# Supplementary material for: Effects of school menstrual hygiene management, water, sanitation, and hygiene interventions on girls’ empowerment, health, and educational outcomes: Lasta district, Amhara regional state, Ethiopia
Source: PLoS One. 2025 Apr 28;20(4):e0321376. doi: 10.1371/journal.pone.0321376 (PMC12036919; doi:10.1371/journal.pone.0321376)
Supplement: S1 Fig — Girls from intervention schools benefited from school peer-to-peer education and teachers who took MHM trainings. This helped girls from intervention school to demonstrate improved hygiene knowledge and practice compared to non-intervention schools who lucked these opportunities. (DOCX) [file pone.0321376.s001.docx]

**S1.Figure**
