# Supplementary material for: Effects of school menstrual hygiene management, water, sanitation, and hygiene interventions on girls’ empowerment, health, and educational outcomes: Lasta district, Amhara regional state, Ethiopia
Source: PLoS One. 2025 Apr 28;20(4):e0321376. doi: 10.1371/journal.pone.0321376 (PMC12036919; doi:10.1371/journal.pone.0321376)
Supplement: S1Table — In non-intervention schools, girls lacked access to dedicated clubrooms where they could change sanitary pads, rest when feeling unwell and exchange hygiene education. This absence significantly impacted their comfort and support for managing menstrual health at school, leading to a much higher proportion of girls refraining from changing sanitary pads during school hours—44% compared to just 4.2% of the girls from intervention schools. (DOCX) [file pone.0321376.s003.docx]

**S1Table**

**Data source questionnaire:** Where do you change sanitary pad at school during menstruation?

| Intervention schools | | | | | | | Non-intervention schools | | | | |
| --- | --- | --- | --- | --- | --- | --- | --- | --- | --- | --- | --- |
|  |  |  |  |  |  |  |  |  |  |  |  |
| Characteristics | In the toilet | In the MHM Club room | In the bush around school | Do not change pad at school due to discomfort | Total | In the toilet | | In the MHM Club room | In the bush around school | Do not change at school due to discomfort | Total |
| Frequency | 60 | 116 | 8 | 8 | 192 | 80 | | 10 | 4 | 74 | 168 |
| Percent | 31.3 | 60.4 | 4.2 | 4.2 | 100 | 47.6 | | 6 | 2.4 | 44 | 100 |
