# Supplementary material for: Effects of school menstrual hygiene management, water, sanitation, and hygiene interventions on girls’ empowerment, health, and educational outcomes: Lasta district, Amhara regional state, Ethiopia
Source: PLoS One. 2025 Apr 28;20(4):e0321376. doi: 10.1371/journal.pone.0321376 (PMC12036919; doi:10.1371/journal.pone.0321376)
Supplement: Attachment — Submitted filename: PONE-D-24-55215.docx [file pone.0321376.s004.docx]

## PLOS ONE

Effects of school menstrual hygiene, water and sanitation interventions on girls’

empowerment, health and educational outcomes: Lasta District, Amhara Regional State, Ethiopia

--Manuscript Draft--

| Manuscript Number: | PONE-D-24-55215 |
| --- | --- |
| Article Type: | Research Article |
| Full Title: | Effects of school menstrual hygiene, water and sanitation interventions on girls’ empowerment, health and educational outcomes: Lasta District, Amhara Regional State, Ethiopia |
| Short Title: | Effects of school based hygiene interventions on girls' empowerment |
| Corresponding Author: | Fisseha Atale Andargie, M.As  Plan International  - please select -, GUYANA |
| Keywords: | girls' empowerment; health(social, emotional and physical); class absenteeism; educational performance; school interventions; hygiene education |
| Abstract: | This study investigated the impact of school-based hygiene education, access to water and sanitation services on menstruating girls' empowerment, health, attendance and academic performance.  The research employed cross-sectional and experimental designs, allowing for comparisons between girls with and without access to these interventions. The study aimed to explore whether such school-based services could foster a supportive environment for menstruating girls, enhancing their ability to manage menstruationrelated needs and reducing absenteeism.  The results demonstrated a marked improvement in confidence among girls at intervention schools, with 54% feeling comfortable purchasing sanitary products at shopping centers compared to 18% in non-intervention schools. Additionally, girls in intervention schools felt more at ease discussing menstruation with boys, with 21% reporting comfort in these conversations versus 8.9% in non-intervention schools. The presence of hygiene facilities also influenced physical health habits, as 51.1% of girls in intervention schools reported practicing genital hygiene three to four times daily, in contrast to 33.3% in non-intervention schools.  Class attendance during menstruation also improved significantly, with 68.3% of girls in intervention schools attending class compared to just 29.8% in non-intervention schools, indicating that better access to water and sanitation services reduces menstruation-related absenteeism.  The findings suggest that implementing hygiene education, access to water and sanitation services in schools can substantially empower menstruating girls, enhance their physical, emotional and social well-being and reduce absenteeism. Despite these improvements, further research is warranted to assess additional strategies that might more directly enhance educational performance among menstruating girls. |
| Order of Authors: | Fisseha Atale Andargie, MA |
|  | Femi Rufus TINUOLA, Ph.D |
| Additional Information: |  |
| Question | Response |
| Financial Disclosure  Enter a financial disclosure statement that describes the sources of funding for the work included in this submission. Review the [submission guidelines](http://journals.plos.org/plosone/s/submission-guidelines#loc-financial-disclosure-statement) for detailed requirements. View published research articles from [PLOS ONE](https://journals.plos.org/plosone/) for specific | The author(s) received no specific funding for this work. |

| examples.  This statement is required for submission and will appear in the published article if the submission is accepted. Please make sure it is accurate. |  |
| --- | --- |
| Funded studies Enter a statement with the following details: - Initials of the authors who received each award - Grant numbers awarded to each author - The full name of each funder - URL of each funder website - Did the sponsors or funders play any role in the study design, data collection and analysis, decision to publish, or preparation of the manuscript? |  |
| Did you receive funding for this work? |  |
| Competing Interests  Use the instructions below to enter a competing interest statement for this submission. On behalf of all authors, disclose any [competing interests](http://journals.plos.org/plosone/s/competing-interests) that could be perceived to bias this work—acknowledging all financial support and any other relevant financial or nonfinancial competing interests.  This statement is required for submission and will appear in the published article if the submission is accepted. Please make sure it is accurate and that any funding sources listed in your Funding Information later in the submission form are also declared in your Financial Disclosure statement.  View published research articles from [PLOS ONE](http://journals.plos.org/plosone/) for specific examples. | I have read the Journal's policy and authors of this manuscript have declared that they have no competing interest. |

| NO authors have competing interests Enter: The authors have declared that no competing interests exist. Authors with competing interests Enter competing interest details beginning with this statement: I have read the journal's policy and the authors of this manuscript have the following competing interests: [insert competing interests here] |  |
| --- | --- |
| * typeset |  |
| Ethics Statement  Enter an ethics statement for this submission. This statement is required if the study involved:   - Human participants - Human specimens or tissue - Vertebrate animals or cephalopods - Vertebrate embryos or tissues - Field research   Write "N/A" if the submission does not require an ethics statement.  General guidance is provided below. Consult the [submission guidelines](http://journals.plos.org/plosone/s/submission-guidelines#loc-guidelines-for-specific-study-types) for detailed instructions. Make sure that all information entered here is included in the Methods section of the manuscript. | The study was a field research that participated girl students' during interviews, questionnaire response and focus group discussions; however it did not include human tissues and vertebrate animals. For the participation of girl students in the research process, ethical clearance letter was obtained from district education office which administers all schools in the district. The ethical clearance letter is uploaded in the 'attach files' section. Moreover, a written consent was obtained from students and this is indicated in the methods section of the manuscript. |

Format for specific study types

Human Subject Research (involving human

participants and/or tissue)

Give the name of the institutional review

board or ethics committee that approved the

study

•

Include the approval number and/or a

statement indicating approval of this

research

•

Indicate the form of consent obtained

(

written/oral) or the reason that consent was

not obtained (e.g. the data were analyzed

anonymously)

•

Animal Research (involving vertebrate

animals, embryos or tissues)

Provide the name of the Institutional Animal

Care and Use Committee (IACUC) or other

relevant ethics board that reviewed the

study protocol, and indicate whether they

approved this research or granted a formal

waiver of ethical approval

•

Include an approval number if one was

obtained

•

If the study involved

non-human primates

,

add

additional details

about animal welfare

and steps taken to ameliorate suffering

•

If anesthesia, euthanasia, or any kind of

animal sacrifice is part of the study, include

briefly which substances and/or methods

were applied

•

Field Research

Include the following details if this study

involves the collection of plant, animal, or

other materials from a natural setting:

Field permit number

•

Name of the institution or relevant body that

granted permission

•

Data Availability

Authors are required to make all data

underlying the findings described fully

available, without restriction, and from the

time of publication. PLOS allows rare

exceptions to address legal and ethical

concerns. See the

[PLOS Data Polic](http://journals.plos.org/plosone/s/data-availability)

[y](http://journals.plos.org/plosone/s/data-availability)

and

[FA](http://journals.plos.org/plosone/s/data-availability#loc-faqs-for-data-policy)

[Q](http://journals.plos.org/plosone/s/data-availability#loc-faqs-for-data-policy)

for detailed information.

Yes - all data are fully available without restriction

| A Data Availability Statement describing where the data can be found is required at submission. Your answers to this question constitute the Data Availability Statement and will be published in the article, if accepted. |  |
| --- | --- |
| Important: Stating ‘data available on request from the author’ is not sufficient. If your data are only available upon request, select ‘No’ for the first question and explain your exceptional situation in the text box. |  |
| Do the authors confirm that all data underlying the findings described in their manuscript are fully available without restriction? |  |
| Describe where the data may be found in full sentences. If you are copying our sample text, replace any instances of XXX with the appropriate details.   - If the data are held or will be held in a public repository, include URLs, accession numbers or DOIs. If this information will only be available after acceptance, indicate this by ticking the box below. For example: All XXX files   are available from the XXX database (accession number(s) XXX, XXX.).   - If the data are all contained within the manuscript and/or Supporting Information files, enter the following: All relevant data are within the manuscript and its Supporting Information files. - If neither of these applies but you are able to provide details of access elsewhere, with or without limitations, please do so. For example:   Data cannot be shared publicly because of [XXX]. Data are available from the XXX Institutional Data Access / Ethics Committee (contact via XXX) for researchers who meet the criteria for access to confidential data.  The data underlying the results presented in the study are available from (include the name of the third party | All relevant data are within the manuscript and its Supporting Information files. |

| and contact information or URL).  • This text is appropriate if the data are owned by a third party and authors do not have permission to share the data.  * typeset |  |
| --- | --- |
| Additional data availability information: |  |

Manuscript	[Click here to access/download;Manuscript;Research Article-](https://www2.cloud.editorialmanager.com/pone/download.aspx?id=38535379&guid=b189996b-c45a-48eb-9d0c-cdb9b6dde129&scheme=1)

[PO.docx](https://www2.cloud.editorialmanager.com/pone/download.aspx?id=38535379&guid=b189996b-c45a-48eb-9d0c-cdb9b6dde129&scheme=1)

1

2

# 3 Effects of school menstrual hygiene, water and sanitation 4 interventions on girls’ empowerment, health and educational 5 outcomes: Lasta District, Amhara Regional State, Ethiopia

6

7

# 8 Effects of school based hygiene interventions on girls’ 9 empowerment

10

## 11 Fisseha A.Andargie^1^*^¶^, Femi R.Tinuola^2^*^&^*

12^1^Department of Public Health Administration, Central University of Nicaragua,
13 Managua,Nicaragua
14 ^2^Department of Sociology, Federal University Gusau, Zamfara State, Nigeria

15

16

17 *Corresponding author E-mail: fissehaatalie@yahoo.com (FA);
18 ^¶^This author contributed to the article 19 *^&^*This author contributed to the article*.*

20

21

22

23

24

25

26

27

28

29

# Abstract

This study investigated the impact of school-based hygiene education, access to water and sanitation services on menstruating girls' empowerment, health, attendance and academic performance.

The research employed cross-sectional and experimental designs, allowing for comparisons between girls with and without access to these interventions. The study aimed to explore whether such school-based services could foster a supportive environment for menstruating girls, enhancing their ability to manage menstruation-related needs and reducing absenteeism.

The results demonstrated a marked improvement in confidence among girls at intervention schools, with 54% feeling comfortable purchasing sanitary products at shopping centres compared to 18% in non-intervention schools. Additionally, girls in intervention schools felt more at ease discussing menstruation with boys, with 21% reporting comfort in these conversations versus 8.9% in non-intervention schools. The presence of hygiene facilities also influenced physical health habits, as 51.1% of girls in intervention schools reported practicing genital hygiene three to four times daily, in contrast to 33.3% in non-intervention schools.

Class attendance during menstruation also improved significantly, with 68.3% of girls in intervention schools attending class compared to just 29.8% in non-intervention schools, indicating that better access to water and sanitation services reduces menstruation-related absenteeism.

The findings suggest that implementing hygiene education, access to water and sanitation services in schools can substantially empower menstruating girls, enhance their physical, emotional and social well-being and reduce absenteeism. Despite these improvements, further research is warranted to assess additional strategies that might more directly enhance educational performance among menstruating girls.

**Keywords:** Girls’ empowerment, health, class attendance, educational performance, school interventions, hygiene education,

## Authors’ summary

Improving girls' education has been a key priority in developing countries, as education equips girls for higher-level positions and decision-making roles. However, traditional attitudes toward education and natural processes, such as menstruation, present significant challenges.

Studies have investigated how menstruation influences girls' class attendance and academic performance, yet findings have been inconsistent—some found that menstruation affects attendance while others reported no impact. Additionally, these studies often focused solely on the school environment, despite girls being influenced by both school and broader social factors. As a result, solid evidence on the extent to which menstruation affects class attendance and performance remains lacking.

Our study employed a comprehensive set of school interventions to examine the effects of menstruation on girls’ attendance and performance. The interventions included access to sex-segregated toilets, water, sanitary products, designated girls' rooms for resting and changing sanitary pads and hygiene education, along with consideration of external environments, like policy and community.

Our findings reveal that comprehensive hygiene and sanitation facilities significantly reduced absenteeism among menstruating girls. Furthermore, the interventions boosted girls' confidence in discussing menstruation with peers, family and teachers, positively influencing their physical, social, and emotional well-being.

## 1. Introduction

Menstruation is a physiological process involving the monthly shedding of the uterine lining, occurring among pubescent girls and pre-menopausal women who are not pregnant [1, 2]. Menstrual hygiene management (MHM) refers to the hygienic handling of menstrual blood using appropriate sanitary products and hygiene practices, including the use of absorbent products, soap, water, and safe disposal of used materials[1, 3].

MHM is an essential component of the human experience; however, discussions around these topics are often deemed sensitive or offensive, particularly in developing nation [4, 5]. Interest in MHM began to emerge in the 2000s when private and non-governmental organizations focused on educating pubescent girls primarily within school settings [6].

In the past two decades, studies have examined the impact of menstruation on girls' class attendance and the influence of the school environment on their hygiene practices [7, 8, 9].

An evaluation conducted by United Nation Children’s Fund (UNICEF), Plan International, Save the Children, and Emory University in the Philippines identified three key determinants of MHM within school environments that can empower girls:- access to water, sanitation and hygiene (WASH) facilities, hygiene education and practical guidance along with access to sanitary products. The evaluation noted that the absence of these determinants could lead to poor MHM practices, confusion, and anxiety related to menstrual leakage, as well as potential teasing from peers [10].

Since 2012, UNICEF and the University of Colombia have been hosting annual conferences focused on the theme "WASH in Schools Empowers Girls' Education." Proceedings from these conferences indicate that school-based WASH interventions have positively influenced girls' education and menstrual hygiene practices [11].

Empowerment is a means through which individuals and groups increase control over their lives, manifesting as self-confidence, self-respect, knowledge and behaviour changes vital for making decisions regarding health and education [12]. However, to date, no study has quantified the impact of school WASH interventions on the empowerment of menstruating girls.

In addition, access to safe drinking water, improved sanitation and hygiene can prevent at least 9.1% of the global disease burden [13] and improvements in health outcomes can lead to enhanced class attendance and academic performance [14, 15, 16].

Conversely, the absence of WASH services can significantly affect emotional, social, and physical health components, resulting in confusion, physical discomfort such as genital bruising, and heightened anxiety due to menstrual leakage, which can lead to teasing from peers [10]. The World Health Organization defines health as a state of complete physical, mental, and social well-being, and not merely the absence of diseases [17].

The relationship between menstruation and class attendance exhibits inconsistencies across different contexts. Studies conducted in Nepal and Malawi reported that menstruation minimally affects girls' class attendance, attributing more significance to factors such as household responsibilities and student motivation [18, 19]. Conversely, researches from Northern Ethiopia and Kenya demonstrate that improvements in hygiene through school interventions positively influence girls' class attendance [8, 20].

Continuous class attendance, along with regular assessments and study time, is positively correlated with educational performance [21]. A study on higher education found that regular attendance reduces the likelihood of poor academic performance [22]. Interestingly, while attendance correlates with lower-performing students, this relationship does not extend to higher-performing students [23].

Generally, previous studies on MHM laid the groundwork for further research but lacked comprehensiveness. Evaluations from Philippine-based NGOs, alongside UNICEF and the University of Colombia’s conference reports, were qualitative assessments that did not incorporate quantitative data [10, 11]. Additionally, no other study has captured the empowering potential of school-based WASH interventions, except for qualitative project completion reports.

Furthermore, existing studies examining impact of menstrual hygiene management on girls’ health have primarily focused on clinical aspects, reproductive tract infections, overlooking critical social, emotional, and physical health dimensions [24]. Research linking MHM practices to class attendance has also been limited, often concentrating solely on in-school factors and neglecting external influences like policies, community dynamics and peer impacts on hygiene practices [7, 8, 9, 17]. However, both school and outside school environments play essential roles in shaping the hygiene practices of menstruating girl students [25].

To address the evident knowledge gap, further studies are essential. The significance of this study, therefore, stemmed from limitations identified in previous researches on MHM. This research therefore aimed to measure the effects of school hygiene, water and sanitation interventions on girls’ self-reliance, health, class attendance and academic performance.

The study examined both in school and out-of-school factors influencing girls' empowerment, health and attendance through school interventions implemented over six years, with active engagement from both girls and teachers. Consequently, the study successfully achieved its aim and objectives.

# 2. Materials and methods

## 2.1 Design and setting

The study employed cross-sectional and experimental designs and was conducted in Lasta District, located in the North Wollo Zone of Amhara Regional State in northern Ethiopia. Six primary second-cycle schools were selected within the district: three schools that had received WASH interventions and three that had not. All schools were situated in rural communities where agriculture is the primary livelihood of local residents.

## 2.2 Description of the school intervention

Plan International Ethiopia, an international non-governmental organization, implemented a menstrual hygiene management (MHM), water, sanitation and hygiene (WASH) program in 10 primary second cycle schools in the Lasta District from July 2018 to January 2024. The intervention aimed to improve menstruating girls' hygiene practices and, in turn, support their school attendance.

Key components of the intervention included MHM education, provision of sanitary products, access to drinking water, gender-segregated toilets and soap. Additionally, the program established dedicated girls’ clubrooms, or MHM rooms, where girls could rest when they feel sick, change sanitary pads, make reusable pads and educate each other on menstruation and menstrual hygiene management.

The Menstrual Hygiene Management (MHM) education process for girls began with Plan International Ethiopia training selected girl and boy students, along with teachers, from each target school. These trained students, referred to as peer trainers, were then responsible for educating other girls in their schools who had not participated in the initial training. This peer-led MHM training continued until all girl students in each target school had been reached. Plan International Ethiopia provided training manuals to support the trainers, who also received guidance from the teachers who had completed the initial MHM training.

WASH experts from Plan International Ethiopia regularly visited the target schools to address emerging challenges faced by girls. For instance, they provided additional sanitary pads when supplies ran low and revitalized girls’ clubs when participation weakened for various reasons. This ongoing support was sustained over the six-year project period, concluding in January 2024.

In contrast, the non-intervention schools did not receive support from Plan International Ethiopia or any other non-governmental organizations. However, as directed by national education policy, all schools in the country maintain co-curricular activities, including a

“Girls' Club” in each school. Occasionally, the government—through the Women and Children Affairs Office—provides training for teachers who oversee these school clubs and activities. While these trainings include some information on menstruation and menstrual hygiene management, their primary focus is on topics like preventing unwanted pregnancies and early marriage. Consequently, girls in the intervention schools experienced a significantly different environment compared to those in the non-intervention schools.

Thus, the aim of the study was to assess the impact of school-based MHM and WASH interventions on girls’ empowerment, health, class attendance and academic performance by comparing outcomes between girls from intervention and non-intervention schools.

The study employed a 95% confidence level and a 5% margin of error, with an expected variance of 0.5. Given the known student population, the research applied finite population formula, detailed as follows [26]:-

𝑆 = 𝑍^2^𝑁𝑃(1 − 𝑃)/𝑒^2^(𝑁 − 1) + 𝑍^2^𝑃(1 − 𝑃)

Where,

S: stands for sample size

N: Population size of students

P: Population proportion, with expected variance of 0.5 Z: Z-score, which is 1.96 in this case; e- Margin of error/degree of accuracy, which is 0.05 (5%);

The total student population across the six schools, including boys, was 1,214. Excluding boys and non-menstruating girls, the study population consisted of 626 pubescent girls. Initially, a sample size of 240 girl students was calculated. However, data collection coincided with the local wedding season when many girls were occupied with family obligations and an uncertain security situation. To reduce the risk of absenteeism during questionnaire administration, the sample size was increased by 19%, adding 120 girls, resulting in a final sample of 360 menstruating girls.

The total sample size was allocated to intervention and non-intervention schools proportionally, based on each group’s share of the total population: 190 girls from intervention schools and 170 girls from non-intervention schools. Luckily, all participants were present for the questionnaire, yielding a 100% response rate.

## 2.3 Informed consent

To ensure ethical compliance, the study secured informed consent from all participants in addition to obtaining ethical clearance from the district education office. Prior to requesting consent, the researcher provided participants with detailed information about the study, including:

- The researchers' identities, contact information and phone numbers.
- The purpose and nature of the study.
- The potential risks and benefits of participation.
- The rationale for selecting the study schools and menstruating girls.
- Assurance of anonymity and respect for their autonomy during the data collection process.

Participants were explicitly informed that they had the right to withdraw from the study at any time. This information was conveyed in a clear manner to ensure participants fully understood the objectives of study and their rights.

An interactive question-and-answer session followed the information briefing. During this session, researchers addressed participants' concerns and queries. This discussion was held before the data collection day—Friday, 8 March 2024—to provide participants ample time to decide on their participation and to minimize delays or fatigue on the actual data collection day.

Subsequently, menstruating girls were asked whether they would voluntarily participate in the study. All participants provided verbal consent. As part of the consent process, participants were also informed that they would be required to provide a signature during questionnaire administration. Thus, a designated signature space was included on the first page of the questionnaire. Accordingly, the informed consent process involved two steps: initial verbal consent followed by written consent.

The study used cross-sectional design and therefore the data collection time was conducted from Monday, 11 March 2024, to Friday, 15 March 2024. These activities included key informant interviews, focus group discussions, observations, and questionnaire administration.

## 2.4 Statistical methods used

Data analysis preparation began with data cleaning, coding of both qualitative and quantitative data, and entering the data into a spreadsheet. Qualitative data was analysed using thematic analysis, while quantitative data was processed with SPSS software. Descriptive statistics, such as frequencies and percentages, were used to summarize respondents' characteristics and responses to each question. To determine statistical significance, the independent samples t-test was extensively applied. Finally, results from both qualitative and quantitative analyses were integrated to determine research hypotheses.

# 3. Results

## 3.1 Sample characteristics

The majority of menstruating girl students were aged 13 to 15 years (68.9%), followed by those aged 16 to 18 years (15.6%) and 10 to 12 years (15.3%). Among the surveyed girls, 97.8% identified as Amhara ethnic, while 1.1% identified as Agew and 0.8% as Tigre. In terms of religious affiliation, 83.9% identified as Orthodox, 9.4% as Protestant, and 6.1% as Muslim.

Parental education levels were generally low, with a higher percentage of fathers able to read and write compared to mothers (66.2% versus 51.7%). Thus, the illiteracy rate among mothers was higher than that of fathers (47.8% compared to 32.8%). Economically, agriculture was the primary source of income for parents, accounting for 81.7% of their livelihoods, while business activities were minimal at just 8.3% (Table 1).

**Table 1: Demographic characteristic of the sample**

| N=360 |  | Characteristics | Frequency | Percent |
| --- | --- | --- | --- | --- |
| Gender |  | Female | 360 | 100 |
| Age |  | 10-12 | 55 | 15.3 |
|  |  | 13-15 | 248 | 68.9 |
|  |  | 16-18 | 56 | 15.6 |
|  |  | Above 19 | 1 | 0.3 |
| Religion |  | Orthodox | 302 | 83.9 |
|  |  | Islam | 22 | 6.1 |
|  |  | Protestant | 34 | 9.4 |
|  |  | Other | 2 | 0.6 |
| Ethnic group |  | Amhara | 353 | 98.1 |
|  |  | Agew | 4 | 1.1 |
|  |  | Tigre | 3 | 0.8 |
| Parents' occupation |  | Farmer | 294 | 81.7 |
|  |  | Merchant | 30 | 8.3 |
|  |  | Office worker | 16 | 4.4 |
|  |  | Other | 20 | 5.6 |
| Mothers' level education | of | Abel to read and write | 144 | 40 |
|  |  | Grade 1-6 | 28 | 7.8 |
|  |  | Grade 7-12 | 14 | 3.9 |
|  |  | Not able to read and write | 172 | 47.8 |
|  |  | Other | 2 | 0.6 |
| Fathers' level education | of | Abel to read and write | 183 | 50.8 |
|  |  | Grade 1-6 | 43 | 11.9 |
|  |  | Grade 7-12 | 14 | 3.9 |
|  |  | Not able to read and write | 118 | 32.8 |
|  |  | Other | 2 | 0.6 |

 The study subjects were only menstruating girl students and so the gender representation is female only.

## 3.2 Empowering girls through water, sanitation and hygiene interventions

### 3.2.1 Qualitative results

Girls from intervention schools reported having open discussions about menstruation and menstrual hygiene management with their mothers, sisters, boys, and teachers. They felt comfortable asking for permission to take breaks during class if they experienced menstruation. A teacher from intervention school and who was supporting the ’’Girls’ Club’’ said, “Girls are not afraid of asking for breaks when menstruation occurs during classes.”

In contrast, girl students from non-intervention schools indicated that their discussions were limited primarily to female teachers. They felt uncomfortable talking about menstruation with boys, who often joked about the topic. A teacher from a nonintervention school remarked, “Girls lack adequate knowledge about menstruation and MHM and they don’t have courage to discuss these topics with teachers and peers.”

### 3.2.2 Quantitative results

Girls from intervention schools demonstrated greater confidence when purchasing sanitary products compared to their peers in non-intervention schools. For example, 54% of girls from intervention schools reported feeling no fear while buying sanitary products, whereas only 18% of the girls from non-intervention schools reported similar confidence. Conversely, 41% of girls in non-intervention schools admitted to feeling fear when purchasing sanitary pads, compared to only 18% of girls from intervention schools who experienced similar feelings (Fig. 1).

Overall, 58% of girls in non-intervention schools reported feeling shy or fearful when buying sanitary products, while only 31% of girls from intervention schools expressed these concerns, indicating that girls from non-intervention schools were nearly twice as likely to experience fear compared to their peers in intervention schools.

0

50

100

150

200

250

I feel

no shy

I feel

shy

I do not

buy

from

shops

I cover

my face

Total

I cover

my face

I feel no

shy

I do not

buy

from

shops

I feel

shy

Total

Intervention schools

Non-intervention schools

127

43

37

30

237

34

36

50

83

203

%

54

18

%

16

%

%

13

%

100

%

17

18

%

25

%

%

41

100

%

**Frequency**

**Girls' experience while buying sanitary products at shops**

Frequency

Percent

**Figure 1**: **Comparing girls’ confidence while purchasing sanitary products**

Quantitative results indicated significant differences in social interactions between girls in the two school environments regarding their relationships with peers and teachers. For instance, 21% of girls from intervention schools reported being able to discuss menstruation and menstrual hygiene management with boys, compared to only 8.9% of girls from nonintervention schools.

Additionally, 22% of girls from intervention/treatment schools felt that menstrual hygiene management (MHM) education enabled them to discuss menstruation with parents and others, while only 9% of girls from non-intervention schools felt the same. Furthermore, 33% of girls in non-intervention schools reported not receiving MHM education while only to just 2% of those in intervention schools (Figure 2).

**Figure 2: Effects of school interventions on girls’ social relations and communications**

*MHM stands for menstrual hygiene management education at schools. Only 2% of the girls from intervention schools reported absence of MHM education in contrast to 33% of the girls from non-intervention schools.*

When the differences in girls’ courage to ask questions, improve communication skills and to discuss with boys and parents on MHM were evaluated using the independent samples t- test, differences were significant where the P-values lie between 0.001 and 0.022. Thus, P<α; 0.001<0.05 and 0.023<0.05 (table2).

**Table 2: T-test results: Impacts of school intervention on girls’ communication and social relations**

| **Independent Samples Test** | |  | | | | |  |  |  | |
| --- | --- | --- | --- | --- | --- | --- | --- | --- | --- | --- |
|  |  | *Levene's Test for*  *Equality of*  *Variances* | | *t-test for Equality of Means* | | |  |  |  | |
|  |  | *F* | *Sig.* | *t* | *df* | *Sig. (2tailed*  *)* | *Mean*  *Differ ence* | *Std.*  *Error Differ ence* | *95% Confidence Interval of the*  *Difference* | |
|  |  |  |  |  |  |  |  |  | *Lower* | *Uppe*  *r* |
| I got courage to ask questions in class | Equal variances assumed | 19.349 | 0.000 | 2.302 | 182 | 0.022 | 0.161 | 0.070 | 0.023 | 0.299 |
|  | Equal variances not assumed |  |  | 2.319 | 181.924 | 0.022 | 0.161 | 0.069 | 0.024 | 0.298 |
| It Helped me improve communicati  on skills | Equal variances assumed | 10.569 | 0.001 | 1.599 | 182 | 0.112 | 0.106 | 0.066 | -0.025 | 0.236 |
|  | Equal variances not assumed |  |  | 1.612 | 181.983 | 0.109 | 0.106 | 0.066 | -0.024 | 0.235 |
| It helped me discuss with  boys on  MHM | Equal variances assumed | 14.256 | 0.000 | 2.543 | 182 | 0.012 | 0.283 | 0.111 | 0.063 | 0.503 |
|  | Equal variances not assumed |  |  | 2.684 | 117.885 | 0.008 | 0.283 | 0.105 | 0.074 | 0.492 |
| It helped me discuss with parents and others on  MHM | Equal variances assumed | 63.424 | 0.000 | 3.573 | 182 | 0.000 | 0.212 | 0.059 | 0.095 | 0.329 |
|  | Equal variances not assumed |  |  | 3.667 | 169.380 | 0.000 | 0.212 | 0.058 | 0.098 | 0.326 |
| No change at  all | Equal variances assumed | 4.993 | 0.027 | -  1.107 | 182 | 0.270 | -  0.042 | 0.038 | -0.117 | 0.033 |
|  | Equal variances not assumed |  |  | -  1.088 | 157.158 | 0.278 | -  0.042 | 0.039 | -0.118 | 0.034 |
| No MHM education at school | Equal variances assumed | 357.730 | 0.000 | -  6.501 | 182 | 0.000 | -  0.341 | 0.053 | -0.445 | -  0.238 |
|  | Equal variances not assumed |  |  | -  6.179 | 103.847 | 0.000 | -  0.341 | 0.055 | -0.451 | -  0.232 |

#### 3.3 Improving girls’ health through school interventions

Clinical data collected from Shumshiha Health Centre, which serves study district communities including girl students from both intervention and non-intervention schools revealed that from September 2022 to February 2024, 41 women visited the centre due to illnesses related to poor menstrual hygiene practices. Among these patients, 14 (34%) were menstruating students. The illnesses were primarily caused by excessive use of sanitary pads and a lack of awareness regarding menstrual hygiene management, as the clinical evidence indicated. This data highlights that girls were at risk for genital bruising due to the overuse of sanitary pads/poor hygiene practices and low level of menstrual hygiene knowledge.

However, the school-based menstrual hygiene management (MHM) and WASH interventions significantly reduced girls' risk of genital bruising by enhancing their hygiene knowledge and practices (Fig. 3). For example, 43.8% of girls in the intervention schools reported practicing genital hygiene three times a day during menstruation, compared to only 19% in non-intervention schools. Additionally, 51.1% of girls from intervention schools practiced genital cleaning three to four times per day against 33.3% of girls from non-intervention schools.

**Figure 3: Comparing daily hygiene practices of girls during menstruation**

In terms of emotional and social aspects of girls’ health, 58% of female students from nonintervention schools reported feeling shy or fearful when buying sanitary products, compared to 31% of those from intervention schools (Fig.1). In addition, 21% of girls from intervention schools reported feeling comfortable discussing menstruation and menstrual hygiene management with boys, compared to only 8.9% of girls from control schools. Furthermore, 22% of girls from intervention schools felt that menstrual hygiene management (MHM) education helped them talk about menstruation with their parents and others, while only 9% of girls from non-intervention schools shared this belief (Fig. 2).

Data from the same health centre, Shumshiha Health Centre, further indicated that waterborne diseases were a significant challenge for the community, including girl students. During the same reporting period, the health centre recorded 125 visits by women due to waterborne diseases, with 51 (41%) of these visits made by girl students.

The most common waterborne illness identified was diarrhoea, primarily caused by a lack of access to improved water and water contamination, as noted by the health centre. This finding aligns with data from the district education office, which revealed that 67% of schools in the district lacked access to improved water sources.

The school WASH intervention, however, significantly improved girls’ access to safe drinking water in the intervention schools. Observations of school facilities and feedback from focus group discussions revealed that, among the three sampled intervention schools, two had access to improved water, while the third school did not have a functioning water system at the time of data collection. In contrast, none of the sampled non-intervention schools had access to improved water.

When water was available at schools, girls reported using it to support safe hygiene practices. For example, 41% of girls from intervention schools used school water for washing sanitary pads and 17% used it for genital cleaning. In comparison, only 24% and 8% of girls from non-intervention schools reported using water for sanitary pad washing and genital cleaning respectively (Fig. 4).

**141**

**59**

**80**

**58**

**8**

**346**

**59**

**19**

**31**

**40**

**93**

**242**

**41**

**%**

**17**

**%**

**23**

**%**

**%**

**17**

**2**

**%**

**100**

**%**

**24**

**%**

**%**

**8**

**13**

**%**

**17**

**%**

**38**

**%**

**100**

**%**

**Menstrual pad washing**

**Genital cleaning**

**Drinking and hand washing**

**Cleaning rooms**

**No water at school**

**Total**

**Menstrual pad washing**

**Genital cleaning**

**Drinking and hand washing**

**Cleaning rooms**

**No water at school**

**Total**

**Intervention schools**

**Non-intervention**

**schools**

**Benefits of school water to menstruating girls**

Frequency

Percent

**Figure 4: Comparing the effects of school interventions on the use of water by girl students**

The difference in the use of school water, when available, for hygiene purpose between two groups of girls was significant when tested through the independent t-test, indicating that all the P-values, except one, had less than 0.05.Thus, P<α; 0.001<0.05(Table 3).

**Table 3: T-test results: Multiple benefits of school water to menstruating girl students**

| **Independent Samples Test** | |  | | | | |  |  |  | |
| --- | --- | --- | --- | --- | --- | --- | --- | --- | --- | --- |
|  |  | Levene's Test for  Equality of  Variances | | **t-test for Equality of Means** | | |  |  |  | |
|  |  | F | Sig. | t | df | Sig. (2tailed) | Mean  Difference | Std. Error  Difference | 95%  Confidence  Interval of the  Difference | |
|  |  |  |  |  |  |  |  |  | Lower | Upper |
| Menstrual pad washing | Equal variances assumed | 5.779 | 0.017 | 5.668 | 182 | 0.001 | 0.386 | 0.068 | 0.252 | 0.52 |
|  | Equal variances not assumed |  |  | 5.639 | 174.45 | 0.001 | 0.386 | 0.068 | 0.251 | 0.521 |
| Genital cleaning | Equal variances assumed | 48.105 | 0 | 3.183 | 182 | 0.002 | 0.19 | 0.06 | 0.072 | 0.308 |
|  | Equal variances not assumed |  |  | 3.256 | 173.37 | 0.001 | 0.19 | 0.058 | 0.075 | 0.305 |
| Drinking and hand washing | Equal variances assumed | 48.809 | 0 | 3.493 | 182 | 0.001 | 0.232 | 0.067 | 0.101 | 0.364 |
|  | Equal variances not assumed |  |  | 3.547 | 180.06 | 0.001 | 0.232 | 0.065 | 0.103 | 0.362 |
| Cleaning rooms | Equal variances assumed | 5.106 | 0.025 | 1.117 | 182 | 0.266 | 0.074 | 0.066 | -  0.056 | 0.204 |
|  | Equal variances not assumed |  |  | 1.123 | 181.63 | 0.263 | 0.074 | 0.065 | -  0.056 | 0.203 |
| No water at school | Equal variances assumed | 407.451 | 0 | -9.438 | 182 | 0.001 | -0.517 | 0.055 | -  0.625 | -  0.409 |
|  | Equal variances not assumed |  |  | -8.999 | 108.45 | 0.001 | -0.517 | 0.057 | -  0.631 | -  0.403 |

## 3.4. Effects of school interventions on girls’ class attendance

### 3.4.1 Qualitative results

Girl students, their mothers and teachers unanimously agreed that the primary causes of girls’ class absenteeism were family responsibilities, such as caring for sick and participating in ceremonies, as well as personal illness. Neither mothers nor girl students cited menstruation as a reason for absenteeism.

Teachers from intervention schools also reported that no pubescent girl students were absent due to menstruation because of the availability of hygiene management services at schools. A female teacher supporting girls’ MHM club at the intervention school remarked, " Menstruating girl students do not miss classes during menstruation because they receive better hygiene services at school than at their homes."

However, teachers from non-intervention schools noted that girl students often-missed classes due to lack of hygiene-supportive services at the school and yet girls were reluctant to disclose their reasons for absence out of fear. Thus, the message was that the availability of hygiene services at schools enhanced girls’ school attendance and vice-versa.

### 3.4.2 Quantitative results

The quantitative results further supported the qualitative evidence regarding girls’ class attendance. When asked about their school attendance during menstruation, 68.3% of girls from intervention schools reported that they often attended school, compared to 29.8% of girls from non-intervention schools who responded similarly (fig5).

For girls from non-intervention schools, the primary causes of class absenteeism, based on the provided options, were lack of sanitary pads (31%) and lack of water at schools (17%). Additionally, 11% cited menstrual pain and discomfort with toilets. In contrast, for girls from intervention schools, the major factors contributing to class absence included menstrual pain (9%), lack of water and sanitary pad (7.8%) and lack of comfortable toilets

(6.4%). The result shows, contrasting causes of girls’ class absenteeism attributed to the school hygiene and WASH interventions (fig.5). Approximately 70.2% of girls from nonintervention schools reported being absent from classes due to menstrual hygiene management (MHM) related challenges, compared to 31.7% of girls from intervention schools.

***149***

***17***

***17***

***14***

***2***

***1***

***218***

***73***

***76***

***42***

***27***

***27***

***245***

***%***

***68***

***%***

***8***

***%***

***8***

***%***

***6***

***%***

***10***

***100***

***%***

***%***

***30***

***1***

***3***

***%***

***17***

***%***

***1***

***1***

***%***

***1***

***%***

***1***

***%***

***100.0***

***0***

***50***

***100***

***150***

***200***

***250***

***300***

***Yes, I often go to school***

***No I do't go to school due to lack of sanitray pads***

***No, I don't go to school due to lack of water***

***No,I don't go to school due to poor toilets***

***No,I don't go to school due to menstrual related…***

***Total***

***Yes, I often go to school***

***No I do't go to school due to lack of pads***

***No, I don't go to school due to due lack of water***

***No,I don't go to school due to poor toilets***

***No,I don't go to school due to menstrual related…***

***Total***

***Intervention schools***

***Non-intervention***

***schools***

***Frequency***

***Girls class attendance during menstruation***

***Frequency***

***Percent***

**Figure5: Class attendance comparison between intervention and non-intervention menstruating girls during menstruation**

When the difference in class absenteeism between girls in intervention and nonintervention schools was evaluated using the independent samples t-test, all characteristics, except one, had p-values between 0.001 and 0.004 which are less than 0.05 (Table 4). Thus, with p < α (0.001 < 0.05; 0.004<0.05), there was a significant difference in class absenteeism during menstruation between the two groups of girl students.

**Table 4: T-test results: Impacts of school intervention on girls’ class attendance**

| **Independent Samples Test** | | | | | | |  |  |  | |
| --- | --- | --- | --- | --- | --- | --- | --- | --- | --- | --- |
|  |  | **Levene's Test for Equality of**  **Variances** | | **t-test for Equality of Means** | | |  |  |  | |
| How often do you go to school during menstruation? |  | F | Sig. | t | df | Sig. (2tailed) | Mean  Difference | Std. Error  Difference | 95% Confidence Interval of the  Difference | |
|  |  |  |  |  |  |  |  |  | Lower | Upper |
| Yes ,I often go to school | Equal variances assumed | 30.100 | 0.000 | 5.104 | 182 | 0.000 | 0.345 | 0.068 | 0.212 | 0.479 |
|  | Equal variances not  assumed |  |  | 5.048 | 167.030 | 0.000 | 0.345 | 0.068 | 0.210 | 0.480 |
| No, I do not go due to lack of  pads | Equal variances assumed | 160.112 | 0.000 | -6.081 | 182 | 0.000 | -0.362 | 0.059 | -0.479 | -0.244 |
|  | Equal variances not  assumed |  |  | -5.886 | 132.432 | 0.000 | -0.362 | 0.061 | -0.483 | -0.240 |
| No, I do not go due to lack of water | Equal variances assumed | 40.802 | 0.000 | -3.022 | 182 | 0.003 | -0.164 | 0.054 | -0.271 | -0.057 |
|  | Equal variances not  assumed |  |  | -2.945 | 144.171 | 0.004 | -0.164 | 0.056 | -0.274 | -0.054 |
| No, I do not go to due to  unclean toilets | Equal variances assumed | 20.063 | 0.000 | -2.164 | 182 | 0.032 | -0.103 | 0.048 | -0.197 | -0.009 |
|  | Equal variances not  assumed |  |  | -2.112 | 146.544 | 0.036 | -0.103 | 0.049 | -0.199 | -0.007 |
| No, I do not go to school, due to menstrual related pain | Equal variances assumed | 3.996 | 0.047 | -0.996 | 182 | 0.321 | -0.051 | 0.051 | -0.151 | 0.050 |
|  | Equal  variances not  assumed |  |  | -0.985 | 168.278 | 0.326 | -0.051 | 0.051 | -0.152 | 0.051 |

- The t-test results show that menstrual pain was the only factor found to be non-significant, suggesting that class absenteeism related to menstrual pain is unaffected by current school interventions and may require additional measures.

Considering both menstrual and non-menstrual period of girls’ class attendance, for girls in non-intervention schools, the top three factors contributing to class absenteeism were family responsibilities (38%), lack of sanitary pads (26%) and menstrual pain and other illnesses (18%). In contrast, for girls in intervention schools, the first three causes of class absence were menstrual pain (30%), family responsibilities (30%), and other illnesses (21%). Notably, girls in intervention schools did not mention lack of sanitary pads, a significant factor for girls in non-intervention schools (Fig. 6).

192

157

59

58

40

168

166

63

43

30

30

%

100

%

82

%

31

%

30

%

21

100

%

99

%

38

%

%

26

18

%

18

%

~~1~~

0

50

100

150

200

250

Toal sample studens in

the category

Total students selected

ranks

Pain related to menses

Family work

Other sickness

Toal sample studens in

the category

Total students selected

ranks

Family work

Lack of sanitary pad

Pain related to menses

Other sickness

Intervention schools

Non-intervention schools

**Frequency**

**Major causes of pubscent girls class absenteeism**

Frequency

Percent

**Figure 6: Comparing top causes of girls’ class absenteeism**

- Most students ,82%, in the intervention schools reported that menstrual related pain is the major cause of class absence while 99% of the girls from non-intervention schools indicated that family work was number one reason for their class absenteeism.

## 3.5 Impacts of class attendance on girls’ performance

### 3.5.1 Girls’ study time at home

The study showed that 77.6% of girls from intervention schools had sufficient time to study at home, compared to only 41.1% of girls from non-intervention schools. Additionally, 53% of girls in non-intervention schools reported lack of adequate study time at home due to family responsibilities, whereas only 10.9% of girls from intervention schools cited the same issue (Figure.7). Thus, the result indicates that girls from intervention schools had more time for studying at home compared to those from nonintervention schools.

481

**Girls’ study time at home**	482

192

200

|  | | |  |  | | | 168 | |
| --- | --- | --- | --- | --- | --- | --- | --- | --- |
| 149 | | |  |  | | |  |  |
|  |  | |  |  | | |  |  |
|  |  | |  | 89 | | |  |  |
|  |  | | 100 | 69 | |  |  | 100 |
|  | 78 | |  |  |  |  |  |  |
|  | 21  12 | |  |  |  | 53 |  |  |
|  |  | 10  11 6 5 |  | 21 ^8^5 | 41 |  |  |  |
| I have I have I often I have enough limited study at enough time to time to library time to study study study due to but no  family interest work | | | Total | I have I often I have I have enough study at enough limited  time to library time to time to study study study but no due to interest family  work | | | Total | |
| Intervention schools | | |  | Non-initervetnion schools | | |  | |

483 175

Frequency

^150^	484

125

100	485

75 486

50

25

487

0

488

489

490

491

Frequency	Percent

492

493 **Figure 7: Comparing menstruating girls’ study time at home:-intervention against non-intervention**
494 **schools**
495 The P-value for the difference in study time was 0.001, which is less than the significance 496 level of 0.05 (P < α; 0.001 < 0.05). This indicates a statistically significant difference in 497 study time between girls in the intervention and non-intervention schools (Table 5).

498	**Table 5: T-test results: Girls study time at home**

Independent Samples Test

|  |  | Levene's Test for Equality of Variances | | t-test for Equality of Means | | |  |  |  |  |
| --- | --- | --- | --- | --- | --- | --- | --- | --- | --- | --- |
| Do you  have enough time to study at home? |  | F | Sig. | t | df | Sig. (2tailed) | Mean Differe nce | Std.  Error Differe nce | 95% Confidence  Interval | |
|  |  |  |  |  |  |  |  |  | Lower | Upper |
|  | Equal variances assumed | 0.846 | 0.358 | -3.522 | 358 | 0.011 | -0.295 | 0.084 | -0.460 | -0.130 |
|  | Equal variances not assumed |  |  | -3.560 | 357.740 | 0.010 | -0.295 | 0.083 | -0.459 | -0.132 |

499

### 500 3.5.2 Girls’ educational performance

To assess the impact of school interventions on girls' academic performance, their firstsemester average scores were collected and compared. Among the 333 girls (menstruating and non-menstruating girls) in the intervention schools, 2% scored "excellent," 4% scored "very good," 14% scored "good," and 29% scored "fair." In comparison, among the 293 girls in non-intervention schools, 3% scored "excellent," 9% scored "very good," 16% scored "good," and 25% scored "fair" (Figure 7). These ratings are based on the standards set by the Ethiopian Ministry of Education for primary and secondary school students.

In summary, 12% of girls from control schools achieved "very good" and "excellent" scores, compared to only 6% of girls in intervention schools, which is less than half the percentage in control schools. However, for scores rated as "good" and "fair," girls from intervention schools led with 43% compared to 41% in control schools.

5

12

48

98

163

8

26

46

74

154

2

4

14

29

100

3

9

16

25

100

0

20

40

60

80

100

120

140

160

180

91-100

81-90

71-80

61-70

61-100

91-100

81-90

71-80

61-70

61-100

Treatment scho

ol

Total

Control school

Total

**Number of students**

**Average score**

**Girl students' first semester average score**

Frequencey

Percent

**Figure 8: Comparing menstruating girls’ first semester educational performance**

Frequencies indicated in blue represent the number of students, while those in red show the percentage of girls within each rating scale who scored at that level. This includes menstruating girls, further divided into those who scored above 60 and those below 61.

## 4. Discussion

Access to adequate hygiene knowledge and management skills was essential for building confidence among girl students. Accordingly, one of the study's hypotheses proposed was that "school MHM and WASH interventions empower girl students."

Qualitative results from focus group discussions with mothers, girl students, and teachers indicated that girls from intervention schools felt more confident in discussing menstruation and hygiene management with peers, teachers, and mothers compared to girls from non-intervention schools.

Quantitative findings revealed that 54% of girls in intervention schools felt confident buying sanitary products compared to 18% in non-intervention schools; while 41% of non-intervention school girls felt fear versus 18% in intervention schools (Fig. 1). Similarly, 21% and 22% of intervention school girls felt confident discussing menstruation with boys and parents respectively compared to 8.9% and 9%of the girls from in non-intervention schools in the same order (Fig. 2).

The difference between intervention and non-intervention schools girls in self - confidence was statistically significant when tested using the independent samples t-test, showing a P-value of 0.001, which is less than 0.05. Thus, as P < α (0.001 < 0.05), the null hypothesis was rejected, and the alternative hypothesis was accepted. Accordingly, there was a significant difference in the empowerment of girl students between intervention and nonintervention schools (Table 2).

The observed difference in girls' empowerment was attributed to the impact of school hygiene and WASH interventions. The interventions enhanced girls' hygiene knowledge through MHM education and provided opportunities to discuss menstruation with boys, teachers, peers and parents. As a result, girls in intervention schools demonstrated increased confidence, reflected in their social interactions with peers, teachers, and parents, and in their emotional control when purchasing sanitary products at shops.

The findings align with an assessment report from UNICEF, Plan International, Save the Children, and Emory University, based in the Philippines, which identified WASH facilities, knowledge, practical guidance, and access to sanitary products as essential services that empower girls [10]. These institutions reported that the lack of these services lead to unsafe hygiene practices, confusion, anxiety about staining clothes with blood and teasing by boys.

The consecutive proceedings of the United Nation Children’s fund (UNICEF) and the

University of Columbia on WASH over the past ten years have also shown that school WASH interventions are empowering girls' education [11]. Furthermore, studies focusing on MHM and class attendance in Niger and Uganda [5, 27] emphasized the importance of school infrastructure, MHM knowledge and access to WASH services to effect profound changes among adolescent girls in menstrual hygiene knowledge and practices.

The findings of this study on girls’ empowerment through school interventions, which included a comprehensive package of hygiene services in the school environment, appear to be unique. The findings may contribute to filling the knowledge gap identified in previous studies, which primarily focused mainly on the effects of menstruation on girls’ class attendance.

In addition, women’s empowerment has been a prominent global issue, particularly in developing countries. The findings of this study suggest that school hygiene and WASH interventions can serve as effective approach to empower girl students, who represent future leaders. Finally, the finding may inform local government budget decisions regarding school interventions and encourage non-governmental organizations to enhance efforts in school hygiene and WASH interventions.

**Enhancing Girls’ Health through School Interventions:** Lack of school-based MHM education and WASH interventions leads to unsafe hygiene practices, confusion, and anxiety among girl students, negatively affecting their physical, emotional, and social health [10]. Accordingly, the hypothesis for this section of the study was that "school MHM and WASH interventions positively impact the health of girl students."

Based on the study district health centre data from September 2022 to February 2024 regarding menstrual hygiene and waterborne diseases, overuse of sanitary products and limited knowledge of menstruation and hygiene management were associated with genital bruising and menstrual hygiene-related discomfort among women and girl students. During this period, 34% of health centre visitors were girl students.

However, the school MHM and WASH interventions improved girls’ knowledge and hygiene practices, positively influencing their health. For example, 43.8% of girls in intervention schools practiced genital hygiene three times daily during menstruation, compared to only 19% of girls in control/non-intervention schools. Additionally, 51.1% of girls in intervention schools practiced genital cleaning three to four times daily, while only 33.3% of girls in non-intervention schools did so (Fig. 3).

In terms of emotional health, 58% of female students in non-intervention schools reported feeling shy or fearful when purchasing sanitary products, compared to 31% in intervention schools. This finding suggests that the school intervention reduced fears among girls in intervention schools by nearly half compared to their peers in non-intervention schools (Fig. 1).

Furthermore, the intervention enhanced girls' communication and social interactions with peers and teachers (social health). For instance, 21% of girls in treatment/intervention schools reported being comfortable discussing menstruation and menstrual hygiene management with boys, compared to only 8.9% of girls in non-intervention schools. Additionally, 22% of girls in intervention schools felt that MHM education enabled them to discuss menstruation with their parents and others, whereas only 9% of girls in nonintervention schools felt confident enough to engage in such discussions (Fig. 2).

The intervention enabled most schools to access water, which girl students used to enhance safe hygiene practices. For instance, 41% of girls in intervention schools used water for washing sanitary pads and 17% for genital cleaning, compared to 24% and 8% of girls in non-intervention schools, respectively (Fig. 4).

The use of water for safe hygiene practices among girl students revealed a significant difference between intervention and non-intervention school with P values less than 0.05

(P < α; 0.001 < 0.05),(Table 3), indicating a statistically significant difference and supporting the rejection of the null hypothesis in favour of the alternative one.

The findings align with the studies from Bangladesh and Nepal, which reported that access to menstrual hygiene education and WASH services reduces stress and discomfort among girls while improving hygiene practices [3]. Similarly, a research finding in India indicated that access to WASH services and enhanced hygiene practices help mitigate negative health impacts, such as infections associated with reusable pads [24].

However, the health centre report in the study district, identifying diarrhoea as WASHrelated challenge-affecting students, including girls, was not supported by both the qualitative and quantitative findings of this study. Girls, in fact, ranked diarrhoea as the least significant cause of their class absenteeism (Fig. 6).

Girls and mothers’ response, however, might have been influenced by cultural factors, as "diarrhoea" carries a negative connotation in the community, where the local term for it is often used as an insult. This cultural stigma may have led focus group participants to feel hesitant to address this issue directly.

Another possible explanation is that mothers and girls may not have distinguished diarrhoea as a specific health concern, rather using the more generic term "sickness" instead. This may explain the rankings provided by girls regarding their absenteeism (Fig. 6). In both intervention and non-intervention schools, girls ranked "other types of sickness" fourth, following family responsibilities, lack of sanitary pads, and menstrual hygiene-related pains among six potential absenteeism causes. This suggests that diarrhoea may have been overlooked or subsumed under the broader label of "other sickness."

The findings regarding the impact of WASH intervention on girls' health underscore that school initiatives have the potential to enhance the emotional, social and physical wellbeing of girls. This highlights the importance of implementing school hygiene and WASH interventions.

**Effects of School Interventions on Class Attendance:** The effects of menstruation on girls’ class absenteeism have been a prominent topic of study since menstrual hygiene management (MHM) emerged as a research focus. However, robust evidence on the extent to which menstruation impacts girls' class attendance remains limited [28]. Thus, this study also included a research hypothesis stating, "School MHM and WASH interventions have an impact on or improve girls' class attendance."

Qualitative results indicated that menstruation was not a primary reason for girls’ absenteeism. Instead, absenteeism was more closely linked to family responsibilities, such as caregiving when a family member is sick, as well as the girls' own health issues.

However, mothers from non-intervention schools noted that lack of hygiene management services, such as access to water and sanitary pads, posed significant challenge for girls during menstruation. Additionally, girls from non-intervention schools mentioned, “Most girl students do not change sanitary pads at school due to a lack of access to water, sanitary pads and girl-friendly toilets."

In contrast, girls from intervention schools stated, "Girls change sanitary pads in both the sex-segregated toilets and girls’ club rooms. A teacher facilitating the MHM Club at the intervention school also added, "During menstruation, girls prefer to come to school because they receive better hygiene services here than they do at home."

Conversely, a principal and MHM club facilitator from a non-intervention school acknowledged the absence of hygiene services to support girls' needs, saying, "Although girls do not explicitly tell us they are absent due to menstruation, we believe that inadequate school hygiene services contribute to their absenteeism."

The qualitative results indicate that while menstruation itself may not directly cause absenteeism, the lack of adequate hygiene services in schools does contribute to girls’ absences during this time. Therefore, it is crucial to recognize that menstruation cannot be viewed in isolation from the availability of school hygiene services.

Quantitative results further clarified qualitative findings. For instance, 68.3% of girls from intervention schools reported attending school during menstruation, compared to only 29.8% from non-intervention schools. About 48% of girls from non-intervention schools acknowledged that they did not attend school during menstruation due to a lack of sanitary products and water, compared to only 15.6% of girls in intervention schools (Fig. 5).

Girls' absenteeism was also linked to social relationships, as teasing from boys during menstruation could increase absences. For example, 21% of girls in intervention schools reported feeling comfortable discussing menstruation and menstrual hygiene with boys, compared to only 8.9% of girls in non-intervention schools. Additionally, 21.7% of girls in intervention schools felt that MHM education helped them discuss menstruation with parents and others, while only 9.2% of girls in non-intervention schools shared this confidence (Fig. 2).

Thus, the qualitative findings underscore the importance of both the physical and social environments in schools for supporting girls’ hygiene practices. Meanwhile, the quantitative results provided measurable insights into how these factors influence girls’ class attendance.

The difference in attendance between girls from intervention and non-intervention schools was statistically significant (Table 4), with a P-value of 0.001, which is less than 0.05.

Therefore, the null hypothesis was rejected, and the alternative hypothesis was accepted.

This finding aligns with the North Ethiopia Tigray Region, study finding which demonstrated that providing sanitary pads and MHM education improved girls' class attendance [8]. Similarly, a study in Ghana found a significant increase in attendance after providing sanitary products and adolescent education [14]. A systematic review on the impacts of school water and sanitation also concluded that the absence of water and sanitation services contributes to absenteeism during menstruation [16]. Furthermore, a study in Kenya found that access to school WASH services significantly reduced absenteeism among primary school students [20].

Conversely, the finding of this study contrasts with two other studies. For instance, a research finding in Nepal found that girls’ absenteeism did not decrease with the provision of menstrual cups [9]. Similarly, a study in Malawi reported that varying levels of WASH service access did not significantly affect absenteeism among girls in different schools [19].

However, the contexts of Nepal and Malawi studies differ significantly from this study. The Nepal study focused solely on menstrual cup provision without addressing other hygiene services or social factors within and outside the school environment that can significantly influence girls’ hygiene behaviours. Similarly, the Malawi study did not consider behaviour change promotion related to the use of school WASH services, access to and use of sanitary products and outside school environments.

This study demonstrates the need for adopting a comprehensive and targeted hygiene and WASH intervention to effectively promote behaviour change among female students. In schools where adequate hygiene services are lacking, menstruation can contribute to increased absenteeism. Thus, it is crucial to evaluate menstruation's impact on absenteeism in relation to the availability of hygiene services within the school. Furthermore, as female students are affected by social dynamics both within and outside the school environment, it is important to consider their external contexts when examining hygiene practices and attendance.

School interventions not only influenced girls’ class attendance but also positively affected their study time at home. Although intervention and non-intervention schools are situated in the same rural communities, girls in intervention schools felt confident discussing and persuading their parents to reduce household tasks and allow more study time, unlike their counterparts who were less confident. Ultimately, while it may not be possible to eliminate all factors contributing to girls' absenteeism, effective school interventions can significantly reduce menstruation-related absences.

**Impacts of Class Attendance on Academic Performance:-**Based on the preceding discussions, the findings indicate that school interventions significantly improved girls' class attendance/reduced menstruation related absenteeism significantly. Therefore, the research hypothesis for this subtopic was, "School MHM and WASH interventions, through improved class attendance, positively influence girls' academic performance**.**"

The study revealed that, in addition to increased class attendance, the school intervention significantly enhanced girls' study time at home. For instance, 77.6% of girls from intervention schools reported having sufficient time to study at home, compared to only

41.1% of girls from non-intervention schools. Additionally, 53% of girls in nonintervention schools reported a lack of study time due to family responsibilities, whereas only 10.9% of girls in intervention schools faced the same issue (Fig. 7).

The t-test results indicated a statistically significant difference in study time between girls from intervention and non-intervention schools, with a p-value of 0.001, which is significant at the 0.05 level (Table 5). The next question, therefore, is whether these improvements in class attendance and study time for girls in intervention schools translated into improved academic performance.

To evaluate and compare girls’ academic performance between the two categories of schools, the researcher analysed students' average scores from the first semester and used rating scales such as "excellent," "very good," "good," and "fair." In intervention schools, the percentages of girls receiving these ratings in order were 2%, 4%, 14%, and 29%, respectively, out of 333 students. In comparison, in the non-intervention schools girls had the ratings of 3%, 9%, 16%, and 25% with similar order from the 293 students across the three non-intervention schools (Fig. 7).

In summary, 12% of girls from non-intervention schools received "very good" and "excellent" ratings, while only 6% of girls from intervention schools achieved these ratings, less than half the percentage observed in non-intervention schools. However, for the ratings of "good" and "fair," girls from intervention schools outperformed their peers, with 43% achieving these ratings compared to 41% in non-intervention schools.

These findings contrast with prior research that established a positive correlation between class attendance and academic performance [21, 22]. For instance, a study titled "Class Attendance and Learning Outcomes" found that attendance was positively associated with improved performance among low-achieving students [23]. This observation partially aligns with the results for low-performing girls in intervention schools, where 43% received "good" and "fair" ratings compared to 41% in control schools; however, this difference is not statistically significant.

The study results suggest that high-performing students were largely unaffected by class attendance, while the impact of attendance on low-performing students was minimal. This could be due to the fact that the comparison relied solely on first-semester average scores. Differences may have been more pronounced if performance had been analysed over an entire academic year.

Furthermore, the number of high-performing students differed between the two categories of schools, with more students achieving "excellent" and "very good" ratings in nonintervention schools compared to intervention schools. This discrepancy suggests that factors beyond class attendance may also contribute to academic performance.

# 5. Conclusion

The school intervention study demonstrated that school hygiene, water and sanitation (WASH) interventions have numerous positive effects on female students. The interventions significantly boosted their confidence in discussing menstruation and lowered anxieties. Beyond fostering a supportive school environment, the interventions positively influenced girls’ emotional, social, and physical well-being. Additionally, the study found that school interventions could reduce menstruation-related absenteeism, although less effective in directly improving academic performance.

Therefore, this research provides valuable insights into the challenges faced by female students and stands out from previous studies due to its use of long-term school interventions and exploration of new dimensions such as empowerment, health, and educational performance. By addressing key knowledge gaps, the findings hold promise to encourage local government decision-makers and non-governmental organizations to further advance school hygiene and WASH initiatives. In addition, in sights on girls’ empowerment, physical, emotional and social aspects of health may trigger researchers to expand the area of research, besides the most commonly researched theme, ‘’effects of menstruation on girls’ class attendance’’.

**Acknowledgements:** The authors recognize the contribution made by Ato Yalew Tizazu and Wondemu Sisay who supported the research during data collection.

# 6. References

1. Ethiopian Ministry of Health. National Adolescent and Youth Health Strategy.2018; Available from[:https://www.prb.org/wp-content/uploads/2018/05/National-Adolescent-and-YouthHealth- Strategy](https://www.prb.org/wp-content/uploads/2018/05/National-Adolescent-and-Youth-Health-%20Strategy)
2. Crofts T, Coates S, FisherJ. Menstruation hygiene management for school girls. 2014; Available from: <http://wedc.lboro.ac.uk/wedc.lboro.ac.uk/knowledge>
3. Bhusal CK. Practice of menstrual hygiene and associated factors among adolescent school girls in	Dang	District,	Nepal.	*Advances	in	Preventive	Medicine*.2020; http://doi:10.1155/2020/1292070.
4. Tamiru S, Mamo K, Acidria P, Mushi R, Ali CS, Ndebele L. Towards a sustainable solution for school menstrual hygiene management: Cases of Ethiopia, Uganda, South-Sudan, Tanzania, and Zimbabwe. Sahin M, editor. Waterlines.2015;34:http://*doi.org/10.3362/17563488.2015.009*
5. Seydou F, Ama-dou B, Abdoulaye S, Alassane T, Oumar TS, Ibrahima K, et al. Menstrual hhygiene mmanagement in school ssetting in two ssecondary sschools in the Bamako District, Case of the School “La Chaine Grise” and the sschool cheick Modibo Diarra. 2020; http:// doi.org/10.4236/ojog.2020.1020025
6. Sommer M, Hirsch JS, Nathanson C, Parker RG. Comfortably, Safely, and Without Shame:

Defining Menstrual Hygiene Management as a Public Health Issue. American Journal of Public Health. 2015; <https://ajph.aphapublications.org/doi/full/10.2105/AJPH.2014.302525>

7. Tegegne TK, Sisay MM. Menstrual hygiene management and school absenteeism among female adolescent students in Northeast Ethiopia. BMC Public Health. 2014;14:http// [doi/10.1186/1471-2458-14-1118](https://doi/10.1186/1471-2458-14-1118)
8. Belay S, Kuhlmann AKS, Wall LL. Girls’ attendance at school after a menstrual hygiene intervention in northern Ethiopia. International Journal of Gynecology & Obstetrics. 2020; 149:287–91. http:// doi: 10.1002/ijgo.13127
9. Oster E, Thornton R. Menstruation, Sanitary Products, and School Attendance: Evidence from a Randomized Evaluation. American Economic Journal: Applied Economics. 2011; 3: 91–100. http://doi: 10.1257/app.3.1.91

10.Haver,J,Caruso, BA, Ellis, A, Sahin, M, Villasenor, JM, Andes, KL, Freeman MC. WASH in

Schools Empowers Girls’ Education in Masbate Province and Metro Manila, Philippines: An assessment of menstrual hygiene management in schools.2013; avaialbel from [https://www.unicef.org/wash/schools.](https://www.unicef.org/wash/schools)

11. United Nations Children’s Fund and Columbia University. WASH in schools empowers girls’ education: Proceedings of the annual virtual conference on menstrual hygiene management in schools.2017; available from https://www.washinschoolsindex.com

[/storage/articles/ck1he0Zuxts5R7dNgMiPtrRF2SuCKjP54b3VbjnA.](https://www.washinschoolsindex.com/storage/articles/ck1he0Zuxts5R7dNgMiPtrRF2SuCKjP54b3VbjnA.)

12. Woodall J, Raine G, South J, Warwick-Both L. Empowerment , health and well-being. 2010; available from <https://core.ac.uk/download/pdf/42412714.pdf>
13. Prüss-Üstün A, World Health Organization. Safer water, better health: Costs, benefits and sustainability of interventions to protect and promote health. 2008; available from <https://apps.who.int/iris/handle/10665/43840>
14. Samuel KA, Ebenezer EYA, Nang BD, George O, Charles CA, et al. WASH in selected basic schools and possible implications on health and academics: An example of the Wa

Municipality of Ghana, West Africa. American Journal of Environmental Science and

Engineering. 2021; 5:15-20. http:// [doi.org/10.11648/j.ajese.20210501.13](https://doi.org/10.11648/j.ajese.20210501.13)

15.Birdthistle I, Dickson K, Freeman M, Javidi L. What impact does the provision of separate toilets for girls at schools have on their primary and secondary school enrolment, attendance and completion? A systematic review of the evidence. EPPI-Centre, Social Science Research Unit, Institute of Education, University of London. 2011; ISBN: 978-907345: available from https;//WWW.educ-links.org

16. Jasper C, Le T-T, Bartram J. Water and sanitation in schools: A systematic review of the health and educational outcomes. Research Gate. 2012; http:// doi 10.3390/ijerph9082772.

17.*World Health Organization. Basic Documents,49ed. 2020; available from* [*https://apps.who.int/gb/bd/pdf_files/B*](https://apps.who.int/gb/bd/pdf_files/BD_49th-en.pdf%20.V12)	*D_49th-en.pdf	.V12*

18. Oster E, Thornton R. Menstruation, sanitary products, and school attendance: Evidence from a randomized evaluation. American Economic Journal.2011;3:91–100.http://doi: 10.1257/app.3.1.91
19. Grant MJ, Lloyd CB, Mensch BS. Menstruation and school absenteeism: Evidence from rural Malawi. Comparative Education Review. 2013; http:// doi: 10.1086/669121
20. Freeman MC, Greene LE, Dreibelbis R, Saboori S, Muga R, Brumback B, et al. Assessing the impact of a school-based water treatment, hygiene and sanitation programme on pupil absence in Nyanza Province, Kenya: A cluster-randomized trial. Tropical Medicine & International Health. 2011; http:// doi:10.1111/j.1365-3156.2011.02927.x
21. [Tetteh GA.](https://www.emerald.com/insight/search?q=Godson%20Ayertei%20Tetteh) Effects of classroom attendance and learning strategies on the learning Outcome. *Journal of International Education in Business*. 2018; 11: http:// [doi.org/10.1108/JIEB-01-20170004](https://doi.org/10.1108/JIEB-01-2017-0004)
22. Al Hazaa K , Abdel-Salam,ASG, Ismail R, Johnson C, Al-Tameemi R A N, Romanowski MH, etal. The effects of attendance and high school GPA on student performance in first-year undergraduate courses. *Cogent Education. 2021;8*: <https://doi.org/10.1080/2331186X.2021.1956857>
23. *Kwak DW, Sherwood C, Tang KK*. [**C**lass attendance and learning outcom**e**:](https://ideas.repec.org/a/spr/empeco/v57y2019i1d10.1007_s00181-018-1434-7.html) [Empirical economics](https://ideas.repec.org/s/spr/empeco.html) 2019; 57: 177-203. http://doi: 10.1007/s00181-018-1434-7
24. Das P, Baker KK, Dutta A, Swain T, Sahoo S, Das BS, et al. Menstrual hygiene practices, WASH access and the risk of urogenital infection in women from Odisha, India. Journal.pone.2015; http:// doi: 10.1371/journal.pone.0130777: PMC4488331.
25. Glanz K, Rimer BK, Viswanath K. Health behaviour and health education: Theory, research and practice. **Jo**ssey-Bass.2008; <https://psycnet.apa.org/fulltext/2008-17146-000.pdf>
26. Krejcie RV, Morgan DW. Determining sample size for research activities: Educational and psychological measurement. Sage publications.1970;30:607–10.http:// doi/10.1177/001316447003000308
27. Miiro G, Rutakumwa R, Nakiyingi-Miiro J, Nakuya K, Musoke S, Namakula J, et al. Menstrual health and school absenteeism among adolescent girls in Uganda: A feasibility study. BMC Women’s Health. 2018; 18: http:// doi 10.1186/s12905-017-0502-z
28. Sumpter C, Torondel B. A systematic review of the health and social effects of menstrual hygiene management. PLoS ONE. 2013;8: http:// doi:10.1371/journal.pone.0062004

## 7. Supporting documents

### S1 Figure. *Girls’ hygiene knowledge comparison between two groups of schools*

Girls from intervention schools benefited from school peer-to-peer education and teachers who took MHM trainings. This helped girls from intervention school to demonstrate improved hygiene knowledge and practice compared to non-intervention schools who lucked these opportunities.

### S2 Figure: Genital hygiene practice comparison between intervention and non-intervention girls during menstruation S3 Figure: Menstruating girls’ confidence to request menstrual break during classes

Menstruating girls from intervention school improved their social relations with boys and teachers due to the school intervention and this was reflected in their confidence to request their teachers, male and female, for menstrual break during classes, unlike the nonintervention school girls who were limited to female teachers***.***

### *S1Table: Comparing* girls’ experience to change sanitary pads at schools

In non-intervention schools, girls lacked access to dedicated clubrooms where they could change sanitary pads, rest when feeling unwell and exchange hygiene education. This absence significantly impacted their comfort and support for managing menstrual health at school, leading to a much higher proportion of girls refraining from changing sanitary pads during school hours—44% compared to just

4.2% of the girls from intervention schools.

S1-Fig.tif

[Click here to access/downloa](https://www2.cloud.editorialmanager.com/pone/download.aspx?id=38302684&guid=a81229ae-8e55-4cc1-89a9-b01a580edc36&scheme=1)

[d](https://www2.cloud.editorialmanager.com/pone/download.aspx?id=38302684&guid=a81229ae-8e55-4cc1-89a9-b01a580edc36&scheme=1)

[Supporting Informatio](https://www2.cloud.editorialmanager.com/pone/download.aspx?id=38302684&guid=a81229ae-8e55-4cc1-89a9-b01a580edc36&scheme=1)

[n](https://www2.cloud.editorialmanager.com/pone/download.aspx?id=38302684&guid=a81229ae-8e55-4cc1-89a9-b01a580edc36&scheme=1)

[Fig1.ti](https://www2.cloud.editorialmanager.com/pone/download.aspx?id=38302684&guid=a81229ae-8e55-4cc1-89a9-b01a580edc36&scheme=1)

[f](https://www2.cloud.editorialmanager.com/pone/download.aspx?id=38302684&guid=a81229ae-8e55-4cc1-89a9-b01a580edc36&scheme=1)

S2-fig.tif

[Click here to access/downloa](https://www2.cloud.editorialmanager.com/pone/download.aspx?id=38302693&guid=72af43f2-81ed-4f3d-bca6-7dcb168869f0&scheme=1)

[d](https://www2.cloud.editorialmanager.com/pone/download.aspx?id=38302693&guid=72af43f2-81ed-4f3d-bca6-7dcb168869f0&scheme=1)

[Supporting Informatio](https://www2.cloud.editorialmanager.com/pone/download.aspx?id=38302693&guid=72af43f2-81ed-4f3d-bca6-7dcb168869f0&scheme=1)

[n](https://www2.cloud.editorialmanager.com/pone/download.aspx?id=38302693&guid=72af43f2-81ed-4f3d-bca6-7dcb168869f0&scheme=1)

[Fig2.ti](https://www2.cloud.editorialmanager.com/pone/download.aspx?id=38302693&guid=72af43f2-81ed-4f3d-bca6-7dcb168869f0&scheme=1)

[f](https://www2.cloud.editorialmanager.com/pone/download.aspx?id=38302693&guid=72af43f2-81ed-4f3d-bca6-7dcb168869f0&scheme=1)

S3-fig.tif

[Click here to access/downloa](https://www2.cloud.editorialmanager.com/pone/download.aspx?id=38302695&guid=9352a661-1f7d-4932-9345-f5bebf4fd609&scheme=1)

[d](https://www2.cloud.editorialmanager.com/pone/download.aspx?id=38302695&guid=9352a661-1f7d-4932-9345-f5bebf4fd609&scheme=1)

[Supporting Informatio](https://www2.cloud.editorialmanager.com/pone/download.aspx?id=38302695&guid=9352a661-1f7d-4932-9345-f5bebf4fd609&scheme=1)

[n](https://www2.cloud.editorialmanager.com/pone/download.aspx?id=38302695&guid=9352a661-1f7d-4932-9345-f5bebf4fd609&scheme=1)

[Fig3.ti](https://www2.cloud.editorialmanager.com/pone/download.aspx?id=38302695&guid=9352a661-1f7d-4932-9345-f5bebf4fd609&scheme=1)

[f](https://www2.cloud.editorialmanager.com/pone/download.aspx?id=38302695&guid=9352a661-1f7d-4932-9345-f5bebf4fd609&scheme=1)

S1-table.tif

[Click here to access/downloa](https://www2.cloud.editorialmanager.com/pone/download.aspx?id=38302708&guid=417f5988-3aa1-4f70-ad43-f226dfccab83&scheme=1)

[d](https://www2.cloud.editorialmanager.com/pone/download.aspx?id=38302708&guid=417f5988-3aa1-4f70-ad43-f226dfccab83&scheme=1)

[Supporting Informatio](https://www2.cloud.editorialmanager.com/pone/download.aspx?id=38302708&guid=417f5988-3aa1-4f70-ad43-f226dfccab83&scheme=1)

[n](https://www2.cloud.editorialmanager.com/pone/download.aspx?id=38302708&guid=417f5988-3aa1-4f70-ad43-f226dfccab83&scheme=1)

[Fig4.ti](https://www2.cloud.editorialmanager.com/pone/download.aspx?id=38302708&guid=417f5988-3aa1-4f70-ad43-f226dfccab83&scheme=1)

[f](https://www2.cloud.editorialmanager.com/pone/download.aspx?id=38302708&guid=417f5988-3aa1-4f70-ad43-f226dfccab83&scheme=1)
