# Supplementary material for: Effects of school menstrual hygiene management, water, sanitation, and hygiene interventions on girls’ empowerment, health, and educational outcomes: Lasta district, Amhara regional state, Ethiopia
Source: PLoS One. 2025 Apr 28;20(4):e0321376. doi: 10.1371/journal.pone.0321376 (PMC12036919; doi:10.1371/journal.pone.0321376)
Supplement: Attachment — Submitted filename: R Comment.docx [file pone.0321376.s005.docx]

I appreciate the work addressing this timely public health issue. I would like to raise few concerns regarding the study:

1. **Method section**

You mentioned that you utilized both methods. Could you clarify the purpose of the cross-sectional study? Additionally, what type of experimental design did you implement (e.g., true randomization, quasi-experiment, longitudinal, etc.)?

2. **Details of the Intervention**
   The description of the intervention lacks specificity in terms of **quantity, frequency, and duration**. Additionally, the content of the intervention is described too superficially, making it difficult to derive meaningful insights or formulate policy recommendations based on the findings.
3. **Contamination**
   The manuscript does not clearly outline the strategies implemented to prevent contamination or to buffer against potential biases.
4. **Ethical Considerations**
    It is important to understand how the researchers addressed ethical dilemmas faced by non-intervention schools. Clarification on this matter is necessary.

Students under the age of 18 are included in your study. In this case, you must obtain both assent from the students and consent from their parents. Why was not assent considered?

5. **Quality Control Mechanisms**
   What quality control mechanisms were in place to ensure the fidelity of the intervention, particularly regarding educational components? Blinding, randomization and other quality control mechanisms, needs to be clearly articulated.
6. **Mixed Methods Approach**
   The implementation of mixed methods is noted. Please clarify the sequence of the qualitative and quantitative studies: which method was conducted first, and what was the rationale behind this choice?
7. **Controlling for Confounding Factor**

Given that school performance can be influenced by multiple factors, it is essential to explain how the study controlled for the effects of other confounding variables on both school attendance and educational performance.

I recommend that all these concerns be addressed in the body of the manuscript to enhance its clarity and robustness.
