# Supplementary material for: Effects of school menstrual hygiene management, water, sanitation, and hygiene interventions on girls’ empowerment, health, and educational outcomes: Lasta district, Amhara regional state, Ethiopia
Source: PLoS One. 2025 Apr 28;20(4):e0321376. doi: 10.1371/journal.pone.0321376 (PMC12036919; doi:10.1371/journal.pone.0321376)
Supplement: Attachment — Submitted filename: Response to Reviewers.docx [file pone.0321376.s008.docx]

PONE-D-24-55215

Effects of school menstrual hygiene, water and sanitation interventions on girls’ empowerment, health and educational outcomes: Lasta District, Amhara Regional State, Ethiopia

PLOS ONE

Dear Dr. Andargie,

Thank you for submitting your manuscript to PLOS ONE. After careful consideration, we feel that it has merit but does not fully meet PLOS ONE’s publication criteria as it currently stands. Therefore, we invite you to submit a revised version of the manuscript that addresses the points raised during the review process.

All three reviewers have provided extensive comments to the writing of the manuscript which need to be addressed.

Please submit your revised manuscript by Feb 21 2025 11:59PM. If you will need more time than this to complete your revisions, please reply to this message or contact the journal office at [plosone@plos.org](mailto:plosone@plos.org). When you're ready to submit your revision, log on to https://www.editorialmanager.com/pone/ and select the 'Submissions Needing Revision' folder to locate your manuscript file.

Please include the following items when submitting your revised manuscript:

- A rebuttal letter that responds to each point raised by the academic editor and reviewer(s). You should upload this letter as a separate file labeled 'Response to Reviewers'.
- A marked-up copy of your manuscript that highlights changes made to the original version. You should upload this as a separate file labeled 'Revised Manuscript with Track Changes'.
- An unmarked version of your revised paper without tracked changes. You should upload this as a separate file labeled 'Manuscript'.

If you would like to make changes to your financial disclosure, please include your updated statement in your cover letter. Guidelines for resubmitting your figure files are available below the reviewer comments at the end of this letter.

If applicable, we recommend that you deposit your laboratory protocols in protocols.io to enhance the reproducibility of your results. Protocols.io assigns your protocol its own identifier (DOI) so that it can be cited independently in the future. For instructions see: [https://journals.plos.org/plosone/s/submission-guidelines#loc-laboratory-protocols](https://track.editorialmanager.com/CL0/https:%2F%2Fjournals.plos.org%2Fplosone%2Fs%2Fsubmission-guidelines%23loc-laboratory-protocols/1/010f019440a44836-3797b4f5-3179-4b8a-a2b1-8bd86878400f-000000/I3nVp_7yDZ48iiEQ5DtII1phCZ8iFuCKJwwQ0VSJSPU=192). Additionally, PLOS ONE offers an option for publishing peer-reviewed Lab Protocol articles, which describe protocols hosted on protocols.io. Read more information on sharing protocols at [https://plos.org/protocols?utm_medium=editorial-email&utm_source=authorletters&utm_campaign=protocols](https://track.editorialmanager.com/CL0/https:%2F%2Fplos.org%2Fprotocols%3Futm_medium=editorial-email%26utm_source=authorletters%26utm_campaign=protocols/1/010f019440a44836-3797b4f5-3179-4b8a-a2b1-8bd86878400f-000000/0OCvuYN_m7JgvpswxrvVwyMMmtBi3RzVHl3Tu-F6uHA=192).

We look forward to receiving your revised manuscript.

Kind regards,

Alison Parker
Academic Editor
PLOS ONE

Journal Requirements:

When submitting your revision, we need you to address these additional requirements.

1. Please ensure that your manuscript meets PLOS ONE's style requirements, including those for file naming. The PLOS ONE style templates can be found at

https://journals.plos.org/plosone/s/file?id=wjVg/PLOSOne_formatting_sample_main_body.pdf and

[https://journals.plos.org/plosone/s/file?id=ba62/PLOSOne_formatting_sample_title_authors_affiliations.pdf](https://track.editorialmanager.com/CL0/https:%2F%2Fjournals.plos.org%2Fplosone%2Fs%2Ffile%3Fid=ba62%2FPLOSOne_formatting_sample_title_authors_affiliations.pdf/1/010f019440a44836-3797b4f5-3179-4b8a-a2b1-8bd86878400f-000000/8byx60REEGhUAfzobInxPV24tdggycT8QqsWwGenggQ=192)

2. Please include your full ethics statement in the ‘Methods’ section of your manuscript file. In your statement, please include the full name of the IRB or ethics committee who approved or waived your study, as well as whether or not you obtained informed written or verbal consent. If consent was waived for your study, please include this information in your statement as well.

3. Please ensure that you refer to Figure 8 in your text as, if accepted, production will need this reference to link the reader to the figure.

4. Please review your reference list to ensure that it is complete and correct. If you have cited papers that have been retracted, please include the rationale for doing so in the manuscript text, or remove these references and replace them with relevant current references. Any changes to the reference list should be mentioned in the rebuttal letter that accompanies your revised manuscript. If you need to cite a retracted article, indicate the article’s retracted status in the References list and also include a citation and full reference for the retraction notice.

Response: Full ethics statement is presented under methods, sub-section 2.4 ethical issues. The ethical letter was shared, together with the manuscript and it can be shared again if necessary. In addition, guiding templates for the title and the body have been used to design the manuscript; references reviewed for errors. This research was non-clinical and therefore, laboratory protocol is not applicable for this study. Fig 8 reviewed. Figures used in the manuscript are changed to tif file using PACE and uploaded separately from the manuscript. However, the manuscript maintains figure numbers and captions on separate pages where figures should be placed.

[Note: HTML markup is below. Please do not edit.]

Reviewers' comments:

Reviewer's Responses to Questions

**Comments to the Author**

1. Is the manuscript technically sound, and do the data support the conclusions?

The manuscript must describe a technically sound piece of scientific research with data that supports the conclusions. Experiments must have been conducted rigorously, with appropriate controls, replication, and sample sizes. The conclusions must be drawn appropriately based on the data presented.

Reviewer #1: Partly

Reviewer #2: Yes

Reviewer #3: Yes

2. Has the statistical analysis been performed appropriately and rigorously?

Reviewer #1: Yes

Reviewer #2: Yes

Reviewer #3: No

3. Have the authors made all data underlying the findings in their manuscript fully available?

The [PLOS Data policy](http://track.editorialmanager.com/CL0/http:%2F%2Fwww.plosone.org%2Fstatic%2Fpolicies.action%23sharing/1/010f019440a44836-3797b4f5-3179-4b8a-a2b1-8bd86878400f-000000/BoDABu2fHJaL4PB-EFbzG32naLGiedAiHk83sxmd1mM=192) requires authors to make all data underlying the findings described in their manuscript fully available without restriction, with rare exception (please refer to the Data Availability Statement in the manuscript PDF file). The data should be provided as part of the manuscript or its supporting information, or deposited to a public repository. For example, in addition to summary statistics, the data points behind means, medians and variance measures should be available. If there are restrictions on publicly sharing data—e.g. participant privacy or use of data from a third party—those must be specified.

Reviewer #1: Yes

Reviewer #2: Yes

Reviewer #3: Yes

4. Is the manuscript presented in an intelligible fashion and written in standard English?

PLOS ONE does not copyedit accepted manuscripts, so the language in submitted articles must be clear, correct, and unambiguous. Any typographical or grammatical errors should be corrected at revision, so please note any specific errors here.

Reviewer #1: Yes

Reviewer #2: Yes

Reviewer #3: Yes

5. Review Comments to the Author

Please use the space provided to explain your answers to the questions above. You may also include additional comments for the author, including concerns about dual publication, research ethics, or publication ethics. (Please upload your review as an attachment if it exceeds 20,000 characters)

Reviewer #1: The author needs to have all the grammatical errors corrected before submitting the paper for review. Some paragraphs are too wordy, this needs to be addressed without interfering with the meaning. There is poor flow of sentences as well, Some parafraphs do not trelate to the previous ones

Response: The authors made extensive editing to improve word economy, clarity ,grammar error and coherence for the full manuscript.

Reviewer #2: I appreciate the work addressing this timely public health issue. I would like to raise few concerns regarding the study:
1. Method section
You mentioned that you utilized both methods. Could you clarify the purpose of the cross-sectional study? Additionally, what type of experimental design did you implement (e.g., true randomization, quasi-experiment, longitudinal, etc.)?

Response: The school MHM and WASH intervention began in July 2018; however, the study on the school intervention started in April 2023. Therefore, it was not possible to conduct a baseline survey, as time had already passed by the time the study began. Therefore, it was not possible to contrast baseline data with school interventions’ outcomes. Instead, the study used control schools that did not have school interventions to compare against the schools that received the WASH interventions. Consequently, longitudinal study design was not feasible due to the absence of baseline data and therefore, the study used cross-sectional design to measure the commutative effect of the school intervention by comparing with control schools. The study used quasi experimental design, details indicated under the following section.

For the detail see under methods, section, 2.2 Study design

Sampling method: The study district, Last District Education Office, identified 35 schools that had severe WASH service shortages before the beginning of the school intervention. Therefore, any non-governmental organization that intended to support schools could select any one of these schools, depending on its budget and work force capacity. Plan International Ethiopia, an international non-governmental organization, randomly selected 10 schools in 2018 for school MHM and WASH interventions. Therefore, the study randomly selected three sample schools among 10 WASH intervention schools to evaluate changes caused by the school intervention.

Similarly, for the control schools, from 25 schools that did not have school WASH interventions, the study selected randomly three sample schools.

Details indicated under Sampling method; section 2.5

2. Details of the Intervention
The description of the intervention lacks specificity in terms of quantity, frequency, and duration. Additionally, the content of the intervention is described too superficially, making it difficult to derive meaningful insights or formulate policy recommendations based on the findings.

Response: The school intervention incorporated a comprehensive approach to menstrual hygiene management (MHM), including MHM education, access to sanitary products (with training on how to prepare these products), provision of clean water, sex-segregated latrines, and a dedicated MHM room. The MHM room served as a space where girls could change sanitary pads and engage in peer-to-peer education. Soap was also provided to promote proper hygiene practices. The MHM education included both girls and boy students to get the support of boys.

Quantity: For each WASH intervention school, the program provided integrated MHM and WASH services. These services included access to sex-segregated latrines, tailored to the existing facilities at each school. For example, if a school already had one improved latrine, the program built one additional latrine exclusively for girls. However, if a school had no latrines, the intervention constructed two—one for girls and one for boys.

The program also provided MHM education, one MHM room per school, and sanitary products sufficient to last six months. These products included clean fabric cloths for sanitary product preparation, with the quantity determined by the number of students in each school. Additionally, access to water was ensured through piped systems, from either nearby community water sources or directly within the schools.

MHM education continued in each school until all students had participated, primarily through peer-to-peer education and training sessions.

Frequency: School interventions are limited by budget and implementation timelines. Once schools receive the necessary support and training, they are expected to sustain these services in collaboration with the district education office. However, to ensure a smooth transition and adequate experience, the intervention continued its support until January 2024.

The frequency of support varied based on the nature of the services. Some services, such as latrines, are designed for long-term use, while others, like sanitary pads, have shorter lifespans. For instance, the intervention provided sanitary pad support twice. However, to address shortages, girls were trained to prepare sanitary pads themselves using fabric cloths supplied by the program and other accessible local materials.

Regarding MHM education, once all students were reached through peer-to-peer training, MHM club members took responsibility for advising pubertal girls and providing education to newcomers, especially at the start of the academic year. Similarly, soap was provided twice by the intervention, but menstruating girls contributed a small monthly amount to purchase soap during shortages.

For water access, maintenance was often required. Schools managed these maintenance activities in collaboration with the district education office. However, as the schools and district education office had budget shortage, the school intervention/program was managing all maintenance works until the end of the intervention.

Moreover, to ensure the proper functioning of the school intervention and equip intervention schools with the necessary skills, school program facilitators conducted consistent follow-ups from July 2018 to January 2024, providing both technical and financial assistance.

Duration: The school intervention spanned over six years, from July 2018 to January 2024. However, its activities were primarily conducted during school terms and were interrupted at times due to the COVID-19 pandemic and months lost to conflict in the district.

Content of intervention: There were distinct differences between intervention and control schools. While intervention schools had access to the services outlined under "quantity" above, control schools lacked all of these services due to the absence of similar interventions. The only support available to control schools was general awareness on MHM, provided by the district's "Women and Children Affairs Office" in line with the national education policy, along with non-sex-segregated latrines. Control schools did not have access to water, soap, sanitary products, peer-to-peer education and dedicated MHM rooms.

Included under methods, section 2.1 Description of school interventions

3. Contamination
The manuscript does not clearly outline the strategies implemented to prevent contamination or to buffer against potential biases.

Response: The intervention and control schools are located in rural areas where communities are sparsely populated, and schools are situated far apart. Additionally, due to gender-based violence and security concerns, girls in the rural areas do not travel long distances to attend school. As a result, only girls living near schools have the opportunity to pursue their education.

This geographical isolation meant there was no opportunity for MHM knowledge to be transferred from one school to another, as the distance between schools was significant. Furthermore, there was no deliberate knowledge transfer from intervention schools to control schools. To ensure the validity of the study, the authors verified the absence of other WASH interventions in the control schools or nearby areas before including them in the study.

Therefore, to address the issue of contamination, the study-employed strategies including conducting the research among rural schools, verifying the absence of WASH interventions in the control schools, and ensuring that no deliberate attempts were made to transfer knowledge from intervention to control schools.

Included under methods, section, 2.1 Description of school intervention-last part

4. Ethical Considerations
It is important to understand how the researchers addressed ethical dilemmas faced by non-intervention schools. Clarification on this matter is necessary.
Students under the age of 18 are included in your study. In this case, you must obtain both assent from the students and consent from their parents. Why was not assent considered?

Response: **Ethical Dilemma:** The control schools were not provided with MHM education or WASH services—not because of the research or for the purposes of the study, but due to budget constraints faced by the district education office in providing hygiene facilities and WASH services to all schools. In addition, the school intervention targeted only 10 schools in the district, based on its financial and work force capacity, while there were more schools needing support. The study found that 70% of schools in the district lacked access to water, and 67% did not have improved latrines. The control schools were among those still in need of WASH services, highlighting the broader challenge of resource limitations in the district.

**Students’ assent/consent**: As described in the methods section, students provided both verbal and written consent to participate in the study. Initially, the girls were contacted prior to the day of completing the questionnaire, and their verbal consent was obtained. The following day, after having sufficient time to discuss the matter with their parents and among themselves, those who volunteered to participate provided written assent. Each student signed the designated space on the questionnaire guide before beginning the response process. This ensured that participation was voluntary and informed, with both verbal and written consent documented

**Guardian consent:** Before data collection, the study schools, in collaboration with the Parents and Teachers’ Association (PTA)—a body that fosters communication between schools and communities on educational matters—invited the parents of menstruating girls to attend a brief morning meeting. During the meeting, the school principal and PTA representatives provided an overview of the existing school hygiene facilities and services.

Parents were then asked for their permission to allow their daughters to share their views on the status of the school hygiene services. Therefore, verbal consent was obtained from parents, while those who participated in focus group discussions (FGDs), they also provided written consent by signing a consent form.

Included under methods, section: 2.4 ethical issues

5. Quality Control Mechanisms
What quality control mechanisms were in place to ensure the fidelity of the intervention, particularly regarding educational components? Blinding, randomization, and other quality control mechanisms need to be clearly articulated.

Response: Quality control: Randomization and blinding: Stratified sampling was used to group 10 intervention and 25 non-control schools based on their similarity in WASH experience. This was followed by selecting three schools randomly from each group. For the intervention schools, the 10 schools were given codes and the codes were mixed. From the mixed school codes, a person who was not part of the process picked three school codes randomly. This procedure was repeated for 25 control schools.

Similarly, to select menstruating girl students, a lottery method was used but it followed multistage sampling: First, all boy students were excluded from the sample, as the focus of the study was on girl students. Second, all non-menstruating girl students were excluded from the sample because the study aimed at evaluating the experience of menstruating girl students.

After the exclusion of boys and non-menstruating girls, the study team identified 333 and 293 menstruating girl students from intervention and control schools respectively. This made 626-study population. Using, the sample size formula and after inclusion of the probably of non-attendant rate, the sample size was 360. Thus, for the sample size, a separate list of girls was prepared for each of the six schools. In the study population, the contribution of the intervention schools was 53% while the control schools was 46.6%. Based on this contribution, 192 and 168 menstruating girls were allocated to intervention and control schools orderly.

Each school received its sample size/share based on its contribution to the study population. Using the prepared list of menstruating girls, a lottery method was used to identify samples from each school. For a school that had 50 menstruating girl students in the list but its sample share/size 25, the study team prepared 50 draws and mixed them. Among 50 draws/pieces of paper, 25 of them were given codes/numbers from 1-25, while the remaining were zero. Then, based on the order of the list of students, each student was asked to withdraw a code. If the draw reads one of the numbers from 1-25, a girl is part of the final sample but if the draw reads zero, a girl is excluded from the sample. Therefore, in both the school and girl student selection, the study used simple random techniques.

Blinding one of the quality controlling techniques used to avoid biases during data collection, data analysis, reporting, etc. from those involved in the research process. Though double blinding was not possible, single blinding was used. Participating girl students were blinded due to long years of program implementation in the school where they had been actively engaged from the start of the school intervention and they had different progress evaluation meetings. Thus, they took their participation in the study process as part of the program implementation not as a research that was different from the program implementation. For data collectors, the researcher provided data collectors’’ training where data collectors bias ’was one of the major components of the training.

In addition, to minimize the researcher’s bias, the study employed different approaches. It employed mixed method of data collection to improve the accuracy of the research finding; used clinical data (recorded from MHM related treatment) to cross check against field data from girl students; used independent samples T-test to avoid considering minor differences between intervention and control school as significant. Furthermore, when the literature reviews are observed, both negative and positive findings were equally presented; it tried to avoid confirmation bias, by employing socio-ecological perspectives that helped the study to consider different levels of factor analysis (individual, group, community, organization, and policy) , which were not common in the previous study designs.

Furthermore, the study used random sampling to determine the sample size and the sample represented 57% of the study population in the six study schools.

Included under methods, section, 2.5, sampling methods

6. Mixed Methods Approach
The implementation of mixed methods is noted. Please clarify the sequence of the qualitative and quantitative studies: which method was conducted first, and what was the rationale behind this choice?

Response: Yes, the study used both qualitative and quantitative methods to improve the strength of the evidence. In terms of sequence, qualitative method was used first to understand the views of the target groups and make any necessary change on the quantitateive method. However, there were also groups and individuals targeted by the qualitative method alone, like, office heads and girls’ mothers. In the second case, only qualitative methods was used and therefore, no sequence. School facility observation was another example.

Clarified under methods, section 2.3

*Institutional Written assent*:- Additionally, the study obtained written consent from Lasta District Education Office that was responsible for monitoring, coordinating, and managing all schools in the district. **Under methods**, sub section 2.4

7. Controlling for Confounding Factor
Given that school performance can be influenced by multiple factors, it is essential to explain how the study controlled for the effects of other confounding variables on both school attendance and educational performance.

Response:

**Confounding Variables and Mitigation Strategies used**

Confounding variables are factors, other than the school interventions, that can influence both independent and dependent variables. To minimize the effect of confounding factors on girls’ class attendance and performance, the study employed the following strategies:

1. **Restriction:**
   The study restricted the population to girls of similar age and educational level to reduce variability caused by potential confounding variables. The majority of the sampled students were between 13 and 15 years old, all female, and at comparable educational level— students in the Primary 2nd Cycle Schools (Grades 7 and 8). These restrictions ensured that participants shared similar values for key confounding factors.
2. **Matching:**
   To control for potential confounding variables, girls in the intervention schools were matched with girls in the control schools who had similar characteristics. These included physiological factors, level of education, family educational background and income, age, exposure to technology, and school settings (both schools were located in rural areas). By ensuring that confounding factors were consistent between the two groups, any differences identified in the study could more confidently be attributed to the school interventions.
3. **Transportation and Academic Performance:**
   Since both intervention and control schools were in rural settings, students shared similar experiences regarding school travel. Almost all girls walked to school, with no notable differences in access to transportation. Additionally, the study accounted for prior academic performance by using mid-year educational performance data from 2024 for comparison, thereby mitigating the influence of pre-existing academic disparities.
4. **Teaching Quality and Learning Environment:**
   Although individual teacher differences could exist, significant differences in teaching quality were unlikely, as neither intervention nor control schools had access to advanced educational technologies or resources. Both were located in similar rural environments, ensuring comparable learning conditions.
5. **Cultural and Religious Homogeneity:**
   The majority of the girls from both intervention and control schools shared the same ethnic background (98% Amhara) and religious faith (84% Orthodox), which reduced the potential influence of cultural or religious differences on the study outcomes.

**Presented under Methods section, 2.6**

I recommend that all these concerns be addressed in the body of the manuscript to enhance its clarity and robustness.

Reviewer #3: Thank you for asking me to review this paper on “Effects of school menstrual hygiene, water, and sanitation interventions on girls’ empowerment, health, and educational outcomes: Lasta District, Amhara Regional State, Ethiopia." It is exciting research. However, the following issues must be improved before it is accepted for publication.

The title of this study, which is stated as "Effects of School Menstrual Hygiene, Water, and Sanitation Interventions on Girls’ Empowerment, Health, and Educational Outcomes,” needs modifications. The intervention section, as indicated in lines 184–188, includes MHM education and MHM rooms, which are not included in this title. Therefore the title of this study should be modified to “Effects of School Menstrual Hygiene Management and Water, Sanitation and Hygiene (WASH) Service Interventions on Girls’ Empowerment, health and Educational Outcomes.”

Response: Improvement has been made to the title and abstract and so does on the rest of the of manuscript. However, the word’ services’ may not be necessary as the reference in the title is the school program/the intervention in order to realize the MHM services . We felt it is also better to use the abbreviation , WASH, in the body, rather than in the title. Therefore, the revised title reads as’’ Effects of School Menstrual Hygiene Management, Water,Sanitation and Hygiene Interventions on Girls’ Empowerment, Health and Educational Outcomes.’’

Abstract sections: lines 31–33 should be consistent with the modified title. Please modify it as “This study investigated the effects of school menstrual hygiene management and water, sanitation, and hygiene (WASH) service interventions on girls’ empowerment, health, and educational outcomes. And also use effect or impacts consistently throughout the documents.

Response: Similar change has been made to the abstract

The introduction sections: The authors well explained the context of the study. However, lines 157-160 need rearrangements. Line 157, “…..The significance of this study...” should come after line 160.
Design and setting: in line 173, the authors stated, “Six primary second-cycle schools were selected within the district” without describing first how many primary second-cycle schools are there in the district. The authors should also describe how six primary second-cycle schools were selected. Is randomization conducted? Or is there a baseline difference between exp’tal and controlled schools? Otherwise, it is difficult to conclude whether the effects after six years were due to interventions or results of their baseline difference.

Response: Rearrangement on the indicated lines have been made; clarifications included on how the 6 study schools were selected and the methods used to select schools:- indicated under methods sections. 2.5. Sampling methods and sample size. However, due to changes made following reviewers’ comments, line numbers referred above many have changed. So, it may require going through many paragraphs to find changes.

Line 180: “10 primary second cycle schools or 6? This contradicts what was stated in line 173.
The informed consent section from line 234-262 needs modification. The informed voluntary consent for a minor (age < 18 years)/vulnerable individual should be signed by his/her legal competent representative (e.g., a parent/guardian). But in this study, even though around 85% of study participants were less than 16 years old (as indicated in table 1), informed voluntary consent taken from parent/guardian was not explained.

Response: The 10 primary 2^nd^ Cycle schools were the intervention schools only; however, the six schools are the study schools, three from the 10 intervention schools and another three from control schools. To avoid confusion, additional clarifications are given under method, sub-section 2.1-description of school interventions and 2.5-Sampling method. Regarding consent, detail explanations are added under methods, sub-section.2.4. Ethical issues.

Results section: Please rewrite from lines 284 to 288. For example, “Parental education levels were generally low...” Do not interpret the findings in the result section; keep it for the discussion section.
Please remove the gender variable from table 1.
The conclusion section is not well written. The authors should stick to the findings of the study during the conclusion. For instance, line 764-765, ”The interventions significantly boosted their confidence in discussing menstruation and lowered anxieties,” should be removed as not a tool that measures anxiety was indicated in the result.
Please rewrite the structured recommendation for this study and remove lines 770–778.
Finally, there are numerous editorial and grammatical errors that should be corrected before this manuscript is accepted. For instance, the author should use MHM consistently throughout the documents once he/she defined menstrual hygiene management as (MHM), unless it comes after full stop or at the beginning of the paragraph.

Response: Manuscript conclusion and recommendations revised and improved, Section: 6

6. PLOS authors have the option to publish the peer review history of their article ([what does this mean?](https://track.editorialmanager.com/CL0/https:%2F%2Fjournals.plos.org%2Fplosone%2Fs%2Feditorial-and-peer-review-process%23loc-peer-review-history/1/010f019440a44836-3797b4f5-3179-4b8a-a2b1-8bd86878400f-000000/1s2zrtzQK6g5U-_WO1lH7m7XIrGXtNmxjMO5wMXQp_Y=192)). If published, this will include your full peer review and any attached files.


If you choose “no”, your identity will remain anonymous but your review may still be made public.


**Do you want your identity to be public for this peer review?** For information about this choice, including consent withdrawal, please see our [Privacy Policy](https://track.editorialmanager.com/CL0/https:%2F%2Fwww.plos.org%2Fprivacy-policy/1/010f019440a44836-3797b4f5-3179-4b8a-a2b1-8bd86878400f-000000/k4VfN9FV_aCaoSYIRw-wDN20irtOFxJgzxfsa0Tkf1U=192).

Reviewer #1: No

Reviewer #2: **Yes:** Balem Demtsu Betsu

Reviewer #3: **Yes:** Abera Cheru

[NOTE: If reviewer comments were submitted as an attachment file, they will be attached to this email and accessible via the submission site. Please log into your account, locate the manuscript record, and check for the action link "View Attachments". If this link does not appear, there are no attachment files.]

While revising your submission, please upload your figure files to the Preflight Analysis and Conversion Engine (PACE) digital diagnostic tool, [https://pacev2.apexcovantage.com/](https://track.editorialmanager.com/CL0/https:%2F%2Fpacev2.apexcovantage.com%2F/1/010f019440a44836-3797b4f5-3179-4b8a-a2b1-8bd86878400f-000000/S1lVuzISRVUN2TsR_CsU42P8aih60LBfuXnqJ8uRr4w=192). PACE helps ensure that figures meet PLOS requirements. To use PACE, you must first register as a user. Registration is free. Then, login and navigate to the UPLOAD tab, where you will find detailed instructions on how to use the tool. If you encounter any issues or have any questions when using PACE, please email PLOS at [figures@plos.org](mailto:figures@plos.org). Please note that Supporting Information files do not need this step.
