# Supplementary material for: Effects of school menstrual hygiene management, water, sanitation, and hygiene interventions on girls’ empowerment, health, and educational outcomes: Lasta district, Amhara regional state, Ethiopia
Source: PLoS One. 2025 Apr 28;20(4):e0321376. doi: 10.1371/journal.pone.0321376 (PMC12036919; doi:10.1371/journal.pone.0321376)
Supplement: Attachment — Submitted filename: PONE-D-24-55215_R1 (1).docx [file pone.0321376.s007.docx]

**PLOS ONE**

### Effects of school menstrual hygiene management, water, sanitation and hygiene interventions on girls’ empowerment, health and educational outcomes: Lasta

**District, Amhara Regional State, Ethiopia**

--Manuscript Draft--

| **Manuscript Number:** | PONE-D-24-55215R1 |
| --- | --- |
| **Article Type:** | Research Article |
| **Full Title:** | Effects of school menstrual hygiene management, water, sanitation and  hygiene interventions on girls’ empowerment, health and educational outcomes: Lasta District, Amhara Regional State, Ethiopia |
| **Short Title:** | Effects of school based menstrual hygiene management interventions on girls' empowerment |
| **Corresponding Author:** | Fisseha Atale Andargie, M.As Plan International  - please select -, GUYANA |
| **Keywords:** | Girls' empowerment, physical, emotional and social health, class attendance, academic performance, menstrual hygiene management education, |
| **Abstract:** | This study examined the effects of school-based menstrual hygiene management (MHM) and water, sanitation, and hygiene (WASH) interventions on menstruating girls' empowerment, health, and educational outcomes using cross-sectional and experimental designs. It explored whether access to MHM education, water, sanitation, and hygiene (WASH) facilities and services could significantly influence girls' self- confidence, physical, emotional, and social health, class attendance, and academic performance. The findings demonstrated a marked improvement in girls’ empowerment in intervention schools, with 54% feeling confident purchasing sanitary products compared to 18% in control schools, indicating enhanced emotional well-being.  Similarly, 21% and 22% of girls in intervention schools felt comfortable discussing MHM with boys and mothers, respectively, compared to only 9% in control schools, reflecting improved social health. Additionally, 51% of menstruating girls in intervention schools practised genital hygiene three to four times per day, compared to 33% in control schools, signifying better physical health. Regarding educational outcomes, 68% of girls in intervention schools attended class during menstruation, compared to 30% in control schools, and 78% of girls in intervention schools reported having adequate study time at home, compared to 41% in control schools. Despite these notable improvements, no significant difference in academic performance was observed between the two groups of girls. Overall, the findings suggest that school- based MHM and WASH interventions can substantially empower menstruating girls, improve their physical, emotional, and social health, and reduce menstrual-related absenteeism.  Keywords: Girls’ empowerment, physical, emotional and social health, class attendance, academic performance, hygiene education, |
| **Order of Authors:** | Fisseha Atale Andargie, MA |
|  | Femi Rufus TINUOLA, Ph.D |
| **Response to Reviewers:** | Response to the Editor: Full ethics statement is presented under methods, sub-section  2.4 ethical issues. The ethical letter was shared, together with the manuscript and it can be shared again if necessary. In addition, guiding templates for the title and the body have been used to design the manuscript; references reviewed for errors. This research was non-clinical and therefore, laboratory protocol is not applicable for this study. Fig 8 reviewed. Figures used in the manuscript are changed to tif file using PACE and uploaded separately from the manuscript. However, the manuscript maintains figure numbers and captions on separate pages where figures should be placed. Other details are indicated on the ' response to reviewers section.  2. Response to reviewer 1: The authors made extensive editing to improve word economy, clarity ,grammar error and coherence for the full manuscript.  Response to reviewer 2: The school MHM and WASH intervention began in July 2018; however, the study on the school intervention started in April 2023. Therefore, it was |

|  | not possible to conduct a baseline survey, as time had already passed by the time the study began. Therefore, it was not possible to contrast baseline data with school interventions’ outcomes. Instead, the study used control schools that did not have school interventions to compare against the schools that received the WASH interventions. Consequently, longitudinal study design was not feasible due to the absence of baseline data and therefore, the study used cross-sectional design to measure the commutative effect of the school intervention by comparing with control schools. The study used quasi experimental design, details indicated under the following section.  For the detail see under methods, section, 2.2 Study design . Detail response for all question to reviewer 2 is indicated on ' response to reviewers' and manuscript.  Response to reviewer3: Improvement has been made to the title and abstract and so does on the rest of the of manuscript. However, the word’ services’ may not be necessary as the reference in the title is the school program/the intervention in order to realize the MHM services . We felt it is also better to use the abbreviation , WASH, in the body, rather than in the title. Therefore, the revised title reads as’’ Effects of School Menstrual Hygiene Management, Water, Sanitation and Hygiene Interventions on Girls’ Empowerment, Health and Educational Outcomes.’’ The detial response to reveiwer 3 is also indicated on ' response to reviewers' and manuscript. |
| --- | --- |
| **Additional Information:** |  |
| **Question** | **Response** |
| **Financial Disclosure**  Enter a financial disclosure statement that describes the sources of funding for the work included in this submission. Review the [submission guidelines](http://journals.plos.org/plosone/s/submission-guidelines#loc-financial-disclosure-statement) for detailed requirements. View published research articles from [*PLOS ONE*](https://journals.plos.org/plosone/) for specific examples.  This statement is required for submission and **will appear in the published article** if the submission is accepted. Please make sure it is accurate. | The author(s) received no specific funding for this work. |
| **Funded studies**  Enter a statement with the following details:   - Initials of the authors who received each award - Grant numbers awarded to each author - The full name of each funder - URL of each funder website - Did the sponsors or funders play any role in the study design, data collection and analysis, decision to publish, or preparation of the manuscript? |  |
| Did you receive funding for this work? |  |
| **Competing Interests** | I have read the Journal's policy and authors of this manuscript have declared that they have no competing interest. |

| Use the instructions below to enter a competing interest statement for this submission. On behalf of all authors, disclose any [competing interests](http://journals.plos.org/plosone/s/competing-interests) that could be perceived to bias this work—acknowledging all financial support and any other relevant financial or non- financial competing interests.  This statement is required for submission and **will appear in the published article** if the submission is accepted. Please make sure it is accurate and that any funding sources listed in your Funding Information later in the submission form are also declared in your Financial Disclosure statement.  View published research articles from  [*PLOS ONE*](http://journals.plos.org/plosone/) for specific examples. |  |
| --- | --- |
| **NO authors have competing interests**  Enter: *The authors have declared that no competing interests exist.*  **Authors with competing interests**  Enter competing interest details beginning with this statement:  *I have read the journal's policy and the authors of this manuscript have the following competing interests: [insert competing interests here]* |  |
| * typeset |  |
| **Ethics Statement**  Enter an ethics statement for this submission. This statement is required if the study involved:   - Human participants - Human specimens or tissue - Vertebrate animals or cephalopods - Vertebrate embryos or tissues - Field research | The study was a field research that participated girl students' during interviews, questionnaire response and focus group discussions; however it did not include human tissues and vertebrate animals. For the participation of girl students in the research process, ethical clearance letter was obtained from district education office which administers all schools in the district. The ethical clearance letter is uploaded in the 'attach files' section. Moreover, a written consent was obtained from students and this is indicated in the methods section of the manuscript. |

| Write "N/A" if the submission does not require an ethics statement.  General guidance is provided below. Consult the [submission guidelines](http://journals.plos.org/plosone/s/submission-guidelines#loc-guidelines-for-specific-study-types) for detailed instructions. **Make sure that all information entered here is included in the Methods section of the manuscript.** |  |
| --- | --- |
| **Format for specific study types**  **Human Subject Research (involving human participants and/or tissue)**   - Give the name of the institutional review board or ethics committee that approved the study - Include the approval number and/or a statement indicating approval of this research - Indicate the form of consent obtained (written/oral) or the reason that consent was not obtained (e.g. the data were analyzed anonymously)   **Animal Research (involving vertebrate animals, embryos or tissues)**   - Provide the name of the Institutional Animal Care and Use Committee (IACUC) or other relevant ethics board that reviewed the study protocol, and indicate whether they approved this research or granted a formal waiver of ethical approval - Include an approval number if one was obtained - If the study involved *non-human primates*, add *additional details* about animal welfare and steps taken to ameliorate suffering - If anesthesia, euthanasia, or any kind of animal sacrifice is part of the study, include briefly which substances and/or methods were applied   **Field Research**  Include the following details if this study involves the collection of plant, animal, or other materials from a natural setting:   - Field permit number - Name of the institution or relevant body that granted permission |  |
|  |  |

| **Data Availability**  Authors are required to make all data underlying the findings described fully available, without restriction, and from the time of publication. PLOS allows rare exceptions to address legal and ethical concerns. See the [PLOS Data Policy](http://journals.plos.org/plosone/s/data-availability) and [FAQ](http://journals.plos.org/plosone/s/data-availability#loc-faqs-for-data-policy) for detailed information.  A Data Availability Statement describing where the data can be found is required at submission. Your answers to this question constitute the Data Availability Statement and **will be published in the article**, if accepted. | Yes - all data are fully available without restriction |
| --- | --- |
| **Important:** Stating ‘data available on request from the author’ is not sufficient. If your data are only available upon request, select ‘No’ for the first question and explain your exceptional situation in the text box. |  |
| Do the authors confirm that all data underlying the findings described in their manuscript are fully available without restriction? |  |
| **Describe where the data may be found in full sentences. If you are copying our sample text, replace any instances of XXX with the appropriate details.**   - If the data are **held or will be held in a public repository**, include URLs, accession numbers or DOIs. If this information will only be available after acceptance, indicate this by ticking the box below. For example: *All XXX files are available from the XXX database (accession number(s) XXX, XXX.*). - If the data are all contained **within the manuscript and/or Supporting Information files**, enter the following: *All relevant data are within the manuscript and its Supporting Information files.* - If neither of these applies but you are able to provide **details of access elsewhere**, with or without limitations, please do so. For example: | All relevant data are within the manuscript and its Supporting Information files. |

| *Data cannot be shared publicly because of [XXX]. Data are available from the XXX Institutional Data Access / Ethics Committee (contact via XXX) for researchers who meet the criteria for access to confidential data.*  *The data underlying the results presented in the study are available from (include the name of the third party and contact information or URL).*   - This text is appropriate if the data are owned by a third party and authors do not have permission to share the data.   * typeset |  |
| --- | --- |
| Additional data availability information: |  |

Manuscript	[Click here to access/download;Manuscript;Manuscript.docx](https://www2.cloud.editorialmanager.com/pone/download.aspx?id=39267850&guid=1f33c615-47dc-4f76-9c13-e8a0a8f91b17&scheme=1)[<https://www2.cloud.editorialmanager.com/pone/download.aspx?id=39267850&guid=1f33c615-47dc-4f76-9c13-e8a0a8f91b17&scheme=1>](https://www2.cloud.editorialmanager.com/pone/download.aspx?id=39267850&guid=1f33c615-47dc-4f76-9c13-e8a0a8f91b17&scheme=1)

1

2

3 **Effects	of	school	menstrual	hygiene	management,	water,**
4 **sanitation and hygiene interventions on girls’ empowerment,**
5 **health	and	educational	outcomes:	Lasta	District,	Amhara**
6 **Regional State, Ethiopia**

7

8

9

### 10 Effects of school menstrual hygiene management interventions on girls’

11 **empowerment**

12

13

##### 14 Fisseha A.Andargie^1^*¶, Femi R.Tinuola2*&*

15 ^1^Department of Public Health Administration, Central University of Nicaragua,
16 Managua, Nicaragua
17 ^2^Department of Sociology, Federal University Gusau, Zamfara State, Nigeria

18

19

20

21 *Corresponding author
22 E-mail: [fissehaatalie@yahoo.com](mailto:fissehaatalie@yahoo.com) (FA);

23

24	^¶^This author contributed to the article

25	*^&^*This author contributed to the article*.*

26

27

28

29

30

# 31 Abstract

32 This study examined the effects of school-based menstrual hygiene management (MHM)
33 and water, sanitation, and hygiene (WASH) interventions on menstruating girls'
34 empowerment, health, and educational outcomes using cross-sectional and experimental
35 designs. It explored whether access to MHM education, water, sanitation, and hygiene
36 (WASH) facilities and services could significantly influence girls' self-confidence,
37 physical, emotional, and social health, class attendance, and academic performance. The
38 findings demonstrated a marked improvement in girls’ empowerment in intervention
39 schools, with 54% feeling confident purchasing sanitary products compared to 18% in
40 control schools, indicating enhanced emotional well-being. Similarly, 21% and 22% of
41 girls in intervention schools felt comfortable discussing MHM with boys and mothers,
42 respectively, compared to only 9% in control schools, reflecting improved social health.
43 Additionally, 51% of menstruating girls in intervention schools practised genital hygiene
44 three to four times per day, compared to 33% in control schools, signifying better physical
45 health. Regarding educational outcomes, 68% of girls in intervention schools attended class
46 during menstruation, compared to 30% in control schools, and 78% of girls in intervention
47 schools reported having adequate study time at home, compared to 41% in control schools.
48 Despite these notable improvements, no significant difference in academic performance
49 was observed between the two groups of girls. Overall, the findings suggest that school-
50 based MHM and WASH interventions can substantially empower menstruating girls,
51 improve their physical, emotional, and social health, and reduce menstrual-related
52 absenteeism.

53

54 **Keywords:** Girls’ empowerment, physical, emotional and social health, class attendance,
55 academic performance, hygiene education,

56

57

58

59

60

61

62

63

64

## 65 Authors’ summary

66 Improving girls' education has been a key priority in developing countries, as education
67 prepares girls for higher-level positions and decision-making roles. However, traditional
68 attitudes toward education and natural processes such as menstruation continue to pose
69 significant challenges.
70 Studies have examined the effect of menstruation on girls' class attendance and academic
71 performance; however, findings remain inconsistent.Others suggest that menstruation
72 affects attendance, while others report no significant effect. Moreover, many studies
73 focused on the school environment, despite girls being influenced by both school and
74 broader social factors. As a result, solid evidence on the effects of menstruation remains
75 limited.
76 This study is based on a comprehensive set of school interventions to examine the effects
77 of menstruation on girls’ empowerment, health, class attendance and performance. The
78 school interventions included access to sex-segregated toilets, water, sanitary products,
79 designated girls' rooms for resting and changing sanitary pads, and menstrual hygiene
80 management education, while considering external factors such as policy and community
81 influences.
82 Our findings showed that comprehensive MHM education and access to WASH facilities
83 and services significantly reduced class absenteeism among menstruating girls; increased
84 girls' confidence in discussing menstruation with peers, family members, and teachers.
85 Moreover, the school interventions improved girls’ physical, social, and emotional health.

86

87

88

89

90

91

92

93

94

95

96

# 97 1.Introduction

98 Menstruation is a natural physiological process involving the monthly shedding of the
99 uterine lining, occurring among pubescent girls and pre-menopausal women who are not
100 pregnant [1, 2]. Menstrual hygiene management (MHM) refers to the hygienic handling of
101 menstrual blood using appropriate sanitary products and hygiene practices, including
102 absorbent products, soap, water, and safe disposal of used materials [1, 3].
103 Despite its importance, discussions on MHM was considered a taboo, especially in
104 developing nations [4, 5]. Interest in MHM emerged in the 2000s when private and non-
105 governmental organizations began promoting MHM education to pubescent girls, primarily
106 within school settings [6].
107 Therefore, menstruation and MHM is a relatively young field of study, having gained
108 research attention for the last 25 years. In this brief period, research has primarily focused
109 on examining the relationship between menstruation and girls' class absenteeism [7, 8, 9].
110 The United Nations Children's Fund (UNICEF), Plan International, Save the Children, and
111 Emory University in the Philippines conducted an evaluation and identified three key
112 determinants of MHM within school environments that could empower girls. These
113 determinants include access to water, sanitation, and hygiene (WASH) facilities and
114 services, MHM education and practical guidance on hygiene practice, and access to
115 sanitary products. The evaluation reported that the absence of these determinants could lead
116 to unsafe MHM practices, confusion, and anxiety [10].
117 In this study report, empowerment refers to an individual's or group's increased control over
118 their lives, characterized by self-confidence, access to adequate knowledge, and behaviour
119 change, which are essential for making informed decisions regarding health and education
120 [12].
121 Since 2012, UNICEF and the University of Colombia have hosted annual conferences
122 under the theme “WASH in Schools Empowers Girls' Education." Proceedings from these
123 conferences indicate that school WASH interventions have the potential to enhance girls'
124 education and improve MHM practices [11].
125 Moreover, access to school WASH services can help prevent at least 9.1% of the global
126 disease burden [13], and improvements in health outcomes can contribute to higher class
127 attendance and better academic performance [14, 15, 16].
128 Conversely, the absence of school WASH services can negatively affect girls' emotional,
129 social, and physical health, leading to confusion and anxiety due to fear of menstrual
130 leakage, social isolation, and genital bruising from unsafe MHM practices [10]. The World
131 Health Organization defines health as “a state of complete physical, mental, and social well-
132 being, and not merely the absence of disease” [17].
133 Despite evidence of positive associations between school WASH services, girls’ MHM
134 practices, and class attendance, some studies have reported no significant correlation
135 between MHM practices and class attendance.
136 Studies conducted in Nepal and Malawi, for instance, reported that the effect of
137 menstruation on class attendance was minimal**.** Instead, they attributed class absenteeism
138 to household responsibilities and lack of motivation [18, 19]. These findings contrast with
139 studies from northern Ethiopia and Kenya**,** which observed improved class attendance
140 among girls following school WASH interventions [8, 20].
141 However, there is broad agreement on the correlation between class attendance and
142 academic performance.
143 A study found that consistent class attendance, along with regular assessments and study
144 time, was positively correlated with academic performance [21]. In addition, a study on
145 higher education reported that regular attendance reduces the likelihood of poor academic
146 performance [22]. However, while class attendance benefits lower-performing students,
147 this relationship does not extend to higher-performing students [23].
148 Generally, while studies on the association between MHM and class attendance have laid
149 the groundwork for further research, they lack comprehensiveness.
150 First, evaluation reports from Philippine-based non-governmental organizations, the
151 UNICEF, and the University of Colombia were qualitative; they were not supported with
152 quantitative findings [10, 11]. Additionally, no studies, aside from qualitative project
153 completion reports, have explored the empowering potential of school WASH
154 interventions.
155 Second, the findings across studies lack consistency. For instance, research in Nepal and
156 Malawi reported minimal effects of MHM on class attendance. However, studies in
157 northern Ethiopia and Kenya found improved attendance following school WASH
158 interventions, highlighting contradictions in the literature [8, 18, 19, 20].
159 Third, existing research has been confined to in-school factors, overlooking external
160 influences such as educational policies, community dynamics, and peer influence on girls’
161 MHM practices [7, 8, 9, 17]. However, in school and out of school environments play
162 critical roles in shaping these practices [25].
163 Finally, studies on the relations between girls’ MHM practice and health have primarily
164 focused on clinical aspects, such as reproductive tract infections, while neglecting key
165 social, emotional, and physical health dimensions [24].
166 Therefore, the significance of this study stems from the limitations identified in previous
167 studies on MHM and class attendance. Accordingly, this study aimed to examine the effects
168 of school-based menstrual hygiene management, water, sanitation and	hygiene
169 interventions on girls’ empowerment, health, class attendance, and academic performance.

170

171

172

# 173 2. Materials and methods

## 174 2.1 Study setting and description of school interventions

175 The study was conducted in Lasta District, North Wollo Zone, Amhara Regional State,
176 located in northern and north-west part of Ethiopia.
177 Lasta District Education Office identified 35 schools with critical WASH service shortages
178 in 2017. Plan International Ethiopia, an international NGO, randomly selected 10 Primary
179 2nd Cycle schools from 35 schools for MHM and WASH interventions.
180 **Contents of the interventions**: The key components of the program included MHM
181 education, the provision of sanitary products and soap, and access to water and gender-
182 segregated toilets. Additionally, the intervention established MHM rooms, where girls
183 could rest when feeling unwell, change sanitary pads during menstruation, prepare sanitary
184 products, and participate in peer-to-peer MHM education.
185 In contrast, control schools lacked the facilities and hygiene services provided through the
186 MHM and WASH interventions. Their only support came from the District Women and
187 Children Affairs Office, which offered only MHM general awareness as part of the
188 education policy.
189 While intervention schools received MHM education and WASH services, most other
190 schools in the district, including control schools did not due to budget constraints and
191 absence of other WASH actors providing school WASH services. Therefore, lack of access
192 to WASH services in control schools was not a result of the study design but rather a
193 consequence of district budget limitations and the absence of external WASH support.
194 The frequency of MHM education and WASH service support for menstruating girls varied
195 depending on the type of service. Long-term facilities, such as improved latrines and MHM
196 rooms were constructed for sustained use, while consumable items like sanitary pads and
197 soap required regular replenishment.
198 The MHM education continued until all students in the intervention schools, including boys,
199 received training. Afterwards school MHM club members held monthly meetings to
200 monitor MHM practices, support pubescent students, and provide guidance. Additionally,
201 the MHM club organized training sessions for new students at the beginning of each
202 academic year.
203 A primary goal of the school interventions was to equip menstruating girls, schoolteachers,
204 and the district education office with the skills needed to sustain the interventions'
205 achievements using their resources, gradually reducing and eliminating reliance on external
206 support.
207 The school intervention spanned six years, from July 2018 to January 2024. However,
208 activities were primarily conducted during school terms and were temporarily interrupted
209 during school breaks, the COVID-19 pandemic, and district conflicts.
210 Throughout the intervention period, program facilitators from Plan International Ethiopia,
211 in collaboration with the District Women and Children Affairs Office and schoolteachers,
212 conducted regular follow-ups to ensure that female students acquired adequate MHM skills
213 and to address service gaps as they arose.
214 To minimize the risk of contamination in control schools, the study used geographical
215 isolation as its primary strategy. Both intervention and control schools were located in rural,
216 mountainous areas where communities were sparsely populated and schools were far apart.
217 Furthermore, due to concerns about gender-based violence and security, girls in rural
218 communities typically did not travel long distances to attend school. As a result, only
219 menstruating girls living near schools had the opportunity to continue their education. This
220 geographical isolation effectively prevented the transfer of MHM knowledge from
221 intervention schools to control schools.
222 Additionally, before selecting the control schools, the research team verified whether any
223 knowledge transfer had occurred between intervention and control schools or if other
224 WASH interventions were present in or near control schools.
225 Therefore, to address contamination concerns, the research team conducted the study in
226 rural schools, confirmed the absence of similar interventions in control schools, and
227 ensured that no deliberate attempt was made to transfer knowledge from intervention
228 schools to control schools.

## 229 2.2 Study design

230 The study employed a cross-sectional design because the school interventions began in July
231 2018, while this study commenced in April 2023. This timing made a longitudinal design
232 infeasible as it made impossible to compare baseline data and intervention outcomes.
233 Instead, the study compared intervention schools with control schools that had not been
234 exposed to similar interventions.
235 Although the study used random sampling to select both schools and participants, neither
236 the schools nor the girls could switch between groups. Girls from intervention schools had
237 no opportunity to be part of the control group, and the same applied to girls from control
238 schools. Therefore, the study followed a quasi-experimental design.

### 239 2.3 Methods and data collection instruments

240 The study employed qualitative and quantitative methods to address potential gaps
241 associated with using a single approach. In terms of sequence, the qualitative method was
242 applied first in order to adjust the quantitative method as needed. However, some focus
243 group discussions and key informant interviews were conducted solely for qualitative data
244 collection.
245 To collect qualitative data the study utilized focus group discussions, key informant
246 interviews, and observations. For quantitative data collection, questionnaires, health centre
247 records, and half-year academic performance data for girls were used.

## 248 2.3 Study population

249 The study aimed at assessing the effects of school interventions on menstruating girl
250 students. Therefore, only menstruating girls comprised the study population. However, to
251 evaluate the influence of communities, peers, and policies on girls' MHM practices, male
252 students, mothers of girls, and district office heads were included for qualitative data
253 collection.

254

## 255 2.4 Ethical issues

256 **Participant informed assent***:* A day before participating in the study, the study team
257 briefed the girls on its purpose. The following day, after having sufficient time to discuss
258 their participation with parents and peers, the girls provided written assent by signing the
259 designated space on the questionnaire.
260 **Guardian Informed assent**: Two days before data collection, the study schools,
261 with support from the Parents and Teachers’ Association (PTA)—a body that
262 facilitates collaboration between schools and communities on educational
263 matters, invited girls’ parents to a brief morning meeting. During this session,
264 the study team provided an overview of the study, and following discussions,
265 parents were asked whether they would allow their daughters to participate.
266 As a result, all parents provided verbal consent and those participated at the focus group
267 discussions gave written consent by signing the consent form.
268 ***Institutional Written assent*:** Additionally, the study obtained written consent from Lasta
269 District Education Office that was responsible for monitoring, coordinating, and managing
270 all schools in the district. The full written ethics statement reads as follows:

271

272	Date: 23Feb.2023

273 Lasta District Education Office, North Wollo Zone of Amhara Region, understands the relevance of
274 examining the ‘*’ Effects of School Menstrual Hygiene, Water, Sanitation and hygiene Interventions on*
275 *Girls Hygiene Knowledge and Practice, Empowerment, Health and Educational Outcomes’*’ at the
276 Primary 2nd Cycle Schools found in Lasta District.
277 The Office recognizes that the study will include menstruating girls, boys, teachers and community
278 representatives around the study schools and Heads of WASH Sector Offices in Lasta District. We
279 believe that the results of the study may benefit for Lasta District Education Office in particular and
280 Amhara Regional State in general to improve school WASH interventions.
281 Therefore, Lasta District Education Office has given this ethical clearance letter/permission to conduct
282 the inquiry in the schools and kindly request the study schools and district WASH Sector Offices to
283 support in providing required data to Mr. Fisseha Atale, who is conducting the study.

284

## 285 2.5 Sampling method and sample size

286 The study employed a stratified sampling method to group intervention and non-
287 intervention schools based on their similarity in access to MHM education and WASH
288 services. The research team assigned codes to 10 intervention schools and then mixed
289 these codes. From the mixed set, three school codes were randomly drawn. The same
290 procedure applied to 25 control schools. As a result, three intervention schools were
291 selected from the 10 WASH intervention schools, while three control schools were
292 chosen from 25 control schools in the district.
293 To select girls the study followed a multi-stage sampling approach. First, male students
294 were excluded since the study focused on female students. Second, non-menstruating girls
295 were also excluded, as the study aimed to capture the experiences of menstruating girls.
296 After these exclusions, the research team identified 333 menstruating girls from
297 intervention schools and 293 from control schools, yielding a total study population of 626.
298 Adjusting for the probability of non-attendance and applying a sample size formula, the
299 final sample size was determined to be 360. Within this sample, 192 girls (53%) were
300 allocated to intervention schools, while 168 girls (46.6%) to control schools, maintaining
301 proportional representation.
302 Then the study team prepared separate list of menstruating girls and schools received their
303 sample allocation based on their proportional contribution to the study population. The
304 lottery method was used to allocate samples for each school. For a school that had 50
305 menstruating girls on the list and sample allocation of 25, the research team prepared 50
306 draws, 25 labelled from 1 - 25. The remaining marked as zero. After mixing the codes, each
307 girl, in the order of the list, drew a code. If the draw contained a number between 1 and 25,
308 she was included in the final sample; if it was zero, she was excluded.
309 The research used 95% confidence level and 5% margin of error, with an expected variance
310 of 0.5. Given the known student population, the research applied finite population formula,
311 detailed as follows [26]:-

312	𝑆 = 𝑍^2^𝑁𝑃(1 − 𝑃)/𝑒^2^(𝑁 − 1) + 𝑍^2^𝑃(1 − 𝑃)

313 Where,
314 S: stands for sample size
315 N: Population size of students
316 P: Population proportion, with expected variance of 0.5
317 Z: Z-score, which is 1.96 in this case;
318 e- Margin of error/degree of accuracy, which is 0.05 (5%);
319 For quality control, the study employed single blinding to minimize biases during data
320 collection. Female students from intervention schools had been engaged in school
321 interventions for several years, and their perspectives influenced by their roles in
322 implementing these interventions. Additionally, the confidence gained through these
323 programs gave them prior experience to share their views.
324 For girls in control schools, the study team communicated clearly that the study was not
325 associated with any future opportunities for school WASH interventions. They were
326 informed to share their individual MHM practice and access to WASH services in the
327 school environment.
328 For data collectors the research team provided comprehensive training including why to
329 avoid being biased during data collection.
330 To further control researcher bias and enhance data quality, the study adopted a mixed-
331 method approach for data collection. Clinical data related to MHM were gathered,
332 crosschecked against field data, and analysed using the independent samples t-test to ensure
333 accuracy in the interpretation of differences between menstruating girls. To avoid
334 confirmation bias, the study employed a socio-ecological perspective, allowing for the
335 analysis of multiple levels of factors:- individual, group, community, organizational, and
336 policy.
337 **2.6 Mitigating effects of extraneous variables**
338 Confounding variables are factors other than school interventions that can influence
339 independent and dependent variables. To minimize their impact on the findings, the study
340 employed the following strategies:
341 **Restriction:** The study population was limited to girls of similar age and educational level
342 to reduce variability. Most sampled students (69%) were between 13 and 15 years old and
343 enrolled in Primary Second Cycle Schools (Grades 7 and 8). These restrictions helped
344 establish comparable characteristics for key confounding variables, minimizing their
345 influence on the study outcomes.
346 **Matching:** Girls in intervention schools were matched with those in control schools based
347 on similar characteristics, including family background, income, exposure to technology,
348 and school environment. Both intervention and control schools were located in remote
349 rural areas, where agriculture was the primary source of income for all parents, and access
350 to information was limited due to geographic isolation. By controlling these confounding
351 factors, this method ensured that any differences observed between the two groups could
352 be attributed to the school interventions.
353 **Transportation and Academic Performance:** Since both intervention and control
354 schools were in rural settings, students shared similar experiences regarding school
355 travel—nearly all girls walked to school. Additionally, the study accounted for prior
356 academic performance by analysing mid-year educational performance data from 2024 as
357 a basis for comparison, reducing the influence of pre-existing academic disparities.
358 **Teaching Quality and Learning Environment:** Individual teacher differences could
359 exist even within a school but significant disparities in teaching quality between
360 intervention and control schools were unlikely. Neither the intervention nor the control
361 schools had access to educational technologies, specialized training, or additional
362 resources. Both are located in similar rural environments, ensuring comparable learning
363 conditions.
364 **Cultural and Religious Homogeneity:**	Most sampled girls shared the same ethnic
365 background (98% Amhara) and faith (84% Orthodox), minimizing the potential effects of
366 cultural and religious differences on the study findings.

## 367 2.7 Data collection

368 The study team provided data collectors’ training, including pilot testing on March 7 and
369 8, 2024, respectively. This was followed by data collection that lasted from March 11 to
370 15, 2024.

## 371 2.8 Statistical methods used

372 The preparation for data analysis involved data cleaning, coding of qualitative and
373 quantitative data, and entering the data into spreadsheets. The study used analysis for
374 qualitative data, while SPSS software was employed for quantitative analysis. Descriptive
375 statistics, including frequencies and percentages were used to summarize the characteristics
376 of each question, and an independent samples t-test was conducted to evaluate statistical
377 significance. Finally, findings from qualitative and quantitative analyses were integrated to
378 assess the research hypotheses.

379

380

381

382

383

384

385

386

387

388

389

390

391

392

393

394

395

396

397

398

399

400

# 401 3. Results

## 402 3.1 Sample characteristics

403 The demographic analysis showed that girls between 13–15 years old accounted 69%,
404 followed by 16–18, 16%, and 10–12 years old that represented 15%. Regarding ethnicity
405 and religion, 98% of the participants identified as Amhara, while smaller proportions
406 identified as Agew (1%) and Tigre (0.8%). Regarding faith, 84% of the girls reported being
407 Orthodox, while 9% identified as Protestant and 6% as Muslim.
408 The result indicated that 66% of fathers and 52% of mothers were literate. Moreover, 82%
409 of parents depended on agriculture as their main source of income, while only 8% earned
410 money from business (Table 1).
411 Table 1: Demographic characteristics of the sample

| N=360 | Characteristics | Frequency | Percent |
| --- | --- | --- | --- |
|  | Female | 360 | 100 |
| Age | 10-12 | 55 | 15.3 |
|  | 13-15 | 248 | 68.9 |
|  | 16-18 | 56 | 15.6 |
|  | Above 19 | 1 | 0.3 |
| Religion | Orthodox | 302 | 83.9 |
|  | Islam | 22 | 6.1 |
|  | Protestant | 34 | 9.4 |
|  | Other | 2 | 0.6 |
| Ethnic group | Amhara | 353 | 98.1 |
|  | Agew | 4 | 1.1 |
|  | Tigre | 3 | 0.8 |
| Parents' occupation | Farmer | 294 | 81.7 |
|  | Merchant | 30 | 8.3 |
|  | Office worker | 16 | 4.4 |
|  | Other | 20 | 5.6 |
| Mothers' level of education | Abel to read and write | 144 | 40 |
|  | Grade 1-6 | 28 | 7.8 |
|  | Grade 7-12 | 14 | 3.9 |
|  | Not able to read and write | 172 | 47.8 |
|  | Other | 2 | 0.6 |
| Fathers' level of education | Abel to read and write | 183 | 50.8 |
|  | Grade 1-6 | 43 | 11.9 |
|  | Grade 7-12 | 14 | 3.9 |
|  | Not able to read and write | 118 | 32.8 |
|  | Other | 2 | 0.6 |

412

413

414

415

## 416 3.2 Empowering girls through school interventions

### 417 3.2.1 Qualitative results

418 Girls from intervention schools reported engaging in open discussions about MHM with
419 their mothers, sisters, male peers, and teachers. A *Girls’ Club* facilitator at an intervention
420 school stated, *"Girls no longer hesitate to ask for menstrual breaks during class."*
421 In contrast, girls in control schools indicated that their discussions about MHM were mostly
422 limited to female teachers. They felt uncomfortable talking about the topic with boys and
423 male teachers. A female teacher at a control school remarked, *"Girls lack sufficient*
424 *knowledge about MHM and often do not have the confidence to discuss the topic with their*
425 *teachers and peers."*

426

### 427 3.2.2 Quantitative results

428 The data revealed that 54% of girls in intervention schools felt confident purchasing
429 sanitary products, compared to only 18% in control schools. Additionally, 58% of girls in
430 control schools reported feeling shy while buying sanitary products, whereas this was the
431 case for only 31% of girls in intervention schools (Fig1 & S2 Fig).

432

433

434

435	**Figure 1**: **Comparing girls’ confidence while purchasing sanitary products**

436

437 The quantitative data on girls’ social interactions showed that 21% and 22% of girls from
438 intervention schools reported discussing MHM with boys, parents, and others, whereas only
439 9% of girls from control schools engaged in such conversations. Regarding access to MHM
440 education, 33% of girls from control schools stated they had not received any MHM
441 education, compared to just 2% of girls from intervention schools (Fig 2).

442

443

444

##### 445 Figure 2: Effects of school interventions on girls’ social interaction

446	The Independent Samples T-test showed that P-values for the differences between the two

447	groups of girls ranged mostly from 0.001 to 0.022. Consequently, P < α, since both 0.001

448	and 0.022 are below the significance level of 0.05 (Table 2).

449

450

451

452

453	Table 2: T-test results: Effects of school interventions on girls’ social interactions

| Independent samples T-test results | | | | | | | | | | |
| --- | --- | --- | --- | --- | --- | --- | --- | --- | --- | --- |
|  |  | Levene's Test for Equality of  Variances | | T-test for Equality of Means | | | | | | |
|  |  | *F* | *Sig.* | *t* | *df* | *Sig. (2-*  *tailed*  *)* | *Mean Differ ence* | *Std. Error Differ*  *ence* | *95% Confidence Interval of the Difference* | |
|  |  |  |  |  |  |  |  |  | *Lower* | *Uppe*  *r* |
| I have got courage to ask questions in class | Equal variances  assumed | 19.349 | 0.000 | 2.302 | 182 | 0.022 | 0.161 | 0.070 | 0.023 | 0.299 |
|  | Equal  variances not assumed |  |  | 2.319 | 181.924 | 0.022 | 0.161 | 0.069 | 0.024 | 0.298 |
| It Helped me improve communicati ons skill | Equal variances  assumed | 10.569 | 0.001 | 1.599 | 182 | 0.112 | 0.106 | 0.066 | -0.025 | 0.236 |
|  | Equal  variances not assumed |  |  | 1.612 | 181.983 | 0.109 | 0.106 | 0.066 | -0.024 | 0.235 |
| It helped me discuss with boys on MHM | Equal variances  assumed | 14.256 | 0.000 | 2.543 | 182 | 0.012 | 0.283 | 0.111 | 0.063 | 0.503 |
|  | Equal variances not  assumed |  |  | 2.684 | 117.885 | 0.008 | 0.283 | 0.105 | 0.074 | 0.492 |
| It helped me discuss with parents and others on MHM | Equal variances  assumed | 63.424 | 0.000 | 3.573 | 182 | 0.000 | 0.212 | 0.059 | 0.095 | 0.329 |
|  | Equal  variances not assumed |  |  | 3.667 | 169.380 | 0.000 | 0.212 | 0.058 | 0.098 | 0.326 |
| No change at all | Equal variances  assumed | 4.993 | 0.027 | - 1.107 | 182 | 0.270 | - 0.042 | 0.038 | -0.117 | 0.033 |
|  | Equal variances not  assumed |  |  | - 1.088 | 157.158 | 0.278 | - 0.042 | 0.039 | -0.118 | 0.034 |
| No MHM  education at school | Equal variances  assumed | 357.730 | 0.000 | - 6.501 | 182 | 0.000 | - 0.341 | 0.053 | -0.445 | - 0.238 |
|  | Equal  variances not assumed |  |  | - 6.179 | 103.847 | 0.000 | - 0.341 | 0.055 | -0.451 | - 0.232 |

454

455

456

457

## 458 3.3 Effects of school interventions on girls’ health

459 One of the district health centres, Shumshiha, serves the community, including female
460 students. Clinical data from September 2022 to February 2024 were analysed to assess the
461 effects of unsafe MHM practices on girls' health. During this period, 41 females sought
462 treatment at the centre for illnesses related to inadequate MHM practices, with 14 (34%) of
463 these patients being menstruating students. Clinical evidence indicated that the primary
464 causes of these illnesses were excessive sanitary pad use and insufficient knowledge of
465 proper MHM practices.
466 However, quantitative results showed that 43.8% of girls in intervention schools practised
467 genital hygiene three times a day during menstruation, compared to only 19% in control
468 schools. Additionally, 51.1% of girls in intervention schools reported cleaning their genitals
469 three to four times daily, while only 33.3% of girls in control schools did the same (Fig 3).

470

471

472 **Figure 3: Girls’ genital hygiene practice during menstruation**
473 Regarding the emotional and social aspects of girls' health, 58% of female students in
474 control schools reported feeling fearful when purchasing sanitary products, compared to
475 only 31% in intervention schools (Fig1). Furthermore, 21% of girls in intervention schools
476 stated that MHM education enabled them to discuss menstruation with boys, parents, and
477 others, compared to just 9% in control schools (Fig 2).
478 Data from the health centre indicated that water-borne diseases posed a significant
479 challenge for communities, especially affecting female students. During the reporting
480 period, the health centre treated 125 female patients, with 51 (41%) of them being students.
481 The most common water borne illness was diarrhoea mainly due to insufficient access to
482 clean water and water contamination. This finding was consistent with data from the district
483 education office, showing 70% of schools in the district did not have access to improved
484 water sources.
485 Observations of school facilities and hygiene services, along with feedback from focus
486 group discussions, revealed that two of the three intervention schools had access to
487 improved water sources. In stark contrast, none of the control schools had such access.
488 Data analysis showed that when water was available at schools, girls used it to support
489 MHM practices. For instance, 41% of girls in intervention schools used school water to
490 wash sanitary pads, and 17% for genital hygiene, whereas in control schools, the
491 corresponding figures were 24% and 8%, respectively (Fig 4).

492

493

494

495

496	**Figure 4: Benefits of school water for girls’ MHM practice**

497

498

499

500

516

| 501 |  |
| --- | --- |
| 502 | **3.4. Effects of school interventions on girls’ class attendance** |
| 503 | **3.4.1 Qualitative results** |
| 504 | Qualitative interviews revealed that menstruating girls and their mothers identified family |
| 505 | responsibilities, such as caring for a sick family member, participating in household chores |
| 506 | related to ceremonies, and personal illness—as the primary factors contributing to class |
| 507 | absenteeism**.** Neither the mothers nor the girls mentioned menstruation as a reason for |
| 508 | missing classes. |
| 509 | A female teacher serving as an MHM facilitator at an intervention school noted, |
| 510 | "Menstruating students do not miss classes during their period because they receive better |
| 511 | hygiene services at school than at home." |
| 512 | In contrast, teachers from control schools reported that girls who were menstruating |
| 513 | frequently missed classes due to a lack of MHM services. Furthermore, girls themselves |
| 514 | often hesitated to share the reasons for their absenteeism out of fear of stigma and |
| 515 | judgement. |

The Independent Samples T-test for water use revealed that, with one exception, all comparisons had p-values between 0.001 and 0.022, both of which are below the significance level of 0.05. Thus, P < α (0.001 < 0.05 and 0.022 < 0.05) (Table 3).

Table 3: T-test results: Multiple benefits of school water to menstruating girls

| Independent Samples Test results | | | | | | | | | | |
| --- | --- | --- | --- | --- | --- | --- | --- | --- | --- | --- |
|  |  | Levene's Test for Equality of  Variances | | **t-test for Equality of Means** | | | | | | |
|  |  | F | Sig. | t | df | Sig. (2-  tailed) | Mean Difference | Std. Error Difference | 95%  Confidence Interval of the Difference | |
|  |  |  |  |  |  |  |  |  | Lower | Upper |
| Menstrual pad washing | Equal variances  assumed | 5.779 | 0.017 | 5.668 | 182 | 0.001 | 0.386 | 0.068 | 0.252 | 0.52 |
|  | Equal variances  not assumed |  |  | 5.639 | 174.45 | 0.001 | 0.386 | 0.068 | 0.251 | 0.521 |
| Genital cleaning | Equal variances  assumed | 48.105 | 0 | 3.183 | 182 | 0.002 | 0.19 | 0.06 | 0.072 | 0.308 |
|  | Equal variances  not assumed |  |  | 3.256 | 173.37 | 0.001 | 0.19 | 0.058 | 0.075 | 0.305 |
| Drinking and hand washing | Equal variances  assumed | 48.809 | 0 | 3.493 | 182 | 0.001 | 0.232 | 0.067 | 0.101 | 0.364 |
|  | Equal variances  not assumed |  |  | 3.547 | 180.06 | 0.001 | 0.232 | 0.065 | 0.103 | 0.362 |
| Cleaning rooms | Equal variances  assumed | 5.106 | 0.025 | 1.117 | 182 | 0.266 | 0.074 | 0.066 | -  0.056 | 0.204 |
|  | Equal variances  not assumed |  |  | 1.123 | 181.63 | 0.263 | 0.074 | 0.065 | -  0.056 | 0.203 |
| No water at school | Equal variances  assumed | 407.451 | 0 | -9.438 | 182 | 0.001 | -0.517 | 0.055 | -  0.625 | -  0.409 |
|  | Equal variances  not assumed |  |  | -8.999 | 108.45 | 0.001 | -0.517 | 0.057 | -  0.631 | -  0.403 |

### 517 3.4.2 Quantitative results

518 The quantitative analysis revealed that 68.3% of girls in intervention schools attended
519 classes during menstruation, compared to only 29.8% in control schools (Fig 5).
520 For girls in control schools, the top three reported reasons for absenteeism during
521 menstruation were lack of sanitary pads (31%), lack of water (17%), and menstrual pain or
522 discomfort related to toilet facilities (11%). In contrast, girls in intervention schools cited
523 menstrual pain (9%), lack of water and sanitary pads (7.8%), and lack of comfortable toilet
524 facilities (6.4%) as the primary reasons for absenteeism.
525 Additionally, approximately 70.2% of girls in control schools reported absenteeism due to
526 MHM challenges, compared to just 31.7% in intervention schools (Fig 5).

527

528

529

530 **Figure 5: Girls’ class attendance during menstruation**
531 The independent samples t-test analysing differences in class absenteeism between girls in
532 intervention and control schools showed that nearly all characteristics had P-values
533 between 0.001 and 0.004, below the significance level of 0.05 (Table 4). Therefore, P < α
534 (0.001 < 0.05; 0.004 < 0.05).

535

536

537

538

539

540

541

542

543

544

545

546

547

548

549

550

551	Table 4: T-test results: Effects of school interventions on class attendance

| Independent sample test results | | | | | | | | | | |
| --- | --- | --- | --- | --- | --- | --- | --- | --- | --- | --- |
|  |  | Levene's Test for Equality  of Variances | | t-test for Equality of Means | | | | | | |
| How often do you go to school during menstruation? |  | F | Sig. | t | df | Sig. (2-  tailed) | Mean Difference | Std. Error Difference | 95% Confidence  Interval of the Difference | |
|  |  |  |  |  |  |  |  |  | Lower | Upper |
| Yes ,I often go to school | Equal variances  assumed | 30.100 | 0.000 | 5.104 | 182 | 0.000 | 0.345 | 0.068 | 0.212 | 0.479 |
|  | Equal variances not  assumed |  |  | 5.048 | 167.030 | 0.000 | 0.345 | 0.068 | 0.210 | 0.480 |
| No, I do not go due to lack of pads | Equal variances  assumed | 160.112 | 0.000 | -6.081 | 182 | 0.000 | -0.362 | 0.059 | -0.479 | -0.244 |
|  | Equal variances not  assumed |  |  | -5.886 | 132.432 | 0.000 | -0.362 | 0.061 | -0.483 | -0.240 |
| No, I do not go due to lack of water | Equal variances  assumed | 40.802 | 0.000 | -3.022 | 182 | 0.003 | -0.164 | 0.054 | -0.271 | -0.057 |
|  | Equal variances not  assumed |  |  | -2.945 | 144.171 | 0.004 | -0.164 | 0.056 | -0.274 | -0.054 |
| No, I do not go to due to unclean toilets | Equal  variances assumed | 20.063 | 0.000 | -2.164 | 182 | 0.032 | -0.103 | 0.048 | -0.197 | -0.009 |
|  | Equal variances not  assumed |  |  | -2.112 | 146.544 | 0.036 | -0.103 | 0.049 | -0.199 | -0.007 |
| No, I do not go to school, due to menstrual related pain | Equal variances  assumed | 3.996 | 0.047 | -0.996 | 182 | 0.321 | -0.051 | 0.051 | -0.151 | 0.050 |
|  | Equal variances not  assumed |  |  | -0.985 | 168.278 | 0.326 | -0.051 | 0.051 | -0.152 | 0.051 |

552

553	Additionally, girls were asked to rate the reasons for their class absenteeism, considering

554	both menstrual and non-menstrual periods. In control schools, girls identified family

555	responsibilities as the primary reason for absenteeism (38%), followed by a lack of sanitary

556	pads (26%) and menstrual pain or other illnesses (18%) (Fig6).

557	In contrast, girls in intervention schools ranked menstrual pain as the leading reason for

558	absenteeism (31%), closely followed by family responsibilities (30%) and other illnesses

559	(21%) (Fig 6).

560

561

562	**Figure 6: Major factors for girls’ class absenteeism - menstrual and non-menstrual periods**

563

## 564 3.5 Effects of school interventions on academic performance

### 565 3.5.1 Girls’ study time

566	The study found that 77.6% of girls in intervention schools reported having

567	sufficient time to study at home, compared to only 41.1% in control schools.

568	Additionally, 53% of girls in control schools indicated a lack of study time due to

569	family responsibilities, while only 10.9% of girls in intervention schools cited the

570	same reason (Fig 7).

571

572

573	**Figure 7: Girls’ study time at home**

574

575	The P-value for the study time difference between the two groups of girls is 0.011, which

576	is less than the significance level. Therefore, P < α; 0.011 < 0.05 (Table 5).

577	Table 5: T-test results on girls’ study time

Independent Samples Test

|  |  | Levene's Test for Equality  of Variances | | t-test for Equality of Means | | | | | |  |
| --- | --- | --- | --- | --- | --- | --- | --- | --- | --- | --- |
|  |  | F | Sig. | t | df | Sig. (2- | Mean | Std. | 95% Confidence | |
|  |  |  |  |  |  | tailed) | Differe | Error | Interval | |
|  |  |  |  |  |  |  | nce | Differe |  | |
| Do you |  |  |  |  |  |  |  | nce |  | |
| have |  |  |  |  |  |  |  |  |  |  |
|  |  |  |  |  |  |  |  |  | Lower | Upper |
| enough time to study at home? |  |  |  |  |  |  |  |  |  |  |
|  | Equal variances assumed | 0.846 | 0.358 | -3.522 | 358 | 0.011 | -0.295 | 0.084 | -0.460 | -0.130 |
|  | Equal |  |  | -3.560 | 357.740 | 0.010 | -0.295 | 0.083 | -0.459 | -0.132 |
|  | variances |  |  |  |  |  |  |  |  |  |
|  | not assumed |  |  |  |  |  |  |  |  |  |

578

579

580

### 581 3.5.2 Academic performance

582 To compare the educational performance of girls in intervention and control schools, the
583 average score for each group was calculated over a six-month period**.** In intervention
584 schools, 333 girls (both menstruating and non-menstruating) were assessed. The results
585 showed that 2% scored "excellent," 4% scored "very good," 14% scored "good," and 29%
586 scored "fair" (Fig 8**).**
587 In contrast, in the control schools, where 293 girls were assessed, the results were as
588 follows: 3% were rated as “excellent," 9% as "very good," 16% as "good," and as 25%
589 "fair." These ratings are based on the standards set by the Ethiopian Ministry of Education
590 for primary and secondary school students. However, a similar proportion of girls from
591 both groups received ratings of "good" or "fair": 43% in intervention schools compared to
592 41% in control schools (Fig 8).

593

594

595

596

597

598

599

600

601	**Figure 8: First semester academic performance**

602

603

604

605

606

607

608

609

610

611

# 612 4. Discussion

613 The demographic analysis revealed that girls between the ages of 13–15 years accounted
614 for 69%, followed by those aged 16–18; at 16%, and girls aged 10–12 representing 15%.
615 In terms of ethnicity and religion, 98% of the participants identified as Amhara, with
616 smaller proportions identifying as Agew (1%) and Tigre (0.8%). In terms of faith, 84% of
617 the girls reported being Orthodox, while 9% identified as Protestant and 6% as Muslim.
618 Parental education levels were low, with 66% of fathers able to read and write, compared
619 to 52% of mothers. Household income was primarily dependent on agriculture (82%), with
620 a smaller proportion (8.3%) engaged in business (Table 1).
621 **Empowering girls through school interventions:** Access to adequate MHM knowledge,
622 skills, and WASH services is crucial for enhancing the confidence and empowerment of
623 menstruating girls. Qualitative findings from focus group discussions with mothers,
624 menstruating girls, and teachers revealed that girls in intervention schools felt more
625 confident discussing MHM with peers, teachers, and mothers compared to those in control
626 schools.
627 Similarly, quantitative results showed that 54% of girls in intervention schools felt
628 confident purchasing sanitary products, compared to only 18% in control schools.
629 Conversely, 41% of girls in control schools reported feeling fearful while buying sanitary
630 products compared to only 18% in intervention schools (Fig 1).
631 Regarding social interactions, 21% of girls in intervention schools discussed MHM with
632 their peers, including boys, and 22% with their parents, whereas only 9% of girls in control
633 schools engaged in such discussions (Fig 2).
634 The difference was further analysed using the Independent Samples T-test, which
635 confirmed that the variation was statistically significant, with a P-value of 0.001. Since P
636 < α (0.001 < 0.05), the results indicate a significant difference in the empowerment of
637 menstruating girls between intervention and control schools (Table 2).
638 The significant difference in girls’ empowerment between intervention and control schools
639 was primarily due to disparities in access to MHM education and school WASH services.
640 The school interventions enhanced girls' MHM knowledge and skills while also providing
641 opportunities to discuss MHM with peers, teachers, and parents, further increasing their
642 confidence when purchasing sanitary products. Additionally, improved school WASH
643 services enabled girls to practice safe MHM, reducing anxiety about potential bloodstains
644 and allowing them to focus more on their education.
645 The findings of this study align with an assessment report by UNICEF, Plan International,
646 Save the Children, and Emory University, which identified WASH facilities, MHM
647 knowledge, practical guidance, and access to sanitary products as key factors contributing
648 to girls’ empowerment [10]. The report further emphasized that the absence of these
649 services could lead to unsafe MHM practices, confusion, anxiety about staining clothes,
650 and teasing by boys.
651 Moreover, consecutive proceedings by UNICEF and Columbia University over the past
652 decade have recognized school WASH interventions as essential for empowering girls'
653 education [11]. Additionally, studies on MHM and class attendance in Niger and Uganda
654 have demonstrated that improved school infrastructure, MHM education, and access to
655 WASH services bring significant positive changes in girls’ knowledge and MHM practices
656 [5, 27].

##### 657 Effects of school interventions on girls’ physical, emotional, and social health: A lack

658 of school-based MHM education and inadequate WASH services exposes girls to unsafe
659 hygiene practices, leading to confusion, anxiety, and negative effects on their physical,
660 emotional, and social well-being [10].
661 Health centre data from September 2022 to February 2024, focusing on MHM and water
662 borne diseases, indicate that limited MHM knowledge and excessive use of sanitary
663 products contributed to genital bruising and related discomfort among women and female
664 students. During this period, menstruating girls comprised 34% of the health centre’s
665 patients.
666 However, school-based MHM education and WASH interventions improved girls’ MHM
667 knowledge and practices, positively influencing their health. The study found that 44% of
668 girls in intervention schools practised genital hygiene three times per day during
669 menstruation, compared to only 19% in control schools. Additionally, 51% of girls in
670 intervention schools practised genital hygiene three to four times daily, whereas only 33%
671 in control schools did the same (Fig 3).
672 Moreover, while 58% of girls in control schools reported feeling shy or fearful when
673 purchasing sanitary products, this figure dropped to 31% among girls in intervention
674 schools. The results suggest that the school intervention nearly halved these fears,
675 contributing to better emotional health among girls in intervention schools (Fig 1).
676 Furthermore, 21% and 22% of girls in intervention schools felt comfortable discussing
677 MHM with boys and parents, respectively, compared to only 9% in control schools. This
678 finding suggests improved social health among girls in intervention schools (Fig. 2).
679 The school interventions also improved water access in most intervention schools, allowing
680 girls to practice safe menstrual hygiene management.	For example, 41% of girls in
681 intervention schools used water for washing sanitary pads, and 17% for genital hygiene,
682 compared to 24% and 8% in control schools, respectively (Fig 4).
683 The disparity in water usage for safe MHM practices between intervention and control
684 schools was statistically significant, with a P-value of 0.001, which is less than 0.05.
685 Therefore, since P < α (0.001 < 0.05) (Table 3); this suggests a significant distinction
686 between the two groups in using school water to promote safe MHM practices.
687 These research findings align with studies from Bangladesh and Nepal, which reported that
688 access to MHM education and WASH services reduces girls’ stress and discomfort while
689 improving hygiene practices [3]. Similarly, a study in India found that access to WASH
690 services and improved MHM practices helps mitigate negative health effects, such as
691 infections associated with reusable sanitary products [24].
692 However, the health centre report from the study district, which identified diarrhoea, as a
693 WASH-related challenge affecting students, including girls, was not supported by either
694 the qualitative or quantitative findings of this study. In fact, female students ranked
695 diarrhoea as the least significant cause of class absenteeism (Fig 6).
696 This discrepancy may be linked to cultural and educational factors. In the study district and
697 the broader region, the word "diarrhoea" carries a negative connotation, as the local term
698 (used during data collection) is often used as an insult. This cultural stigma may have made
699 focus group participants hesitant to discuss the issue openly.
700 Another possible explanation is that mothers and girls may not distinguish diarrhoea as a
701 specific health concern, instead referring to it as "sickness." This could explain why girls
702 ranked class absenteeism due to illness under the broader category of "other sickness." In
703 both intervention and control schools, girls ranked "other sickness" fourth—after family
704 responsibilities, lack of sanitary pads, and menstrual hygiene-related pain—among six
705 possible reasons for absenteeism. Thus, diarrhoea may have been overlooked or classified
706 under this broader category (Fig 6).
707 Overall, the research findings indicate that school interventions can enhance girls'
708 emotional, social, and physical well-being, reinforcing the importance of implementing
709 MHM and WASH programs in schools.
710 **Effects of school interventions on class attendance:** Qualitative findings indicate that
711 family responsibilities, such as caregiving for sick relatives, girls’ own health conditions,
712 and menstrual-related pain, were the primary reasons for class absenteeism rather than
713 menstruation alone. However, teachers from both intervention and control schools
714 attributed absenteeism more to the availability of MHM and WASH services at school.
715 A teacher facilitating an MHM club in an intervention school stated, “During menstruation,
716 girls prefer coming to school because they receive better MHM services here than at home.”
717 Similarly, a school principal and mothers of girls from intervention schools acknowledged
718 that access to MHM services at school helped prevent absenteeism during menstruation.
719 In contrast, mothers of girls from control schools believed that the lack of MHM services,
720 such as water and sanitary products, contributed to absenteeism. Female students echoed
721 this concern, stating, “Most girls do not change sanitary pads at school due to lack of access
722 to water, sanitary pads, and girl-friendly toilets.”
723 Additionally, a school principal and an MHM club facilitator from a control school
724 explained, “Although girls do not explicitly say they are absent due to menstruation, we
725 believe inadequate MHM services contribute to their absenteeism during this time.”
726 The quantitative results further clarified the qualitative findings, showing that 68.3% of
727 girls in intervention schools attended classes during menstruation, compared to only 29.8%
728 in control schools. This indicates that access to MHM services in intervention schools
729 reduced absenteeism by more than half compared to control schools (Fig 5).
730 Additionally, nearly 48% of girls in control schools reported missing classes due to a lack
731 of sanitary products and water, whereas only 15.6% of girls in intervention schools cited
732 the same reason (Fig 5).
733 Absenteeism was also related to social interactions, as teasing by boys and others
734 contributed to girls’ absences. In intervention schools, 21% of girls felt comfortable
735 discussing MHM with boys, compared to only 9% in control schools. Furthermore, 22% of
736 girls in intervention schools believed MHM education enabled them to discuss
737 menstruation with parents and others, whereas only 9% of girls in control schools shared
738 this view (Fig 2).
739 The qualitative findings highlight the critical role of the school environment in supporting
740 MHM practices, while the quantitative results provide measurable insights into their effect
741 on attendance. An Independent Samples T-test further examined this difference, yielding a
742 P-value of 0.001, well below the 0.05 threshold (Table 4). This statistically significant
743 difference in attendance can largely be attributed to access to MHM education and WASH
744 services in intervention schools, which were absent in control schools.
745 The findings align with a study from the Tigray region of northern Ethiopia, which
746 demonstrated improved MHM practices and increased class attendance among girls
747 following the provision of MHM education, sanitary products, and underwear [8].
748 Similarly, research in Ghana reported higher-class attendance among girls after providing
749 sanitary products and MHM education [14]. Additionally, a study in Kenya found that
750 access to school WASH services significantly reduced absenteeism among primary school
751 students [20].
752 However, these findings contrast with those of two other studies. Research in Nepal, for
753 instance, found no improvement in girls' attendance despite the provision of menstrual cups
754 to address sanitary product shortages [9]. Likewise, a study in Malawi reported that
755 variations in access to WASH services across schools did not significantly influence girls'
756 absenteeism [19].
757 The differences in context may explain the contrasting results. The Nepal study solely
758 focused on providing menstrual cups without incorporating other essential factors, such as
759 MHM education and WASH services, which play a crucial role in shaping hygiene
760 behaviour. It was a single-factor intervention rather than a comprehensive approach.
761 Similarly, the Malawi study did not emphasize behaviour change promotion related to
762 school WASH services. Although some schools had WASH facilities as part of broader
763 social services, they were not designed to influence girls' hygiene practices.
764 These findings suggest that improving girls’ MHM practices and class attendance requires
765 integrated school-based interventions that address the specific needs of menstruating girls.
766 This includes fostering supportive social environments both within and beyond school
767 settings, as well as establishing a favourable policy framework to sustain these efforts.
768 However, access to WASH services alone is insufficient to improve class attendance. In
769 order to be effective, school WASH services should target specific behavioural
770 improvements. This requires active involvement from all stakeholders, including school
771 community members.
772 The findings indicate that menstrual hygiene-related absenteeism can be reduced through
773 comprehensive, targeted MHM education and WASH interventions. However, school-
774 based efforts alone cannot eliminate absenteeism due to other contributing factors, such as
775 household chores. Lastly, menstruation should not be examined as a sole cause of
776 absenteeism but in relation to access to school-based MHM services to provide a more
777 accurate and holistic understanding of the issue.
778 **Effects of school interventions on academic performance:** The discussion on girls’
779 absenteeism suggests that school interventions can improve class attendance and reduce
780 menstrual-related absences. Additionally, the study found that these interventions enhanced
781 girls' study time at home. Specifically, 78% of girls in intervention schools reported having
782 sufficient study time, compared to 41% in control schools. In contrast, 53% of girls in
783 control schools cited household responsibilities as a barrier to studying, whereas only 11%
784 of girls in intervention schools faced the same challenge (Fig 7).
785 The findings indicate that school interventions nearly doubled girls' study time at home and
786 reduced their household burden by about five times compared to control schools. This
787 improvement was largely due to the empowerment provided by these interventions, which
788 equipped girls with the confidence and communication skills to negotiate with their
789 parents—an opportunity not available to girls in control schools.
790 Further analysis using the Independent Samples T-test yielded a P-value of 0.001, well
791 below the 0.05 threshold (Table5), indicating a statistically significant difference
792 attributable to the interventions.
793 A key question that arises is whether the improved class attendance, increased study time,
794 and other positive effects of the interventions translated into better academic performance
795 for girls in the intervention schools.
796 To evaluate academic performance, the study analysed half-year average scores,
797 categorizing them as "excellent," "very good," "good," or "fair" based on school ratings.
798 Among 333 girls in intervention schools, 2%, 4%, 14%, and 29% received these ratings,
799 respectively. In comparison, among 293 girls in control schools, the corresponding
800 percentages were 3%, 9%, 16%, and 25% (Fig 7).
801 Overall, 12% of girls in control schools received "very good" or "excellent" ratings,
802 compared to only 6% in intervention schools—half the proportion observed in control
803 schools. However, for lower ratings ("good" and "fair"), girls in the intervention schools
804 performed slightly better than their counterparts (43% versus 41%), though the difference
805 was not significant.
806 If first-semester average scores determined grade promotion, 49% (163 out of 333) of
807 menstruating girls in intervention schools would advance, compared to 53% (154 out of
808 293) in control schools.
809 These findings suggest that high-performing students are affected by class attendance and
810 study time, whereas the effect on lower-performing students is minimal. This implies that
811 other factors aside from attendance and study time play a role for the variations in academic
812 performance. However, it is important to note that the comparison was made using half-
813 year academic performance data, which may have influenced the results.
814 These findings contrast with previous research showing a positive correlation between class
815 attendance and academic performance [21, 22]. For example, *Class Attendance and*
816 *Learning Outcomes* found that attendance significantly improved performance among low-
817 achieving students [23]. While this partially aligns with low-performing girls in
818 intervention schools, the difference was not statistically significant.

819

# 820 5. Conclusion

821 School-based MHM and WASH interventions provide multiple benefits for menstruating
822 girls. They help build confidence in communicating with peers, teachers, and parents,
823 enhance MHM skills, and improve girls' ability to negotiate for study time and reduced
824 domestic workload.
825 Additionally, these interventions support physical, emotional, and social well-being by
826 promoting safe MHM practices, emotional regulation, and positive social interactions.
827 They also reduce class absenteeism by providing sanitation and hygiene facilities and
828 fostering supportive school environments, though domestic responsibilities and menstrual
829 pain remain significant barriers.
830 Despite improvements in attendance, study time, and social engagement, the impact on
831 academic performance is minimal, suggesting that other factors influence learning
832 outcomes.
833 The findings emphasize the need for comprehensive, sustained interventions tailored to
834 girls’ diverse needs to improve MHM practices, empowerment, health, and school
835 attendance. Continued support from school programs is essential for long-term adoption of
836 MHM practices and resilience to related challenges.
837 This study contributes to closing knowledge gaps in MHM and absenteeism research,
838 particularly regarding girls' empowerment, health, and academic performance, and serves
839 as a foundation for future studies.
840 Finally, the findings have implications for policy makers and organizations involved in
841 girls' education. Ethiopia has a supportive policy framework for school MHM and WASH
842 interventions, but inadequate budget allocation limits implementation. Regional and district
843 WASH sector bureaus should allocate sufficient funds to improve these services.
844 Non-governmental organizations, as key drivers of school MHM and WASH initiatives,
845 should strengthen collaboration with government agencies, actively engage girls, and
846 design comprehensive school WASH programs.

847

848

849

850	**Acknowledgements:** The authors acknowledge Ato Yalew Tizazu and Wondemu Sisay

851	for their support in data collection. We also appreciate Lasta District Education Office for

852	approving the research and teachers for facilitating the data collection process.

853

854

### 855 6. References

856 1.	Ministry	of	Health.	National	adolescent	and	youth	health	strategy.	2018;	Available
857 from:[https://www.prb.org/wp-content/uploads/2018/05/National-Adolescent-and-Youth-Health-](https://www.prb.org/wp-content/uploads/2018/05/National-Adolescent-and-Youth-Health-%20Strategy)
858 [Strategy](https://www.prb.org/wp-content/uploads/2018/05/National-Adolescent-and-Youth-Health-%20Strategy)
859 2. Crofts T, Coates S, Fisher J. Menstruation hygiene management for school girls. 2014; Available from:
860 <http://wedc.lboro.ac.uk/wedc.lboro.ac.uk/knowledge>
861 3. Bhusal CK. Practice of menstrual hygiene and associated factors among adolescent school girls in Dang
862 District, Nepal. *Advances in Preventive Medicine*. 2020; http://doi:10.1155/2020/1292070.
863 4. Tamiru S, Mamo K, Acidria P, Mushi R, Ali CS, Ndebele L. Towards a sustainable solution for school
864 menstrual hygiene management: Cases of Ethiopia, Uganda, South-Sudan, Tanzania, and Zimbabwe.
865 Sahin M, editor. Waterlines. 2015;34[:http://*doi.org/10.3362/1756-3488.2015.009*](http://doi.org/10.3362/1756-3488.2015.009)
866 5. Seydou F, Ama-dou B, Abdoulaye S, Alassane T, Oumar TS, Ibrahima K, et al. Menstrual hygiene
867 management in school setting in two secondary schools in the Bamako District, Case of the
868 School	“La	Chaine	Grise”	and	the	school	cheick	Modibo	Diarra. 2020; http://
869 doi.org/10.4236/ojog.2020.1020025
870 6. Sommer M, Hirsch JS, Nathanson C, Parker RG. Comfortably, safely, and without shame: Defining
871 mmenstrual hhygiene mmanagement as a ppublic hhealth iissue. American Journal of Public Health.
872 2015; <https://ajph.aphapublications.org/doi/full/10.2105/AJPH.2014.302525>
873 7. Tegegne TK, Sisay MM. Menstrual hygiene management and school absenteeism among female
874 adolescent students in North-east Ethiopia. BMC Public Health. 2014; 14:http// [doi/10.1186/1471-2458-](https://doi/10.1186/1471-2458-14-1118)
875 [14-1118](https://doi/10.1186/1471-2458-14-1118)
876 8. Belay S, Kuhlmann AKS, Wall LL. Girls’ attendance at school after a menstrual hygiene intervention in
877 northern Ethiopia. International Journal of Gynaecology & Obstetrics. 2020; 149:287–91. http:// doi:
878 10.1002/ijgo.13127
879 9. Oster E, Thornton R. Menstruation, sanitary products, and school attendance: Evidence from a randomized
880 evaluation. American Economic Journal: Applied Economics. 2011; 3: 91–100. [http://doi:](http://doi/)
881 10.1257/app.3.1.91
882 10. Haver J, Caruso BA, Ellis A, Sahin M, Villasenor JM, Ande KL, Freeman MC. WASH in school
883 empowers girls’ eeducation in Masbate Province and Metro Manila, Philippines: An assessment of
884 menstrual hygiene management in schools. 2013; available from [https://www.unicef.org/wash/schools.](https://www.unicef.org/wash/schools)
885 11. United Nations Children’s Fund and Columbia University. WASH in schools empowers girls’ education: Proceedings
886 of the annual virtual conference on menstrual	hygiene management in schools.2017;available from
887 https:/[/www.washinschoolsindex.com](http://www.washinschoolsindex.com/) [/storage/articles/ck1he0Zuxts5R7dNgMiPtrRF2SuCKjP54b3VbjnA.](https://www.washinschoolsindex.com/storage/articles/ck1he0Zuxts5R7dNgMiPtrRF2SuCKjP54b3VbjnA)
888 12. Woodall J, Raine G, South J, Warwick-Both L. Empowerment , health and well-being. 2010; available
889 from <https://core.ac.uk/download/pdf/42412714.pdf>
890 13. Prüss-Üstün A. Safer water, better health: Costs, benefits and sustainability of interventions to protect and
891 promote health. 2008; available from <https://apps.who.int/iris/handle/10665/43840>
892 14. Samuel KA, Ebenezer EYA, Nang BD, George O, Charles CA, et al. WASH in selected basic schools
893 and possible implications on health and academics: An example of the Wa Municipality of Ghana,
894 West	Africa. American Journal of Environmental Science and Engineering. 2021; 5:15-20. http://
895 [doi.org/10.11648/j.ajese.20210501.13](https://doi.org/10.11648/j.ajese.20210501.13)
896 15. Birdthistle I, Dickson K, Freeman M, Javidi L. What impact does the provision of separate toilets for girls
897 at schools have on their primary and secondary school enrolment, attendance and completion? A systematic
898 review of the evidence. EPPI-Centre, Social Science Research Unit, Institute of Education, University of
899 London. 2011; ISBN: 978-907345: available from https[;//WWW.educ-links.org](http://WWW.educ-links.org/)
900 16. Jasper C, Le T-T, Bartram J. Water and sanitation in schools: A systematic review of the health and
901 educational outcomes. Research Gate. 2012; http:// doi 10.3390/ijerph9082772.
902 17.World	Health	Organization.	Basic	Documents,49ed.	2020;	available	from
903 [*https://apps.who.int/gb/bd/pdf_files/B*](https://apps.who.int/gb/bd/pdf_files/BD_49th-en.pdf%20.V12)	*D_49th-en.pdf .V12*
904 18. Oster E, Thornton R. Menstruation, sanitary products, and school attendance: Evidence from a randomized
905 evaluation. American Economic Journal.2011;3:91–100.[http://doi:](http://doi/) 10.1257/app.3.1.91
906 19. Grant MJ, Lloyd CB, Mensch BS. Menstruation and school absenteeism: Evidence from rural Malawi.
907 Comparative Education Review. 2013; http:// doi: 10.1086/669121
908 20. Freeman MC, Greene LE, Dreibelbis R, Saboori S, Muga R, Brumback B, et al. Assessing the impact of a
909 school-based water treatment, hygiene and sanitation programme on pupil absence in Nyanza Province,
910 Kenya: A cluster-randomized trial. Tropical Medicine & International Health. 2011; http://
911 doi:10.1111/j.1365-3156.2011.02927.x
912 21. [Tetteh GA.](https://www.emerald.com/insight/search?q=Godson%20Ayertei%20Tetteh) Effects of classroom attendance and learning strategies on the learning Outcome. *[Journal](c:\\pdfbuilder\\temp\\Journal of International Education  in  Business)*
913 *[of International Education in Business](c:\\pdfbuilder\\temp\\Journal of International Education  in  Business)*[.](c:\\pdfbuilder\\temp\\Journal of International Education  in  Business) 2018; 11: http:// [doi.org/10.1108/JIEB-01-2017-0004](https://doi.org/10.1108/JIEB-01-2017-0004)
914 22. Al Hazaa K , Abdel-Salam,ASG, Ismail R, Johnson C, Al-Tameemi RAN, Romanowski MH, et al. The
915 effects of attendance and high school GPA on student performance in first-year undergraduate
916 courses. *Cogent Education. 2021;8*: <https://doi.org/10.1080/2331186X.2021.1956857>
917 23. *Kwak DW, Sherwood C, Tang KK*. [**C**lass attendance and learning outcom**e**:](https://ideas.repec.org/a/spr/empeco/v57y2019i1d10.1007_s00181-018-1434-7.html) [Empirical economics.](https://ideas.repec.org/s/spr/empeco.html) 2019;

918	57: 177-203. [http://doi:](http://doi/) 10.1007/s00181-018-1434-7

919 24. Das P, Baker KK, Dutta A, Swain T, Sahoo S, Das BS, et al. Menstrual hygiene practices, WASH
920 access and the risk of urogenital infection in women from Odisha, India. Journal.pone.2015; http://
921 doi: 10.1371/journal.pone.0130777: PMC4488331.
922 25. Glanz K, Rimer BK, Viswanath K. Health behaviour and health education: Theory, research and practice.
923 **Jo**ssey-Bass.2008; <https://psycnet.apa.org/fulltext/2008-17146-000.pdf>
924 26. Krejcie RV, Morgan DW. Determining sample size for research activities: Educational and psychological

925	measurement. Sage publications.1970;30:607–10.http:// doi/10.1177/001316447003000308

926 27. Miiro G, Rutakumwa R, Nakiyingi-Miiro J, Nakuya K, Musoke S, Namakula J, et al. Menstrual health and
927 school absenteeism among adolescent girls in Uganda: A feasibility study. BMC Women’s Health. 2018;
928 18: http:// doi 10.1186/s12905-017-0502-z
929 28. Sumpter C, Torondel B. A systematic review of the health and social effects of menstrual hygiene
930 management. PLoS ONE. 2013;8: http:// doi:10.1371/journal.pone.0062004

931

932

933

934

935

936

937

938

939

940

941

942

943

944

945

946

947

948

949

950

951

952

953

954

955

### 956 7. Supporting documents

957 **S1 Figure. *Girls’ knowledge on menstruation***
958 Girls from intervention schools benefited from school peer-to-peer education and teachers who
959 took MHM trainings. This helped girls from intervention school to demonstrate improved
960 hygiene knowledge and practice compared to non-intervention schools who lucked these
961 opportunities.
962 **S2 Figure: Girls’ confidence to request menstrual break during classes**
963 Menstruating girls from intervention school improved their social relations with boys and
964 teachers due to the school intervention and this was reflected in their confidence to request
965 their teachers, male and female, for menstrual break during classes, unlike the non-
966 intervention school girls who were limited to female teachers***.***
967 *S1Table: G*irls’ experience to change sanitary pads at schools
968 In non-intervention schools, girls lacked access to dedicated clubrooms where they could change sanitary
969 pads, rest when feeling unwell and exchange hygiene education. This absence significantly impacted their
970 comfort and support for managing menstrual health at school, leading to a much higher proportion of girls
971 refraining from changing sanitary pads during school hours—44% compared to just 4.2% of the girls from
972 intervention schools.

Figure	[Click here to access/download;Figure;Fig 1.tif](https://www2.cloud.editorialmanager.com/pone/download.aspx?id=39268067&guid=2844abbb-4c71-4937-a0c9-eabec99c4169&scheme=1)[<https://www2.cloud.editorialmanager.com/pone/download.aspx?id=39268067&guid=2844abbb-4c71-4937-a0c9-eabec99c4169&scheme=1>](https://www2.cloud.editorialmanager.com/pone/download.aspx?id=39268067&guid=2844abbb-4c71-4937-a0c9-eabec99c4169&scheme=1)

Figure	[Click here to access/download;Figure;Fig 2.tif](https://www2.cloud.editorialmanager.com/pone/download.aspx?id=39268068&guid=6c410464-7b53-4f79-94be-fd34732e9d5f&scheme=1)[<https://www2.cloud.editorialmanager.com/pone/download.aspx?id=39268068&guid=6c410464-7b53-4f79-94be-fd34732e9d5f&scheme=1>](https://www2.cloud.editorialmanager.com/pone/download.aspx?id=39268068&guid=6c410464-7b53-4f79-94be-fd34732e9d5f&scheme=1)

Figure	[Click here to access/download;Figure;Fig 3.tif](https://www2.cloud.editorialmanager.com/pone/download.aspx?id=39341358&guid=3fcad909-402c-4126-9f3c-e91d11a54c01&scheme=1)[<https://www2.cloud.editorialmanager.com/pone/download.aspx?id=39341358&guid=3fcad909-402c-4126-9f3c-e91d11a54c01&scheme=1>](https://www2.cloud.editorialmanager.com/pone/download.aspx?id=39341358&guid=3fcad909-402c-4126-9f3c-e91d11a54c01&scheme=1)

Figure	[Click here to access/download;Figure;Fig 4.tif](https://www2.cloud.editorialmanager.com/pone/download.aspx?id=39268071&guid=2c9ca089-2bad-4a2e-8d67-4f9be90bb4b6&scheme=1)[<https://www2.cloud.editorialmanager.com/pone/download.aspx?id=39268071&guid=2c9ca089-2bad-4a2e-8d67-4f9be90bb4b6&scheme=1>](https://www2.cloud.editorialmanager.com/pone/download.aspx?id=39268071&guid=2c9ca089-2bad-4a2e-8d67-4f9be90bb4b6&scheme=1)

Figure	[Click here to access/download;Figure;Fig 5.tif](https://www2.cloud.editorialmanager.com/pone/download.aspx?id=39268074&guid=d1737892-b954-420d-bbec-555ce9c08268&scheme=1)[<https://www2.cloud.editorialmanager.com/pone/download.aspx?id=39268074&guid=d1737892-b954-420d-bbec-555ce9c08268&scheme=1>](https://www2.cloud.editorialmanager.com/pone/download.aspx?id=39268074&guid=d1737892-b954-420d-bbec-555ce9c08268&scheme=1)

Figure	[Click here to access/download;Figure;Fig 6.tif](https://www2.cloud.editorialmanager.com/pone/download.aspx?id=39268075&guid=56bb9d7a-e1de-4b30-ae6f-a7c2bce892bd&scheme=1)[<https://www2.cloud.editorialmanager.com/pone/download.aspx?id=39268075&guid=56bb9d7a-e1de-4b30-ae6f-a7c2bce892bd&scheme=1>](https://www2.cloud.editorialmanager.com/pone/download.aspx?id=39268075&guid=56bb9d7a-e1de-4b30-ae6f-a7c2bce892bd&scheme=1)

Figure	[Click here to access/download;Figure;Fig 7.tif](https://www2.cloud.editorialmanager.com/pone/download.aspx?id=39268078&guid=1b460eb5-22f7-4bd0-a094-135ad5d3612d&scheme=1)[<https://www2.cloud.editorialmanager.com/pone/download.aspx?id=39268078&guid=1b460eb5-22f7-4bd0-a094-135ad5d3612d&scheme=1>](https://www2.cloud.editorialmanager.com/pone/download.aspx?id=39268078&guid=1b460eb5-22f7-4bd0-a094-135ad5d3612d&scheme=1)

Figure	[Click here to access/download;Figure;Fig 8.tif](https://www2.cloud.editorialmanager.com/pone/download.aspx?id=39268080&guid=79aef0cd-a2f8-4084-90ec-2ae8fd1b5af0&scheme=1)[<https://www2.cloud.editorialmanager.com/pone/download.aspx?id=39268080&guid=79aef0cd-a2f8-4084-90ec-2ae8fd1b5af0&scheme=1>](https://www2.cloud.editorialmanager.com/pone/download.aspx?id=39268080&guid=79aef0cd-a2f8-4084-90ec-2ae8fd1b5af0&scheme=1)

S1Figure

[Click here to access/download](https://www2.cloud.editorialmanager.com/pone/download.aspx?id=39341376&guid=14d72244-362b-4c13-8ce6-a1caf6b43c5c&scheme=1)

[**Supporting Information** S1 Figure.docx](https://www2.cloud.editorialmanager.com/pone/download.aspx?id=39341376&guid=14d72244-362b-4c13-8ce6-a1caf6b43c5c&scheme=1)

S2Figure

[Click here to access/download](https://www2.cloud.editorialmanager.com/pone/download.aspx?id=39341387&guid=40afa2d3-9938-48ce-a0a6-f16dc1a26c02&scheme=1)

[**Supporting Information** S2 Figure.docx](https://www2.cloud.editorialmanager.com/pone/download.aspx?id=39341387&guid=40afa2d3-9938-48ce-a0a6-f16dc1a26c02&scheme=1)

S1Table

[Click here to access/download](https://www2.cloud.editorialmanager.com/pone/download.aspx?id=39341494&guid=2b593e80-4a9c-46cb-9790-c4d4d89ee437&scheme=1)

[**Supporting Information** S1 Table.docx](https://www2.cloud.editorialmanager.com/pone/download.aspx?id=39341494&guid=2b593e80-4a9c-46cb-9790-c4d4d89ee437&scheme=1)

Revised Manuscript with Track Changes

1

2

3	**Effects of school menstrual hygiene management, water ~~and~~**

4	**sanitation and hygiene interventions on girls’ empowerment,**

5	**health and educational outcomes: Lasta District, Amhara**

6	**Regional State, Ethiopia**

7

8

##### 9 Effects of school based menstrual hygiene management

10	**interventions on girls’ empowerment**

11

###### 12 Fisseha A.Andargie^1^*^¶^, Femi R.Tinuola^2^*&*

13 ^1^Department of Public Health Administration, Central University of Nicaragua,
14 Managua,Nicaragua
15 ^2^Department of Sociology, Federal University Gusau, Zamfara State, Nigeria

16

17

18 *Corresponding author E-mail: [fissehaatalie@yahoo.com](mailto:fissehaatalie@yahoo.com) (FA);
19 ^¶^This author contributed to the article
20 *^&^*This author contributed to the article*.*

21

22

23

24

25

26

27

28

29

30

31	**Abstract**

32 The~~is~~ study investigated the effects ~~impact~~ of school~~-based~~ menstrual hygiene
33 management,~~education~~, ~~access to~~ water and sanitation interventions ~~services~~ on
34 ~~menstruating~~ girls' empowerment, health and educational outcomes. ~~attendance and~~
35 ~~academic performance.~~
36 The research employed cross-sectional and experimental designs, allowing for
37 comparisons between girls with and without access to school ~~these~~ interventions. The study
38 aimed to explore whether ~~such~~ access to school menstrual hygiene ~~-based~~
39 ~~services~~management services could foster girls’ hygiene behaviour and empowerment,
40 which influence girls’ health and educational outcomes (class attendance and academic
41 performance). ~~a supportive environment for menstruating girls, enhancing their ability to~~
42 ~~manage menstruation-related needs and reducing absenteeism.~~
43 The results demonstrated a marked improvement in confidence among girls at intervention
44 schools, with 54% feeling comfortable purchasing sanitary products at shopping centres
45 compared to 18% in non-intervention schools. Additionally, girls in intervention schools
46 felt more at ease discussing menstruation with boys, with 21% reporting comfort in these
47 conversations versus 8.9% in non-intervention schools. The presence of hygiene facilities
48 also influenced physical health habits, as 51.1% of girls in intervention schools reported
49 practicing genital hygiene three to four times daily, in contrast to 33.3% in non-intervention
50 schools.
51 Class attendance during menstruation also improved significantly, with 68.3% of girls in
52 intervention schools attending class compared to just 29.8% in non-intervention schools,
53 indicating that better access to water and sanitation services reduces menstruation-related
54 absenteeism.
55 The findings suggest that implementing hygiene education, access to water and sanitation
56 services in schools can substantially empower menstruating girls, enhance their physical,
57 emotional and social well-being and reduce absenteeism. Despite these improvements,
58 further research is warranted to assess additional strategies that might more directly
59 enhance educational performance among menstruating girls.
60 **Keywords:** Girls’ empowerment, health, class attendance, educational performance,
61 school interventions, hygiene education,

62

63

64

65

66

67

###### 68 Authors’ summary

69 Improving girls' education has been a key priority in developing countries, as education
70 equips girls for higher-level positions and decision-making roles. However, traditional
71 attitudes toward education and natural processes, such as menstruation, present significant
72 challenges.
73 Studies have investigated how menstruation influences girls' class attendance and academic
74 performance, yet findings have been inconsistent—some found that menstruation affects
75 attendance while others reported no impact. Additionally, these studies often focused solely
76 on the school environment, despite girls being influenced by both school and broader social
77 factors. As a result, solid evidence on the extent to which menstruation affects class
78 attendance and performance remains lacking.
79 Our study employed a comprehensive set of school interventions to examine the effects of
80 menstruation on girls’ attendance and performance. The interventions included access to
81 sex-segregated toilets, water, sanitary products, designated girls' rooms for resting and
82 changing sanitary pads and hygiene education, along with consideration of external
83 environments, like policy and community.
84 Our findings reveal that comprehensive hygiene and sanitation facilities significantly
85 reduced absenteeism among menstruating girls. Furthermore, the interventions boosted
86 girls' confidence in discussing menstruation with peers, family and teachers, positively
87 influencing their physical, social, and emotional well-being.

88

89

90

91

92

93

94

95

96

97

98

99

100	**1. Introduction**

101 Menstruation is a physiological process involving the monthly shedding of the uterine
102 lining, occurring among pubescent girls and pre-menopausal women who are not pregnant
103 [1, 2]. Menstrual hygiene management (MHM) refers to the hygienic handling of menstrual
104 blood using appropriate sanitary products and hygiene practices, including the use of
105 absorbent products, soap, water, and safe disposal of used materials[1, 3].
106 MHM is an essential component of the human experience; however, discussions around
107 these topics are often deemed sensitive or offensive, particularly in developing nation [4,
108 5]. Interest in MHM began to emerge in the 2000s when private and non-governmental
109 organizations focused on educating pubescent girls primarily within school settings [6].
110 In the past two decades, studies have examined the impact of menstruation on girls' class
111 attendance and the influence of the school environment on their hygiene practices [7, 8, 9].
112 An evaluation conducted by United Nation Children’s Fund (UNICEF), Plan International,
113 Save the Children, and Emory University in the Philippines identified three key
114 determinants of MHM within school environments that can empower girls:- access to
115 water, sanitation and hygiene (WASH) facilities, hygiene education and practical guidance
116 along with access to sanitary products. The evaluation noted that the absence of these
117 determinants could lead to poor MHM practices, confusion, and anxiety related to
118 menstrual leakage, as well as potential teasing from peers [10].
119 Since 2012, UNICEF and the University of Colombia have been hosting annual
120 conferences focused on the theme "WASH in Schools Empowers Girls' Education."
121 Proceedings from these conferences indicate that school-based WASH interventions have
122 positively influenced girls' education and menstrual hygiene practices [11].
123 Empowerment is a means through which individuals and groups increase control over their
124 lives, manifesting as self-confidence, self-respect, knowledge and behaviour changes vital
125 for making decisions regarding health and education [12]. However, to date, no study has
126 quantified the impact of school WASH interventions on the empowerment of menstruating
127 girls.
128 In addition, access to safe drinking water, improved sanitation and hygiene can prevent at
129 least 9.1% of the global disease burden [13] and improvements in health outcomes can lead
130 to enhanced class attendance and academic performance [14, 15, 16].
131 Conversely, the absence of WASH services can significantly affect emotional, social, and
132 physical health components, resulting in confusion, physical discomfort such as genital
133 bruising, and heightened anxiety due to menstrual leakage, which can lead to teasing from
134 peers [10]. The World Health Organization defines health as a state of complete physical,
135 mental, and social well-being, and not merely the absence of diseases [17].
136 The relationship between menstruation and class attendance exhibits inconsistencies across
137 different contexts. Studies conducted in Nepal and Malawi reported that menstruation
138 minimally affects girls' class attendance, attributing more significance to factors such as
139 household responsibilities and student motivation [18, 19]. Conversely, researches from
140 Northern Ethiopia and Kenya demonstrate that improvements in hygiene through school
141 interventions positively influence girls' class attendance [8, 20].
142 Continuous class attendance, along with regular assessments and study time, is positively
143 correlated with educational performance [21]. A study on higher education found that
144 regular attendance reduces the likelihood of poor academic performance [22]. Interestingly,
145 while attendance correlates with lower-performing students, this relationship does not
146 extend to higher-performing students [23].
147 Generally, previous studies on MHM laid the groundwork for further research but lacked
148 comprehensiveness. Evaluations from Philippine-based NGOs, alongside UNICEF and the
149 University of Colombia’s conference reports, were qualitative assessments that did not
150 incorporate quantitative data [10, 11]. Additionally, no other study has captured the
151 empowering potential of school-based WASH interventions, except for qualitative project
152 completion reports.
153 Furthermore, existing studies examining impact of menstrual hygiene management on
154 girls’ health have primarily focused on clinical aspects, reproductive tract infections,
155 overlooking critical social, emotional, and physical health dimensions [24]. Research
156 linking MHM practices to class attendance has also been limited, often concentrating solely
157 on in-school factors and neglecting external influences like policies, community dynamics
158 and peer impacts on hygiene practices [7, 8, 9, 17]. However, both school and outside
159 school environments play essential roles in shaping the hygiene practices of menstruating
160 girl students [25].

161

162 The study, therefore, examined both in school and out-of-school factors influencing girls'
163 hygiene behaviour, empowerment, health ~~and~~ and educational outcomes ~~attendance~~
164 through school interventions that were implemented over six years, with active engagement
165 of ~~from both~~ girls and teachers. ~~Consequently, the study successfully achieved its aim and~~
166 ~~objectives.~~
167 The significance of the study stemmed from the limitations identified in the previous
168 studies on MHM and therefore, it aimed to measure the effects of school menstrual hygiene
169 management, water and sanitation interventions on girls’ self-reliance,, health , class
170 attendance and educational performance.

171

172

173

#### 174 2. Materials and methods

##### 175 2.1 Study ~~Design and~~ setting and description of school

176	**interventions**

177 ~~The study employed cross-sectional and experimental designs and~~ The study was
178 conducted in Lasta District, ~~located in the~~ North Wollo Zone of Amhara Regional State in
179 ~~northern~~ Ethiopia. Amhara Regional State is located in North and North West of the Federal
180 Democratic Republic of Ethiopia.
181 ~~Six primary second-cycle schools were selected within the district: three schools that had~~
182 ~~received WASH interventions and three that had not. All schools were situated in rural~~
183 ~~communities where agriculture is the primary livelihood of local residents.~~

184

##### 185 ~~2.2 Description of the school intervention~~

186 The study district, Last District Education Office, identified 35 schools that had severe
187 WASH service shortages before the beginning of the school intervention (2018). Thus, any
188 non-governmental organization that intended to support these schools had the chance to
189 select any one of these schools, depending on its budget and work force capacity. Plan
190 International Ethiopia, an international non-governmental organization, randomly selected
191 10 Primary 2nd Cycle schools in 2018 for MHM and WASH interventions from 35 schools.

192

193 ~~Plan International Ethiopia, an international non-governmental organization, implemented~~
194 ~~a menstrual hygiene management (MHM), water, sanitation and hygiene (WASH) program~~
195 ~~in 10 primary second cycle schools in the Lasta District from July 2018 to January 2024.~~
196 ~~The intervention aimed to improve menstruating girls' hygiene practices and, in turn,~~
197 ~~support their school attendance.~~

198

199 **Contents of the school intervention**: Key components of the interventions included MHM
200 education, ~~provision of~~ sanitary product provisions, access to drinking water, gender-
201 segregated toilets and access to soaps for hygiene practices. Additionally, the school
202 intervention ~~program established~~ constructed ~~dedicated girls’ clubrooms, or~~ MHM rooms,
203 where girls use them ~~could~~ to take rest when they feel sick, change sanitary pads, prepare
204 sanitary pads~~make reusable pads~~ and educate one ~~each~~ another on ~~menstruation and~~
205 menstrual hygiene management.
206 However, control schools did not have these school facilities and hygiene services because
207 of the absence of similar MHM and WASH interventions. The only support available to
208 control schools was general awareness on MHM, provided by the district's "Women and
209 Children Affairs Office" in line with the national education policy. The control schools did
210 not have MHM education tailored to menstruating girls, sex segregated toilets, MHM
211 rooms, access to water, soaps and sanitary products.
212 When the intervention schools gained access to MHM and WASH services, most other
213 schools, including control schools did not get similar services due to district education
214 office budget limitation and absence of more non-governmental organizations working on
215 WASH service provision. Therefore, lack of access to WASH services by control schools
216 was not because of the study but due to schools’ budget shortage and lack of many WASH
217 actors in the district.
218 The frequency of support for WASH services vary based on service type. Some services,
219 like latrines and MHM rooms, are designed for long-term use, while sanitary pads and
220 soaps have shorter lifespans. Thus, the school intervention provided sanitary pad support
221 twice, together with piece of fabric cloth, which girls used to prepared sanitary pads when
222 they had shortages. Similarly, the intervention provided soaps twice and yet menstruating
223 girls contributed a small monthly amount to purchase soap during shortages.
224 The MHM education continued until all students in the intervention school were reached.
225 After all students took the training, MHM club members conducted monthly meeting to
226 evaluate the progress of MHM in the school and provided advice to pubertal girls in their
227 MHM rooms. In addition, school MHM club members organize MHM education at the
228 beginning of each academic year for new students, who had come from different schools.
229 The duration of the school intervention spanned over six years, from July 2018 to January
230 2024. However, the school intervention activities were operational primarily during school
231 terms and were temporarily interrupted during school breaks, during COVID-19 pandemic
232 and some months lost to conflict in the study district. During the intervention period, school
233 intervention facilitators from Plan International Ethiopia, together with the district Women
234 and Children Affairs Office representative and school teachers, conducted consistent
235 follow-ups to ensure that girl students acquire adequate MHM skills and to fill gaps when
236 girl students encountered.
237 Furthermore, to control the effects of contamination between intervention and control
238 schools, the study used geographical isolation and absence of similar intervention in the
239 control schools as major techniques. The intervention and control schools are located in
240 rural areas where communities are sparsely populated, and schools are situated far apart.
241 Additionally, due to gender-based violence and security concerns, girls in the rural areas
242 do not travel long distances to attend school. Thus, only girls living near schools have the
243 opportunity to pursue their education.
244 This geographical isolation meant there was no opportunity for MHM knowledge transfer
245 from intervention schools to control schoolgirl students. In addition, before the selection of
246 control schools, the research team cross –checked whether there was a deliberate
247 knowledge transfer from intervention to control schools and other WASH interventions in
248 the control schools or nearby.
249 Therefore, to address contamination challenges the study conducted the research among
250 rural schools, verified the absence of WASH interventions in the control schools, and
251 ensured that no deliberate attempts were made to transfer knowledge from intervention to
252 control schools for knowledge sharing.

###### 253 2.2 Study design

254 The study employed cross-sectional design because the school intervention had begun in
255 July 2018, while the study on the school intervention started in April 2023. This made the
256 use of longitudinal design and consequently contrasting baseline data with the final school
257 interventions outcomes impossible. Instead, the study used control schools that did not have
258 similar exposure to compare against the schools that received WASH interventions.
259 However, though random sampling method was used to select girls, they did not have the
260 opportunity to switch between groups. Therefore, the study used quasi-experimental
261 design.

###### 262 2.3 Methods and data collection instruments

263 The study used qualitative and quantitative methods of data collection in order to
264 fill gaps that may come because of using one of the methods and thereby improve
265 the fidelity of findings. In terms of sequence, the qualitative methods were used
266 prior to quantitative methods in order to make any necessary changes on the
267 quantitate methods, if gaps are identified during the use of qualitative tools.
268 However, there were also groups and key informant interviews targeted for the
269 qualitative method alone, like, heads of district offices and community
270 representatives. In the second case, only qualitative methods was used and
271 therefore, no sequence was needed.
272 For qualitative data collection, the study used focus group discussions, key
273 informant interviews and observation for school facility and hygiene services. While
274 for quantitative data collection, questionnaire, health centre data and girls’ first
275 semester/half-year academic performance

###### 276 2.3 Study population

277 The study aimed at determining the effects of school interventions on menstruating girl
278 students. Therefore, menstruating girl students in the sampled intervention and control
279 schools made the study population. However, to evaluate the views of communities, boy
280 students and policy influence on girls’ MHM behaviour, the study included boys, women
281 and heads of district offices for FGDs and KIIs.

###### 282 2.4 Ethical issues

283 **Participants’ informed consent**: A day before questionnaire response, the researcher gave
284 briefing to girls on the purpose of the study. The following day, after girls had sufficient
285 time to discuss with their parents and among peers on their participation, girl students
286 provided their written assent/ signed on the space provided on the first page of the
287 questionnaire, before working on the questionnaires. The signed consent is attached with
288 the questionnaire response, as evidence.
289 **Guardians’ informed consent**: Before the data collection day, study schools, with the
290 support of Parents and Teachers’ Association (PTAs), a body that fosters collaboration
291 between schools and communities on educational matters, invited girls’ parents for a brief
292 morning meeting. At the meeting, a briefing was given to parents on the purpose of the
293 study and then, they were asked whether they would allow their daughters to share their
294 views on the status of their school hygiene services.
295 Therefore, all parents participated at the meeting provided verbal consent and some others,
296 who participated in the focus group discussions (FGDs), provided written consent by
297 signing on the consent form.
298 **Informed written consent:** Moreover, the study obtained a written consent from **Lasta**
299 **District Education Office**’ which monitors, coordinates and manage schools in the district.
300 The full written ethics statement reads as follows:-
301 Date: 23Feb.2023
302 Lasta District Education Office, North Wollo Zone of Amhara Region, understands the relevance of
303 examining the ‘*’Effects of School Menstrual Hygiene, Water and Sanitation Interventions on Girls*
304 *Hygiene Knowledge and Practice, Empowerment, Health and Educational Outcomes’*’ at the Primary 2nd
305 Cycle Schools, found in Lasta District.
306 The Office recognizes that the study will include menstruating girls, boys, teachers and community
307 representatives around the study schools and Heads of WASH Sector Offices in Lasta District. We
308 believe that the results of the study may benefit to Lasta District Education Office in particular and
309 Amhara Regional State in general to improve school WASH interventions.
310 Therefore, Lasta District Education Office has given this ethical clearance letter/permission to conduct
311 the inquiry in the schools found in the district and kindly request study schools and district WASH Sector
312 Offices to support in providing required data to Mr. Fisseha Atale, who is running the study.

313

###### 314 2.5 Sampling method and sample size

315 Stratified sampling was used to group 10 intervention and 25 non-control schools based on their
316 similarity in WASH experience. This was followed by selecting three schools randomly from each
317 group. For the intervention schools, the 10 schools were given codes and the codes were mixed.
318 From the mixed school codes, a person who was not part of the process picked three school codes
319 randomly. This procedure was repeated for 25 control schools.
320 Similarly, to select menstruating girl students, a lottery method was used but it followed multistage
321 sampling: First, all boy students were excluded from the sample, as the focus of the study was on
322 girl students. Second, all non-menstruating girl students were excluded from the sample because
323 the study aimed at evaluating the experience of menstruating girl students.
324 After the exclusion of boys and non-menstruating girls, the study team identified 333 and 293
325 menstruating girl students from intervention and control schools respectively. This made 626-study
326 population. Using, the sample size formula and after inclusion of the probably of non-attendant rate,
327 the sample size was 360. Thus, for the sample size, a separate list of girls was prepared for each
328 of the six schools. In the study population, the contribution of the intervention schools was 53%
329 while the control schools was 46.6%. Based on this contribution, 192 and 168 menstruating girls
330 were allocated to intervention and control schools orderly.
331 The study employed a 95% confidence level and a 5% margin of error, with an expected
332 variance of 0.5. Given the known student population, the research applied finite population
333 formula, detailed as follows [26]:-

334	𝑆 = 𝑍^2^𝑁𝑃(1 − 𝑃)/𝑒^2^(𝑁 − 1) + 𝑍^2^𝑃(1 − 𝑃)

335 Where,
336 S: stands for sample size
337 N: Population size of students
338 P: Population proportion, with expected variance of 0.5
339 Z: Z-score, which is 1.96 in this case;
340 e- Margin of error/degree of accuracy, which is 0.05 (5%);
341 Each school received its sample size/share based on its contribution to the study population. Using
342 the prepared list of menstruating girls, a lottery method was used to identify samples from each
343 school. For a school that had 50 menstruating girl students in the list but its sample share/size 25,
344 the study team prepared 50 draws and mixed them. Among 50 draws/pieces of paper, 25 of them
345 were given codes/numbers from 1-25, while the remaining were zero. Then, based on the order of
346 the list of students, each student was asked to withdraw a code. If the draw reads one of the
347 numbers from 1-25, a girl is part of the final sample but if the draw reads zero, a girl is excluded
348 from the sample. Therefore, in both the school and girl student selection, the study used simple
349 random techniques.
350 Blinding one of the quality controlling techniques used to avoid biases during data collection, data
351 analysis, reporting, etc. from those involved in the research process. Though double blinding was
352 not possible, single blinding was used. Participating girl students were blinded due to long years of
353 program implementation in the school where they had been actively engaged from the start of the
354 school intervention and they had different progress evaluation meetings. Thus, they took their
355 participation in the study process as part of the program implementation not as a research that was
356 different from the program implementation. For data collectors, the researcher provided data
357 collectors’’ training where data collectors bias ’was one of the major components of the training.
358 On the other hand, the study team made the purpose of the study clear that the study was not
359 related to any future WASH opportunity for schools. Girls were asked to just share their practical
360 experience on MHM at their schools.
361 In addition, to minimize the researcher’s bias, the study employed different approaches. It employed
362 mixed method of data collection to improve the accuracy of the research finding; used clinical data
363 (recorded from MHM related treatment) to cross check against field data from girl students; used
364 independent samples T-test to avoid considering minor differences between intervention and
365 control school as significant. Furthermore, when the literature reviews are observed, both negative
366 and positive findings were equally presented; it tried to avoid confirmation bias, by employing socio-
367 ecological perspectives that helped the study to consider different levels of factor analysis
368 (individual, group, community, organization, and policy) , which were not common in the previous
369 study designs.
370 Furthermore, the study used random sampling to determine the sample size and the sample
371 represented 57% of the study population in the six study schools.
372 2.6 Mitigating effects of extraneous variables
373 Confounding variables are factors, other than the school interventions, that can influence
374 both independent and dependent variables. To minimize the effect of confounding factors
375 on girls’ class attendance and performance, the study employed the following strategies:
376 1. **Restriction**
377 The study restricted the population to girls of similar age and educational level to
378 reduce variability caused by potential confounders. The majority of the sampled
379 students were between 13 and 15 years old, female, and at comparable educational
380 level— students in the Primary 2nd Cycle Schools (Grades 7 and 8). These
381 restrictions enabled participants to share similar values for key confounding factors.
382 2. **Matching**
383 To control for potential confounding variables, girls in the intervention schools
384 were matched with girls in the control schools who had similar characteristics.
385 These included physiological factors, level of education, family educational
386 background and income, age, exposure to technology, and school settings (both
387 schools were located in rural areas). By ensuring that confounding factors were
388 consistent between the two groups, any differences identified in the study could
389 more confidently be attributed to the school interventions.
390 3. Transportation and Academic Performance
391 Since both intervention and control schools were in rural settings, students shared
392 similar experiences regarding school travel. Almost all girls walked to school,
393 with no notable differences in access to transportation. Additionally, the study
394 accounted for prior academic performance by using mid-year educational
395 performance data from 2024 for comparison, thereby mitigating the influence of
396 pre-existing academic disparities.
397 4. Teaching Quality and Learning Environment
398 Although individual teacher differences could exist, significant differences in
399 teaching quality were unlikely, as neither intervention nor control schools had
400 access to advanced educational technologies or resources. Both were located in
401 similar rural environments, ensuring comparable learning conditions.
402 5. Cultural and Religious Homogeneity
403 The majority of the girls from both intervention and control schools shared the
404 same ethnic background (98% Amhara) and religious faith (84% Orthodox),
405 which reduced the potential influence of cultural or religious differences on the
406 study outcomes.

###### 407 2.7 Data collection

408 Following the training of data collectors and pilot testing (March 7 & 8, 2024), the data
409 collection team conducted the actual data collection from March 11 to 15, 2024. The data
410 sources for the study included:
411 1. Menstruating girl students’ survey: 360 menstruating girls from all sample schools.
412 2. Focus Group Discussions (FGDs**)**: A total of six FGDs, consisting of:
413 o Two with boy students, one from intervention and one from control schools
414 o Two FGDs with menstruating girl students: one from each school category,
415 o Two	FGDs with menstruating girls’ mothers:-one from each school
416 category(intervention and control);
417 3. Key Informant Interviews (KIIs):
418 o Two interviews with school principals, one from intervention and one from
419 control school.
420 o Two interviews with school MHM club facilitators (female teachers), one
421 from intervention and one from control schools.
422 o Ono interview each from Lasta District Education Office and Women and
423 Children Affairs Office, to understand the implication of policies on girls'
424 menstrual hygiene management practices.
425 4. District Health Centre data: Recorded health data from the district health centre
426 regarding girl students’ menstrual hygiene management related health challenges
427 and diarrhoea prevalence, which may come from lack of, improved water.
428 5. Girls’ academic performance: Menstruating girl students’ first semester average
429 scores from six sample schools.
430 The Menstrual Hygiene Management (MHM) education process for girls began with Plan
431 International Ethiopia training selected girl and boy students, along with teachers, from
432 each target school. These trained students, referred to as peer trainers, were then responsible
433 for educating other girls in their schools who had not participated in the initial training.
434 This peer-led MHM training continued until all girl students in each target school had been
435 reached. Plan International Ethiopia provided training manuals to support the trainers, who
436 ~~also received guidance from the teachers who had completed the initial MHM training.~~
437 WASH experts from Plan International Ethiopia regularly visited the target schools to
438 address emerging challenges faced by girls. For instance, they provided additional sanitary
439 pads when supplies ran low and revitalized girls’ clubs when participation weakened for
440 various reasons. This ongoing support was sustained over the six-year project period,
441 ~~concluding in January 2024.~~
442 In contrast, the non-intervention schools did not receive support from Plan International
443 Ethiopia or any other non-governmental organizations. However, as directed by national
444 education policy, all schools in the country maintain co-curricular activities, including a
445 “Girls' Club” in each school. Occasionally, the government—through the Women and
446 Children Affairs Office—provides training for teachers who oversee these school clubs and
447 activities. While these trainings include some information on menstruation and menstrual
448 hygiene management, their primary focus is on topics like preventing unwanted
449 pregnancies and early marriage. Consequently, girls in the intervention schools experienced
450 ~~a significantly different environment compared to those in the non-intervention schools.~~
451 Thus, the aim of the study was to assess the impact of school-based MHM and WASH
452 interventions on girls’ empowerment, health, class attendance and academic performance
453 ~~by comparing outcomes between girls from intervention and non-intervention schools.~~
454 T~~he study employed a 95% confidence level and a 5% margin of error, with an expected~~
455 variance of 0.5. Given the known student population, the research applied finite population
456 ~~formula, detailed as follows [26]:-~~

457	𝑆 = 𝑍^2^𝑁𝑃(1 − 𝑃)/𝑒^2^(𝑁 − 1) + 𝑍^2^𝑃(1 − 𝑃)

458 ~~Where,~~
459 ~~S: stands for sample size~~
460 ~~N: Population size of students~~
461 ~~P: Population proportion, with expected variance of 0.5~~
462 ~~Z: Z-score, which is 1.96 in this case;~~
463 ~~e- Margin of error/degree of accuracy, which is 0.05 (5%);~~
464 The total student population across the six schools, including boys, was 1,214. Excluding
465 boys and non-menstruating girls, the study population consisted of 626 pubescent girls.
466 Initially, a sample size of 240 girl students was calculated. However, data collection
467 coincided with the local wedding season when many girls were occupied with family
468 obligations and an uncertain security situation. To reduce the risk of absenteeism during
469 questionnaire administration, the sample size was increased by 19%, adding 120 girls,
470 ~~resulting in a final sample of 360 menstruating girls.~~
471 The total sample size was allocated to intervention and non-intervention schools
472 proportionally, based on each group’s share of the total population: 190 girls from
473 intervention schools and 170 girls from non-intervention schools. Luckily, all participants
474 ~~were present for the questionnaire, yielding a 100% response rate.~~

##### 475 ~~2.3 Informed consent~~

476 To ensure ethical compliance, the study secured informed consent from all participants in
477 addition to obtaining ethical clearance from the district education office. Prior to requesting
478 consent, the researcher provided participants with detailed information about the study,
479 ~~including:~~
480 ~~The researchers' identities, contact information and phone numbers.~~
481 ~~The purpose and nature of the study.~~
482 ~~The potential risks and benefits of participation.~~
483 ~~The rationale for selecting the study schools and menstruating girls.~~
484 Assurance of anonymity and respect for their autonomy during the data collection process.
485 Participants were explicitly informed that they had the right to withdraw from the study at
486 any time. This information was conveyed in a clear manner to ensure participants fully
487 ~~understood the objectives of study and their rights.~~
488 An interactive question-and-answer session followed the information briefing. During this
489 session, researchers addressed participants' concerns and queries. This discussion was held
490 before the data collection day—Friday, 8 March 2024—to provide participants ample time
491 to decide on their participation and to minimize delays or fatigue on the actual data
492 ~~collection day.~~
493 Subsequently, menstruating girls were asked whether they would voluntarily participate in
494 the study. All participants provided verbal consent. As part of the consent process,
495 participants were also informed that they would be required to provide a signature during
496 questionnaire administration. Thus, a designated signature space was included on the first
497 page of the questionnaire. Accordingly, the informed consent process involved two steps:
498 ~~initial verbal consent followed by written consent.~~
499 The study used cross-sectional design and therefore the data collection time was conducted
500 from Monday, 11 March 2024, to Friday, 15 March 2024. These activities included key
501 informant interviews, focus group discussions, observations, and questionnaire
502 ~~administration.~~

##### 503 2.8~~4~~ Statistical methods used

504 Data analysis preparation began with data cleaning, coding of both qualitative and
505 quantitative data, and entering the data into a spreadsheet. Qualitative data was analysed
506 using thematic analysis, while quantitative data was processed with SPSS software.
507 Descriptive statistics, such as frequencies and percentages, were used to summarize
508 respondents' characteristics and responses to each question. To determine statistical
509 significance, the independent samples t-test was extensively applied. Finally, results from
510 both qualitative and quantitative analyses were integrated to determine research
511 hypotheses.

512

513

514

515

516

517	**3. Results**

##### 518 3.1 Sample characteristics

519 The majority of menstruating girl students were aged 13 to 15 years (68.9%), followed by
520 those aged 16 to 18 years (15.6%) and 10 to 12 years (15.3%). Among the surveyed girls,
521 97.8% identified as Amhara ethnic, while 1.1% identified as Agew and 0.8% as Tigre. In
522 terms of religious affiliation, 83.9% identified as Orthodox, 9.4% as Protestant, and 6.1%
523 as Muslim.
524 Regarding parental education, ~~Parental education levels were generally low, with a higher~~
525 ~~percentage of fathers~~ 66.2% of girls’ fathers was able to read and write against ~~compared~~
526 51.7% of girls’ mothers ~~to mothers (66.2% versus 51.7%). Thus, the illiteracy rate among~~
527 ~~mothers was higher than that of fathers (47.8% compared to 32.8%)~~. Economically,81.7%
528 of girls’ parents made their living from agriculture ~~was the primary source of income for~~
529 ~~parents, accounting for 81.7% of their livelihoods,~~ while people engaged in business was
530 ~~activities were minimal at just~~ only 8.3% (Table 1).
531 **Table 1: Demographic characteristic of the sample**

| N=360 | Characteristics | Frequency | Percent |
| --- | --- | --- | --- |
| ~~Gender~~ | Female | 360 | 100 |
| Age | 10-12 | 55 | 15.3 |
|  | 13-15 | 248 | 68.9 |
|  | 16-18 | 56 | 15.6 |
|  | Above 19 | 1 | 0.3 |
| Religion | Orthodox | 302 | 83.9 |
|  | Islam | 22 | 6.1 |
|  | Protestant | 34 | 9.4 |
|  | Other | 2 | 0.6 |
| Ethnic group | Amhara | 353 | 98.1 |
|  | Agew | 4 | 1.1 |
|  | Tigre | 3 | 0.8 |
| Parents' occupation | Farmer | 294 | 81.7 |
|  | Merchant | 30 | 8.3 |
|  | Office worker | 16 | 4.4 |
|  | Other | 20 | 5.6 |
| Mothers' level of  education | Abel to read and write | 144 | 40 |
|  | Grade 1-6 | 28 | 7.8 |

|  | Grade 7-12 | 14 | 3.9 |
| --- | --- | --- | --- |
|  | Not able to read and write | 172 | 47.8 |
|  | Other | 2 | 0.6 |
| Fathers' level of education | Abel to read and write | 183 | 50.8 |
|  | Grade 1-6 | 43 | 11.9 |
|  | Grade 7-12 | 14 | 3.9 |
|  | Not able to read and write | 118 | 32.8 |
|  | Other | 2 | 0.6 |

532 • The study subjects were only menstruating girl students and so the gender representation
533 is female only.

534

##### 535 3.2 Empowering girls through water, sanitation and

536	**hygiene interventions**

###### 537 3.2.1 Qualitative results

538 Girls from intervention schools reported having open discussions about menstruation and
539 menstrual hygiene management with their mothers, sisters, boys, and teachers. They felt
540 comfortable asking for permission to take breaks during class if they experienced
541 menstruation. A teacher from intervention school and who was supporting the ’’Girls’
542 Club’’ said, “Girls are not afraid of asking for breaks when menstruation occurs during
543 classes.”

544

545 In contrast, girl students from non-intervention schools indicated that their discussions
546 were limited primarily to female teachers. They felt uncomfortable talking about
547 menstruation with boys, who often joked about the topic. A teacher from a non-
548 intervention school remarked, “Girls lack adequate knowledge about menstruation and
549 MHM and they don’t have courage to discuss these topics with teachers and peers.”

550

###### 551 3.2.2 Quantitative results

552 Girls from intervention schools demonstrated greater confidence when purchasing sanitary
553 products compared to their peers in non-intervention schools. For example, 54% of girls
554 from intervention schools reported feeling no fear while buying sanitary products, whereas
555 only 18% of the girls from non-intervention schools reported similar confidence.
556 Conversely, 41% of girls in non-intervention schools admitted to feeling fear when
557 purchasing sanitary pads, compared to only 18% of girls from intervention schools who
558 experienced similar feelings (Fig. 1).

559

560	Overall, 58% of girls in non-intervention schools reported feeling shy or fearful when

561	buying sanitary products, while only 31% of girls from intervention schools expressed

562	these concerns, indicating that girls from non-intervention schools were nearly twice as

563	likely to experience fear compared to their peers in intervention schools.

564

565

Intervention schools	Non-intervention schools

Frequency	Percent

buy	shy

from shops

shy

my face

I cover I feel no I do not I feel Total

I feel I do not I cover Total

shy	buy my face from

shops

I feel

no shy

0

54%	18%	16%	13% 100% 17%	18%	25%	41% 100%

34	36

30

50

50

37

43

83

100

127

150

**Girls' experience while buying sanitary products at shops**

237

250

203

200

**Frequency**

566	**Figure 1**: **Comparing girls’ confidence while purchasing sanitary products**

567

568 Quantitative results indicated significant differences in social interactions between girls in
569 the two school environments regarding their relationships with peers and teachers. For
570 instance, 21% of girls from intervention schools reported being able to discuss menstruation
571 and menstrual hygiene management with boys, compared to only 8.9% of girls from non-
572 intervention schools.
573 Additionally, 22% of girls from intervention/treatment schools felt that menstrual hygiene
574 management (MHM) education enabled them to discuss menstruation with parents and
575 others, while only 9% of girls from non-intervention schools felt the same. Furthermore,
576 33% of girls in non-intervention schools reported not receiving MHM education while
577 only to just 2% of those in intervention schools (Figure 2).

Frequency

578

Control schools

Treatmetn schools

1^9^7	1^8^6

1^9^8

19

37

22

44

33

64

22

63

22

61

21

59

26	1^4^0

196

29

82

281

100%

Percent

Frequency

100%

**Impacts of school intervention on girl students**

350

300

250

200

150

100

50

0

No MHM education at school

No change at all

Helped me discuss with boys on MHM

Helped me discuss with parnets & others on MHM

Helped me improve communication skills

I got courage to ask questions

Total

Total

No MHM education at school

I got courage to ask questions

Helped me improve communication skills

Helped me discuss with parnets and others on MHM

Helped me discuss with boys on MHM

No change at all

579	**Figure 2: Effects of school interventions on girls’ social relations and communications**

580	*MHM stands for menstrual hygiene management education at schools. Only 2% of the girls from intervention*

581	*schools reported absence of MHM education in contrast to 33% of the girls from non-intervention schools.*

582

583	When the differences in girls’ courage to ask questions, improve communication skills and to discuss with

584	boys and parents on MHM were evaluated using the independent samples t- test, differences were

585	significant where the P-values lie between 0.001 and 0.022. Thus, P<α; 0.001<0.05 and 0.023<0.05

586	(table2).

587

588

589

590

591

592

593

594

595	**Table 2: T-test results: Impacts of school intervention on girls’ communication and social**

596	**relations**

| **Independent Samples Test** | | | | | | | | | | |
| --- | --- | --- | --- | --- | --- | --- | --- | --- | --- | --- |
|  |  | *Levene's Test for Equality of Variances* | | *t-test for Equality of Means* | | | | | | |
|  |  | *F* | *Sig.* | *t* | *df* | *Sig. (2-*  *tailed*  *)* | *Mean Differ ence* | *Std. Error Differ*  *ence* | *95% Confidence Interval of the Difference* | |
|  |  |  |  |  |  |  |  |  | *Lower* | *Uppe*  *r* |
| I got courage to ask  questions in class | Equal  variances assumed | 19.349 | 0.000 | 2.302 | 182 | 0.022 | 0.161 | 0.070 | 0.023 | 0.299 |
|  | Equal variances not  assumed |  |  | 2.319 | 181.924 | 0.022 | 0.161 | 0.069 | 0.024 | 0.298 |
| It Helped me improve communicati on skills | Equal variances  assumed | 10.569 | 0.001 | 1.599 | 182 | 0.112 | 0.106 | 0.066 | -0.025 | 0.236 |
|  | Equal variances not  assumed |  |  | 1.612 | 181.983 | 0.109 | 0.106 | 0.066 | -0.024 | 0.235 |
| It helped me discuss with boys on MHM | Equal  variances assumed | 14.256 | 0.000 | 2.543 | 182 | 0.012 | 0.283 | 0.111 | 0.063 | 0.503 |
|  | Equal  variances not assumed |  |  | 2.684 | 117.885 | 0.008 | 0.283 | 0.105 | 0.074 | 0.492 |
| It helped me discuss with parents and others on MHM | Equal variances  assumed | 63.424 | 0.000 | 3.573 | 182 | 0.000 | 0.212 | 0.059 | 0.095 | 0.329 |
|  | Equal  variances not assumed |  |  | 3.667 | 169.380 | 0.000 | 0.212 | 0.058 | 0.098 | 0.326 |
| No change at all | Equal variances  assumed | 4.993 | 0.027 | - 1.107 | 182 | 0.270 | - 0.042 | 0.038 | -0.117 | 0.033 |
|  | Equal  variances not assumed |  |  | - 1.088 | 157.158 | 0.278 | - 0.042 | 0.039 | -0.118 | 0.034 |
| No MHM  education at school | Equal  variances assumed | 357.730 | 0.000 | - 6.501 | 182 | 0.000 | - 0.341 | 0.053 | -0.445 | - 0.238 |
|  | Equal variances not  assumed |  |  | - 6.179 | 103.847 | 0.000 | - 0.341 | 0.055 | -0.451 | - 0.232 |

597

598 3.3 Improving girls’ health through school interventions
599 Clinical data collected from Shumshiha Health Centre, which serves study district
600 communities including girl students from both intervention and non-intervention schools
601 revealed that from September 2022 to February 2024, 41 women visited the centre due to
602 illnesses related to poor menstrual hygiene practices. Among these patients, 14 (34%)
603 were menstruating students. The illnesses were primarily caused by excessive use of
604 sanitary pads and a lack of awareness regarding menstrual hygiene management, as the
605 clinical evidence indicated. This data highlights that girls were at risk for genital bruising
606 due to the overuse of sanitary pads/poor hygiene practices and low level of menstrual
607 hygiene knowledge.
608 However, the school-based menstrual hygiene management (MHM) and WASH
609 interventions significantly reduced girls' risk of genital bruising by enhancing their
610 hygiene knowledge and practices (Fig. 3). For example, 43.8% of girls in the intervention
611 schools reported practicing genital hygiene three times a day during menstruation,
612 compared to only 19% in non-intervention schools. Additionally, 51.1% of girls from
613 intervention schools practiced genital cleaning three to four times per day against 33.3%
614 of girls from non-intervention schools.

**Girls’ daily hygiene practice during menstruation**

100	44	26	23	7.3	6	14	19	20	41	100

192	84	50	44	14	10	24	32	34	68	168

Frequency	Percent

| Total | Three Twice One times a day time a per day day | More than four times | Do not wash | More Three More Twice than times than a day four four  times times | Total |
| --- | --- | --- | --- | --- | --- |
|  | Intervention school |  |  | non-intervention school |  |

615

616 **Figure 3: Comparing daily hygiene practices of girls during menstruation**
617 In terms of emotional and social aspects of girls’ health, 58% of female students from non-
618 intervention schools reported feeling shy or fearful when buying sanitary products,
619 compared to 31% of those from intervention schools (Fig.1). In addition, 21% of girls from
620 intervention schools reported feeling comfortable discussing menstruation and menstrual
621 hygiene management with boys, compared to only 8.9% of girls from control schools.
622 Furthermore, 22% of girls from intervention schools felt that menstrual hygiene
623 management (MHM) education helped them talk about menstruation with their parents and
624 others, while only 9% of girls from non-intervention schools shared this belief (Fig. 2).
625 Data from the same health centre, Shumshiha Health Centre, further indicated that
626 waterborne diseases were a significant challenge for the community, including girl
627 students. During the same reporting period, the health centre recorded 125 visits by women
628 due to waterborne diseases, with 51 (41%) of these visits made by girl students.
629 The most common waterborne illness identified was diarrhoea, primarily caused by a lack
630 of access to improved water and water contamination, as noted by the health centre. This
631 finding aligns with data from the district education office, which revealed that 67% of
632 schools in the district lacked access to improved water sources.
633 The school WASH intervention, however, significantly improved girls’ access to safe
634 drinking water in the intervention schools. Observations of school facilities and feedback
635 from focus group discussions revealed that, among the three sampled intervention schools,
636 two had access to improved water, while the third school did not have a functioning water
637 system at the time of data collection. In contrast, none of the sampled non-intervention
638 schools had access to improved water.
639 When water was available at schools, girls reported using it to support safe hygiene
640 practices. For example, 41% of girls from intervention schools used school water for
641 washing sanitary pads and 17% used it for genital cleaning. In comparison, only 24% and
642 8% of girls from non-intervention schools reported using water for sanitary pad washing
643 and genital cleaning respectively (Fig. 4).

**Benefits of school water to menstruating girls**

**Total**

**No water at school Cleaning rooms**

**Drinking and hand washing**

**Genital cleaning Menstrual pad washing**

**Total No water at school Cleaning rooms**

**Drinking and hand washing**

**Genital cleaning Menstrual pad washing**

**242**

**93**

**40**

**31**

**19**

**59**

**346**

**8**

**58**

**80**

**59**

**141**

**100%**

**38%**

**17%**

**13%**

**8%**

**24%**

**100%**

**2%**

**17%**

**23%**

**17%**

**41%**

Frequency	Percent

**Non-intervention schools**

644

**Intervention schools**

645	**Figure 4: Comparing the effects of school interventions on the use of water by girl students**

646	The difference in the use of school water, when available, for hygiene purpose between

647	two groups of girls was significant when tested through the independent t-test, indicating

648	that all the P-values, except one, had less than 0.05.Thus, P<α; 0.001<0.05(Table 3).

649

650

651

652

653

654	**Table 3: T-test results: Multiple benefits of school water to menstruating girl**

655	**students**

| **Independent Samples Test** | | | | | | | | | | |
| --- | --- | --- | --- | --- | --- | --- | --- | --- | --- | --- |
|  |  | Levene's Test for Equality of  Variances | | **t-test for Equality of Means** | | | | | | |
|  |  | F | Sig. | t | df | Sig. (2-  tailed) | Mean Difference | Std. Error Difference | 95%  Confidence Interval of the  Difference | |
|  |  |  |  |  |  |  |  |  | Lower | Upper |
| Menstrual pad washing | Equal variances  assumed | 5.779 | 0.017 | 5.668 | 182 | 0.001 | 0.386 | 0.068 | 0.252 | 0.52 |
|  | Equal variances  not assumed |  |  | 5.639 | 174.45 | 0.001 | 0.386 | 0.068 | 0.251 | 0.521 |
| Genital cleaning | Equal variances  assumed | 48.105 | 0 | 3.183 | 182 | 0.002 | 0.19 | 0.06 | 0.072 | 0.308 |
|  | Equal variances  not assumed |  |  | 3.256 | 173.37 | 0.001 | 0.19 | 0.058 | 0.075 | 0.305 |
| Drinking and hand washing | Equal variances  assumed | 48.809 | 0 | 3.493 | 182 | 0.001 | 0.232 | 0.067 | 0.101 | 0.364 |
|  | Equal variances  not assumed |  |  | 3.547 | 180.06 | 0.001 | 0.232 | 0.065 | 0.103 | 0.362 |
| Cleaning rooms | Equal variances  assumed | 5.106 | 0.025 | 1.117 | 182 | 0.266 | 0.074 | 0.066 | -  0.056 | 0.204 |
|  | Equal variances  not assumed |  |  | 1.123 | 181.63 | 0.263 | 0.074 | 0.065 | -  0.056 | 0.203 |
| No water at school | Equal variances  assumed | 407.451 | 0 | -9.438 | 182 | 0.001 | -0.517 | 0.055 | -  0.625 | -  0.409 |
|  | Equal variances  not assumed |  |  | -8.999 | 108.45 | 0.001 | -0.517 | 0.057 | -  0.631 | -  0.403 |

656

##### 657 3.4. Effects of school interventions on girls’ class attendance

###### 658 3.4.1 Qualitative results

659 Girl students, their mothers and teachers unanimously agreed that the primary causes of
660 girls’ class absenteeism were family responsibilities, such as caring for sick and
661 participating in ceremonies, as well as personal illness. Neither mothers nor girl students
662 cited menstruation as a reason for absenteeism.
663 Teachers from intervention schools also reported that no pubescent girl students were
664 absent due to menstruation because of the availability of hygiene management services at
665 schools. A female teacher supporting girls’ MHM club at the intervention school remarked,
666 " Menstruating girl students do not miss classes during menstruation because they receive
667 better hygiene services at school than at their homes."
668 However, teachers from non-intervention schools noted that girl students often-missed classes due
669 to lack of hygiene-supportive services at the school and yet girls were reluctant to disclose their
670 reasons for absence out of fear. Thus, the message was that the availability of hygiene services at
671 schools enhanced girls’ school attendance and vice-versa.

672

###### 673 3.4.2 Quantitative results

674 The quantitative results further supported the qualitative evidence regarding girls’ class
675 attendance. When asked about their school attendance during menstruation, 68.3% of girls
676 from intervention schools reported that they often attended school, compared to 29.8% of
677 girls from non-intervention schools who responded similarly (fig5).
678 For girls from non-intervention schools, the primary causes of class absenteeism, based
679 on the provided options, were lack of sanitary pads (31%) and lack of water at schools
680 (17%). Additionally, 11% cited menstrual pain and discomfort with toilets. In contrast, for
681 girls from intervention schools, the major factors contributing to class absence included
682 menstrual pain (9%), lack of water and sanitary pad (7.8%) and lack of comfortable toilets
683 (6.4%). The result shows, contrasting causes of girls’ class absenteeism attributed to the
684 school hygiene and WASH interventions (fig.5). Approximately 70.2% of girls from non-
685 intervention schools reported being absent from classes due to menstrual hygiene
686 management (MHM) related challenges, compared to 31.7% of girls from intervention
687 schools.

***Frequency	Percent***

***200	250	300***

***150***

***Frequency***

***0	50	100***

***No,I don't go to school due to menstrual related… 21 10%***

***No,I don't go to school due to poor toilets	146% No, I don't go to school due to lack of water	178%***

***No I do't go to school due to lack of sanitray pads	178%***

***Yes, I often go to school	149	68%***

***100%***

***31%***

***30%***

***17%***

***42***

***76***

***73***

***218***

***27 11%***

***No,I don't go to school due to poor toilets***

***No, I don't go to school due to due lack of water No I do't go to school due to lack of pads***

***Yes, I often go to school***

***Total***

***100.0%***

***Girls class attendance during menstruation***

***Total	245***

***No,I don't go to school due to menstrual related… 27 11%***

***Non-intervention schools***

688

***Intervention schools***

689 **Figure5: Class attendance comparison between intervention and non-intervention**
690 **menstruating girls during menstruation**
691 When the difference in class absenteeism between girls in intervention and non-
692 intervention schools was evaluated using the independent samples t-test, all
693 characteristics, except one, had p-values between 0.001 and 0.004 which are less than 0.05
694 (Table 4). Thus, with p < α (0.001 < 0.05; 0.004<0.05), there was a significant difference
695 in class absenteeism during menstruation between the two groups of girl students.
696 **Table 4: T-test results: Impacts of school intervention on girls’ class attendance**

| **Independent Samples Test** | | | | | | | | | | |
| --- | --- | --- | --- | --- | --- | --- | --- | --- | --- | --- |
|  |  | **Levene's Test for Equality of**  **Variances** | | **t-test for Equality of Means** | | | | | | |
| How often do you go to school during menstruation? |  | F | Sig. | t | df | Sig. (2-  tailed) | Mean Difference | Std. Error Difference | 95% Confidence  Interval of the Difference | |
|  |  |  |  |  |  |  |  |  | Lower | Upper |
| Yes ,I often go to school | Equal variances  assumed | 30.100 | 0.000 | 5.104 | 182 | 0.000 | 0.345 | 0.068 | 0.212 | 0.479 |
|  | Equal variances not  assumed |  |  | 5.048 | 167.030 | 0.000 | 0.345 | 0.068 | 0.210 | 0.480 |
| No, I do not go due to lack of pads | Equal variances  assumed | 160.112 | 0.000 | -6.081 | 182 | 0.000 | -0.362 | 0.059 | -0.479 | -0.244 |
|  | Equal variances not  assumed |  |  | -5.886 | 132.432 | 0.000 | -0.362 | 0.061 | -0.483 | -0.240 |
| No, I do not go due to lack of water | Equal  variances assumed | 40.802 | 0.000 | -3.022 | 182 | 0.003 | -0.164 | 0.054 | -0.271 | -0.057 |
|  | Equal variances not  assumed |  |  | -2.945 | 144.171 | 0.004 | -0.164 | 0.056 | -0.274 | -0.054 |
| No, I do not go to due to unclean toilets | Equal  variances assumed | 20.063 | 0.000 | -2.164 | 182 | 0.032 | -0.103 | 0.048 | -0.197 | -0.009 |
|  | Equal variances not  assumed |  |  | -2.112 | 146.544 | 0.036 | -0.103 | 0.049 | -0.199 | -0.007 |
| No, I do not go to school, due to menstrual related pain | Equal variances  assumed | 3.996 | 0.047 | -0.996 | 182 | 0.321 | -0.051 | 0.051 | -0.151 | 0.050 |
|  | Equal variances not  assumed |  |  | -0.985 | 168.278 | 0.326 | -0.051 | 0.051 | -0.152 | 0.051 |

697

698 • The t-test results show that menstrual pain was the only factor found to be non-significant,
699 suggesting that class absenteeism related to menstrual pain is unaffected by current school
700 interventions and may require additional measures.
701 Considering both menstrual and non-menstrual period of girls’ class attendance, for girls
702 in non-intervention schools, the top three factors contributing to class absenteeism were
703 family responsibilities (38%), lack of sanitary pads (26%) and menstrual pain and other
704 illnesses (18%). In contrast, for girls in intervention schools, the first three causes of class
705 absence were menstrual pain (30%), family responsibilities (30%), and other illnesses
706 (21%). Notably, girls in intervention schools did not mention lack of sanitary pads, a
707 significant factor for girls in non-intervention schools (Fig. 6).

21%

40

Frequency	Percent

168

166

63

26%

43

18%	18%

30

30

Intervention schools

Non-intervention schools

58

59

38%

30%

31%

157

192

100% 99%

82%

100%

**1**

250

200

150

100

50

0

**M a j o r c a u s e s o f p u b s c e n t g i r l s c l a s s a b s e n t e e i s m**

**Frequency**

Pain related to menses

Family work

Other sickness

Family work

Lack of sanitary pad

Pain related to menses

Other sickness

708

Toal sample studens in the category

Total students selected ranks

Toal sample studens in the category

Total students selected ranks

| 709 | **Figure 6: Comparing top causes of girls’ class absenteeism** |
| --- | --- |
| 710 | - Most students ,82%, in the intervention schools reported that menstrual related pain is the |
| 711 | major cause of class absence while 99% of the girls from non-intervention schools |
| 712 | indicated that family work was number one reason for their class absenteeism. |
| 713 |  |
| 714 | **3.5 Impacts of class attendance on girls’ performance** |
| 715 | **3.5.1 Girls’ study time at home** |
| 716 | The study showed that 77.6% of girls from intervention schools had sufficient time to |
| 717 | study at home, compared to only 41.1% of girls from non-intervention schools. |
| 718 | Additionally, 53% of girls in non-intervention schools reported lack of adequate study |
| 719 | time at home due to family responsibilities, whereas only 10.9% of girls from intervention |
| 720 | schools cited the same issue (Figure.7). Thus, the result indicates that girls from |
| 721 | intervention schools had more time for studying at home compared to those from non- |
| 722 | intervention schools. |

723

Frequency

734

724

725

726

727

728

729

730

731

732

733

53

11	6

10

5

2 1

8 5

41

I have I have I often I have Total I have I often I have I have Total

enough limited study at enough	enough study at enough limited time to time to library time to	time to library time to time to

study study

due to family work

study

but no interest

study

but no interest

study study

due to family work

Intervention schools	Non-initervetnion schools

Frequency	Percent

12

21

50

25

0

78

100

69

100

89

125

100

75

149

168

175

150

**Girls’ study time at home**

200	192

735 **Figure 7: Comparing menstruating girls’ study time at home:-intervention against non-intervention**
736 **schools**
737 The P-value for the difference in study time was 0.001, which is less than the significance
738 level of 0.05 (P < α; 0.001 < 0.05). This indicates a statistically significant difference in
739 study time between girls in the intervention and non-intervention schools (Table 5).
740 **Table 5: T-test results: Girls study time at home**

Independent Samples Test

|  |  | Levene's Test  for Equality of Variances | | t-test for Equality of Means | | | | | |  |
| --- | --- | --- | --- | --- | --- | --- | --- | --- | --- | --- |
|  |  | F | Sig. | t | df | Sig. (2- | Mean | Std. | 95% Confidence | |
|  |  |  |  |  |  | tailed) | Differe | Error | Interval | |
|  |  |  |  |  |  |  | nce | Differe |  | |
| Do you |  |  |  |  |  |  |  | nce |  | |
| have |  |  |  |  |  |  |  |  |  |  |
|  |  |  |  |  |  |  |  |  | Lower | Upper |
| enough time to study at home? |  |  |  |  |  |  |  |  |  |  |
|  | Equal variances assumed | 0.846 | 0.358 | -3.522 | 358 | 0.011 | -0.295 | 0.084 | -0.460 | -0.130 |
|  | Equal |  |  | -3.560 | 357.740 | 0.010 | -0.295 | 0.083 | -0.459 | -0.132 |
|  | variances |  |  |  |  |  |  |  |  |  |
|  | not assumed |  |  |  |  |  |  |  |  |  |

741

742

###### 743 3.5.2 Girls’ educational performance

744 To assess the impact of school interventions on girls' academic performance, their first-
745 semester average scores were collected and compared. Among the 333 girls (menstruating
746 and non-menstruating girls) in the intervention schools, 2% scored "excellent," 4% scored
747 "very good," 14% scored "good," and 29% scored "fair." In comparison, among the 293
748 girls in non-intervention schools, 3% scored "excellent," 9% scored "very good," 16%
749 scored "good," and 25% scored "fair" (Figure 7). These ratings are based on the standards
750 set by the Ethiopian Ministry of Education for primary and secondary school students.
751 In summary, 12% of girls from control schools achieved "very good" and "excellent"
752 scores, compared to only 6% of girls in intervention schools, which is less than half the
753 percentage in control schools. However, for scores rated as "good" and "fair," girls from
754 intervention schools led with 43% compared to 41% in control schools.

Frequencey	Percent

Total

Control school

Total

**Average score**

Treatment school

91-100 81-90 71-80 61-70 61-100 91-100 81-90 71-80 61-70 61-100

25

16

9

8 3

14

12 4

5 2

26

29

40

20

0

46

48

74

100

100

98

154

163

180

160

140

120

100

80

60

**Girl students' first semester average score**

**Number of students**

755

756	**Figure 8: Comparing menstruating girls’ first semester educational performance**

757	Frequencies indicated in blue represent the number of students, while those in red show the

758	percentage of girls within each rating scale who scored at that level. This includes

759	menstruating girls, further divided into those who scored above 60 and those below 61. 760

761

762

763

764

765

766

767	**4. Discussion**

768	Access to adequate hygiene knowledge and management skills was essential for building

769	confidence among girl students. Accordingly, one of the study's hypotheses proposed was

770	that "school MHM and WASH interventions empower girl students."

771

772	Qualitative results from focus group discussions with mothers, girl students, and teachers

773	indicated that girls from intervention schools felt more confident in discussing

774	menstruation and hygiene management with peers, teachers, and mothers compared to

775	girls from non-intervention schools.

776

777 Quantitative findings revealed that 54% of girls in intervention schools felt confident buying
778 sanitary products compared to 18% in non-intervention schools; while 41% of non-intervention
779 school girls felt fear versus 18% in intervention schools (Fig. 1). Similarly, 21% and 22% of
780 intervention school girls felt confident discussing menstruation with boys and parents respectively
781 compared to 8.9% and 9%of the girls from in non-intervention schools in the same order (Fig. 2).
782 The difference between intervention and non-intervention schools girls in self - confidence
783 was statistically significant when tested using the independent samples t-test, showing a
784 P-value of 0.001, which is less than 0.05. Thus, as P < α (0.001 < 0.05), the null hypothesis
785 was rejected, and the alternative hypothesis was accepted. Accordingly, there was a
786 significant difference in the empowerment of girl students between intervention and non-
787 intervention schools (Table 2).

788

789 The observed difference in girls' empowerment was attributed to the impact of school
790 hygiene and WASH interventions. The interventions enhanced girls' hygiene knowledge
791 through MHM education and provided opportunities to discuss menstruation with boys,
792 teachers, peers and parents. As a result, girls in intervention schools demonstrated
793 increased confidence, reflected in their social interactions with peers, teachers, and
794 parents, and in their emotional control when purchasing sanitary products at shops.

795

796 The findings align with an assessment report from UNICEF, Plan International, Save the
797 Children, and Emory University, based in the Philippines, which identified WASH
798 facilities, knowledge, practical guidance, and access to sanitary products as essential
799 services that empower girls [10]. These institutions reported that the lack of these services
800 lead to unsafe hygiene practices, confusion, anxiety about staining clothes with blood and
801 teasing by boys.
802 The consecutive proceedings of the United Nation Children’s fund (UNICEF) and the
803 University of Columbia on WASH over the past ten years have also shown that school
804 WASH interventions are empowering girls' education [11]. Furthermore, studies focusing
805 on MHM and class attendance in Niger and Uganda [5, 27] emphasized the importance of
806 school infrastructure, MHM knowledge and access to WASH services to effect profound
807 changes among adolescent girls in menstrual hygiene knowledge and practices.
808 The findings of this study on girls’ empowerment through school interventions, which
809 included a comprehensive package of hygiene services in the school environment, appear
810 to be unique. The findings may contribute to filling the knowledge gap identified in
811 previous studies, which primarily focused mainly on the effects of menstruation on girls’
812 class attendance.
813 In addition, women’s empowerment has been a prominent global issue, particularly in
814 developing countries. The findings of this study suggest that school hygiene and WASH
815 interventions can serve as effective approach to empower girl students, who represent
816 future leaders. Finally, the finding may inform local government budget decisions
817 regarding school interventions and encourage non-governmental organizations to enhance
818 efforts in school hygiene and WASH interventions.
819 **Enhancing Girls’ Health through School Interventions:** Lack of school-based MHM
820 education and WASH interventions leads to unsafe hygiene practices, confusion, and
821 anxiety among girl students, negatively affecting their physical, emotional, and social
822 health [10]. Accordingly, the hypothesis for this section of the study was that "school MHM
823 and WASH interventions positively impact the health of girl students."
824 Based on the study district health centre data from September 2022 to February 2024
825 regarding menstrual hygiene and waterborne diseases, overuse of sanitary products and
826 limited knowledge of menstruation and hygiene management were associated with genital
827 bruising and menstrual hygiene-related discomfort among women and girl students. During
828 this period, 34% of health centre visitors were girl students.
829 However, the school MHM and WASH interventions improved girls’ knowledge and
830 hygiene practices, positively influencing their health. For example, 43.8% of girls in
831 intervention schools practiced genital hygiene three times daily during menstruation,
832 compared to only 19% of girls in control/non-intervention schools. Additionally, 51.1% of
833 girls in intervention schools practiced genital cleaning three to four times daily, while only
834 33.3% of girls in non-intervention schools did so (Fig. 3).
835 In terms of emotional health, 58% of female students in non-intervention schools reported
836 feeling shy or fearful when purchasing sanitary products, compared to 31% in intervention
837 schools. This finding suggests that the school intervention reduced fears among girls in
838 intervention schools by nearly half compared to their peers in non-intervention schools
839 (Fig. 1).
840 Furthermore, the intervention enhanced girls' communication and social interactions with
841 peers and teachers (social health). For instance, 21% of girls in treatment/intervention
842 schools reported being comfortable discussing menstruation and menstrual hygiene
843 management with boys, compared to only 8.9% of girls in non-intervention schools.
844 Additionally, 22% of girls in intervention schools felt that MHM education enabled them
845 to discuss menstruation with their parents and others, whereas only 9% of girls in non-
846 intervention schools felt confident enough to engage in such discussions (Fig. 2).
847 The intervention enabled most schools to access water, which girl students used to enhance
848 safe hygiene practices. For instance, 41% of girls in intervention schools used water for
849 washing sanitary pads and 17% for genital cleaning, compared to 24% and 8% of girls in
850 non-intervention schools, respectively (Fig. 4).
851 The use of water for safe hygiene practices among girl students revealed a significant
852 difference between intervention and non-intervention school with P values less than 0.05
853 (P < α; 0.001 < 0.05),(Table 3), indicating a statistically significant difference and
854 supporting the rejection of the null hypothesis in favour of the alternative one.

855

856 The findings align with the studies from Bangladesh and Nepal, which reported that access
857 to menstrual hygiene education and WASH services reduces stress and discomfort among
858 girls while improving hygiene practices [3]. Similarly, a research finding in India indicated
859 that access to WASH services and enhanced hygiene practices help mitigate negative health
860 impacts, such as infections associated with reusable pads [24].
861 However, the health centre report in the study district, identifying diarrhoea as WASH-
862 related challenge-affecting students, including girls, was not supported by both the
863 qualitative and quantitative findings of this study. Girls, in fact, ranked diarrhoea as the
864 least significant cause of their class absenteeism (Fig. 6).
865 Girls and mothers’ response, however, might have been influenced by cultural factors, as
866 "diarrhoea" carries a negative connotation in the community, where the local term for it is
867 often used as an insult. This cultural stigma may have led focus group participants to feel
868 hesitant to address this issue directly.
869 Another possible explanation is that mothers and girls may not have distinguished diarrhoea
870 as a specific health concern, rather using the more generic term "sickness" instead. This
871 may explain the rankings provided by girls regarding their absenteeism (Fig. 6). In both
872 intervention and non-intervention schools, girls ranked "other types of sickness" fourth,
873 following family responsibilities, lack of sanitary pads, and menstrual hygiene-related
874 pains among six potential absenteeism causes. This suggests that diarrhoea may have been
875 overlooked or subsumed under the broader label of "other sickness."
876 The findings regarding the impact of WASH intervention on girls' health underscore that
877 school initiatives have the potential to enhance the emotional, social and physical well-
878 being of girls. This highlights the importance of implementing school hygiene and WASH
879 interventions.
880 **Effects of School Interventions on Class Attendance:** The effects of menstruation on
881 girls’ class absenteeism have been a prominent topic of study since menstrual hygiene
882 management (MHM) emerged as a research focus. However, robust evidence on the extent
883 to which menstruation impacts girls' class attendance remains limited [28]. Thus, this study
884 also included a research hypothesis stating, "School MHM and WASH interventions have
885 an impact on or improve girls' class attendance."
886 Qualitative results indicated that menstruation was not a primary reason for girls’
887 absenteeism. Instead, absenteeism was more closely linked to family responsibilities, such
888 as caregiving when a family member is sick, as well as the girls' own health issues.
889 However, mothers from non-intervention schools noted that lack of hygiene management
890 services, such as access to water and sanitary pads, posed significant challenge for girls
891 during menstruation. Additionally, girls from non-intervention schools mentioned, “Most
892 girl students do not change sanitary pads at school due to a lack of access to water, sanitary
893 pads and girl-friendly toilets."
894 In contrast, girls from intervention schools stated, "Girls change sanitary pads in both the
895 sex-segregated toilets and girls’ club rooms. A teacher facilitating the MHM Club at the
896 intervention school also added, "During menstruation, girls prefer to come to school
897 because they receive better hygiene services here than they do at home."
898 Conversely, a principal and MHM club facilitator from a non-intervention school
899 acknowledged the absence of hygiene services to support girls' needs, saying, "Although
900 girls do not explicitly tell us they are absent due to menstruation, we believe that inadequate
901 school hygiene services contribute to their absenteeism."
902 The qualitative results indicate that while menstruation itself may not directly cause
903 absenteeism, the lack of adequate hygiene services in schools does contribute to girls’
904 absences during this time. Therefore, it is crucial to recognize that menstruation cannot be
905 viewed in isolation from the availability of school hygiene services.
906 Quantitative results further clarified qualitative findings. For instance, 68.3% of girls from
907 intervention schools reported attending school during menstruation, compared to only
908 29.8% from non-intervention schools. About 48% of girls from non-intervention schools
909 acknowledged that they did not attend school during menstruation due to a lack of sanitary
910 products and water, compared to only 15.6% of girls in intervention schools (Fig. 5).
911 Girls' absenteeism was also linked to social relationships, as teasing from boys during
912 menstruation could increase absences. For example, 21% of girls in intervention schools
913 reported feeling comfortable discussing menstruation and menstrual hygiene with boys,
914 compared to only 8.9% of girls in non-intervention schools. Additionally, 21.7% of girls in
915 intervention schools felt that MHM education helped them discuss menstruation with
916 parents and others, while only 9.2% of girls in non-intervention schools shared this
917 confidence (Fig. 2).
918 Thus, the qualitative findings underscore the importance of both the physical and social
919 environments in schools for supporting girls’ hygiene practices. Meanwhile, the
920 quantitative results provided measurable insights into how these factors influence girls’
921 class attendance.
922 The difference in attendance between girls from intervention and non-intervention schools
923 was statistically significant (Table 4), with a P-value of 0.001, which is less than 0.05.
924 Therefore, the null hypothesis was rejected, and the alternative hypothesis was accepted.
925 This finding aligns with the North Ethiopia Tigray Region, study finding which
926 demonstrated that providing sanitary pads and MHM education improved girls' class
927 attendance [8]. Similarly, a study in Ghana found a significant increase in attendance after
928 providing sanitary products and adolescent education [14]. A systematic review on the
929 impacts of school water and sanitation also concluded that the absence of water and
930 sanitation services contributes to absenteeism during menstruation [16]. Furthermore, a
931 study in Kenya found that access to school WASH services significantly reduced
932 absenteeism among primary school students [20].
933 Conversely, the finding of this study contrasts with two other studies. For instance, a
934 research finding in Nepal found that girls’ absenteeism did not decrease with the provision
935 of menstrual cups [9]. Similarly, a study in Malawi reported that varying levels of WASH
936 service access did not significantly affect absenteeism among girls in different schools [19].
937 However, the contexts of Nepal and Malawi studies differ significantly from this study.
938 The Nepal study focused solely on menstrual cup provision without addressing other
939 hygiene services or social factors within and outside the school environment that can
940 significantly influence girls’ hygiene behaviours. Similarly, the Malawi study did not
941 consider behaviour change promotion related to the use of school WASH services, access
942 to and use of sanitary products and outside school environments.
943 This study demonstrates the need for adopting a comprehensive and targeted hygiene and
944 WASH intervention to effectively promote behaviour change among female students. In
945 schools where adequate hygiene services are lacking, menstruation can contribute to
946 increased absenteeism. Thus, it is crucial to evaluate menstruation's impact on absenteeism
947 in relation to the availability of hygiene services within the school. Furthermore, as female
948 students are affected by social dynamics both within and outside the school environment,
949 it is important to consider their external contexts when examining hygiene practices and
950 attendance.
951 School interventions not only influenced girls’ class attendance but also positively affected
952 their study time at home. Although intervention and non-intervention schools are situated
953 in the same rural communities, girls in intervention schools felt confident discussing and
954 persuading their parents to reduce household tasks and allow more study time, unlike their
955 counterparts who were less confident. Ultimately, while it may not be possible to eliminate
956 all factors contributing to girls' absenteeism, effective school interventions can significantly
957 reduce menstruation-related absences.
958 **Impacts of Class Attendance on Academic Performance:-**Based on the preceding
959 discussions, the findings indicate that school interventions significantly improved girls'
960 class attendance/reduced menstruation related absenteeism significantly. Therefore, the
961 research hypothesis for this subtopic was, "School MHM and WASH interventions,
962 through improved class attendance, positively influence girls' academic performance**.**"

| 963 | The study revealed that, in addition to increased class attendance, the school intervention |
| --- | --- |
| 964 | significantly enhanced girls' study time at home. For instance, 77.6% of girls from |
| 965 | intervention schools reported having sufficient time to study at home, compared to only |
| 966 | 41.1% of girls from non-intervention schools. Additionally, 53% of girls in non- |
| 967 | intervention schools reported a lack of study time due to family responsibilities, whereas |
| 968 | only 10.9% of girls in intervention schools faced the same issue (Fig. 7). |
| 969 | The t-test results indicated a statistically significant difference in study time between girls |
| 970 | from intervention and non-intervention schools, with a p-value of 0.001, which is |
| 971 | significant at the 0.05 level (Table 5). The next question, therefore, is whether these |
| 972 | improvements in class attendance and study time for girls in intervention schools translated |
| 973 | into improved academic performance. |
| 974 | To evaluate and compare girls’ academic performance between the two categories of |
| 975 | schools, the researcher analysed students' average scores from the first semester and used |
| 976 | rating scales such as "excellent," "very good," "good," and "fair." In intervention schools, |
| 977 | the percentages of girls receiving these ratings in order were 2%, 4%, 14%, and 29%, |
| 978 | respectively, out of 333 students. In comparison, in the non-intervention schools girls had |
| 979 | the ratings of 3%, 9%, 16%, and 25% with similar order from the 293 students across the |
| 980 | three non-intervention schools (Fig. 7). |
| 981 | In summary, 12% of girls from non-intervention schools received "very good" and |
| 982 | "excellent" ratings, while only 6% of girls from intervention schools achieved these ratings, |
| 983 | less than half the percentage observed in non-intervention schools. However, for the ratings |
| 984 | of "good" and "fair," girls from intervention schools outperformed their peers, with 43% |
| 985 | achieving these ratings compared to 41% in non-intervention schools. |
| 986 | These findings contrast with prior research that established a positive correlation between |
| 987 | class attendance and academic performance [21, 22]. For instance, a study titled "Class |
| 988 | Attendance and Learning Outcomes" found that attendance was positively associated with |
| 989 | improved performance among low-achieving students [23]. This observation partially |
| 990 | aligns with the results for low-performing girls in intervention schools, where 43% received |
| 991 | "good" and "fair" ratings compared to 41% in control schools; however, this difference is |
| 992 | not statistically significant. |
| 993 | The study results reports that high-performing students were largely unaffected by class |
| 994 | attendance, while the impact of attendance on low-performing students was minimal. This |
| 995 | could be due to the fact that the comparison relied solely on first-semester average scores. |
| 996 | Differences may have been more pronounced if performance had been analysed over an |
| 997 | entire academic year. |
| 998 | In addition, the number of high-performing students differed between the two categories |
| 999 | of schools, with more students achieving "excellent" and "very good" ratings in non- |
| 1000 | intervention schools compared to intervention schools. This discrepancy suggests that |
| 1001 | factors beyond class attendance may also contribute to academic performance. |
| 1002 |  |

1003

1004

1005

1006

1007

1008

1009

1010

1011

1012

1013

1014

1015

1016

1017

1018

1019

1020

1021

1022

1023

1024

1025

1026

1027

1028

1029

1030

1031

1032

1033

1034

1035

1036

1037

1038

1039

1040

1041

1042

#### 5. Conclusion

School-based MHM and WASH interventions provide multiple benefits for menstruating girls. They help build confidence in communicating with peers, teachers, and parents, enhance MHM skills, and improve girls' ability to negotiate for study time and reduced domestic workload.

Additionally, these interventions support physical, emotional, and social well-being by promoting safe MHM practices, emotional regulation, and positive social interactions. They also reduce class absenteeism by providing sanitation and hygiene facilities and fostering supportive school environments, though domestic responsibilities and menstrual pain remain significant barriers.

Despite improvements in attendance, study time, and social engagement, the impact on academic performance is minimal, suggesting that other factors influence learning outcomes.

The findings emphasize the need for comprehensive, sustained interventions tailored to girls’ diverse needs to improve MHM practices, empowerment, health, and school attendance. Continued support from school programs is essential for long-term adoption of MHM practices and resilience to related challenges.

This study contributes to closing knowledge gaps in MHM and absenteeism research, particularly regarding girls' empowerment, health, and academic performance, and serves as a foundation for future studies.

Finally, the findings have implications for policy makers and organizations involved in girls' education. Ethiopia has a supportive policy framework for school MHM and WASH interventions, but inadequate budget allocation limits implementation. Regional and district WASH sector bureaus should allocate sufficient funds to improve these services.

Non-governmental organizations, as key drivers of school MHM and WASH initiatives, should strengthen collaboration with government agencies, actively engage girls, and design comprehensive school WASH programs.

~~The school intervention study demonstrated that school hygiene, water and sanitation (WASH)~~ ~~interventions have numerous positive effects on female students. The interventions significantly~~ ~~boosted their confidence in discussing menstruation and lowered anxieties. Beyond fostering a~~ ~~supportive school environment, the interventions positively influenced girls’ emotional, social,~~ ~~and physical well-being. Additionally, the study found that school interventions could reduce~~ ~~menstruation-related absenteeism, although less effective in directly improving academic~~ ~~performance.~~

1043

**Formatted:** Indent: Left: 0.1", Space Before: 0 pt, After: 0 pt

1044

~~Therefore, this research provides valuable insights into the challenges faced by female~~ ~~students and stands out from previous studies due to its use of long-term school~~

1045

1046

1047

1048

1049

1050

1051

1052

1053

~~interventions and exploration of new dimensions such as empowerment, health, and~~ ~~educational performance. By addressing key knowledge gaps, the findings hold promise to~~ ~~encourage local government decision-makers and non-governmental organizations to~~ ~~further advance school hygiene and WASH initiatives. In addition, in sights on girls’~~ ~~empowerment, physical, emotional and social aspects of health may trigger researchers to~~ ~~expand the area of research, besides the most commonly researched theme, ‘’effects of~~ ~~menstruation on girls’ class attendance’’~~.

1054

1055

1056

1057

1058

1059

1060

1061

1062

1063

1064

1065

1066

1067

1068

1069

1070

1071

1072

1073

1074

1075

1076

1077

1078

**Acknowledgements:** The authors recognize the contribution made by Ato Yalew Tizazu and Wondemu Sisay who supported the research during data collection.

#### 6. References

1. Ethiopian Ministry of Health. National Adolescent and Youth Health Strategy.2018; Available from:[https://www.prb.org/wp-content/uploads/2018/05/National-Adolescent-and-Youth-](https://www.prb.org/wp-content/uploads/2018/05/National-Adolescent-and-Youth-Health-%20Strategy) [Health- Strategy](https://www.prb.org/wp-content/uploads/2018/05/National-Adolescent-and-Youth-Health-%20Strategy)
2. Crofts T, Coates S, FisherJ. Menstruation hygiene management for school girls. 2014; Available from: <http://wedc.lboro.ac.uk/wedc.lboro.ac.uk/knowledge>
3. Bhusal CK. Practice of menstrual hygiene and associated factors among adolescent school girls in Dang District, Nepal. *Advances in Preventive Medicine*.2020; http://doi:10.1155/2020/1292070.
4. Tamiru S, Mamo K, Acidria P, Mushi R, Ali CS, Ndebele L. Towards a sustainable solution for school menstrual hygiene management: Cases of Ethiopia, Uganda, South-Sudan, Tanzania,

| 1079 | and Zimbabwe. Sahin M, editor. Waterlines.2015;34:[http://*doi.org/10.3362/1756-*](http://doi.org/10.3362/1756-) |
| --- | --- |
| 1080 | *3488.2015.009* |
| 1081 | 5. Seydou F, Ama-dou B, Abdoulaye S, Alassane T, Oumar TS, Ibrahima K, et al. Menstrual |
| 1082 | hhygiene mmanagement in school ssetting in two ssecondary sschools in the Bamako |
| 1083 | District, Case of the School “La Chaine Grise” and the sschool cheick Modibo Diarra. |
| 1084 | 2020; http:// doi.org/10.4236/ojog.2020.1020025 |
| 1085 | 6. Sommer M, Hirsch JS, Nathanson C, Parker RG. Comfortably, Safely, and Without Shame: |
| 1086 | Defining Menstrual Hygiene Management as a Public Health Issue. American Journal of Public |
| 1087 | Health. 2015; <https://ajph.aphapublications.org/doi/full/10.2105/AJPH.2014.302525> |
| 1088 | 7. Tegegne TK, Sisay MM. Menstrual hygiene management and school absenteeism among female |
| 1089 | adolescent students in Northeast Ethiopia. BMC Public Health. 2014;14:http// |
| 1090 | [doi/10.1186/1471-2458-14-1118](https://doi/10.1186/1471-2458-14-1118) |
| 1091 | 8. Belay S, Kuhlmann AKS, Wall LL. Girls’ attendance at school after a menstrual hygiene |
| 1092 | intervention in northern Ethiopia. International Journal of Gynecology & Obstetrics. 2020; |
| 1093 | 149:287–91. http:// doi: 10.1002/ijgo.13127 |
| 1094 | 9. Oster E, Thornton R. Menstruation, Sanitary Products, and School Attendance: Evidence from |
| 1095 | a Randomized Evaluation. American Economic Journal: Applied Economics. 2011; 3: 91–100. |
| 1096 | [http://doi:](http://doi/) 10.1257/app.3.1.91 |
| 1097 | 10.Haver,J,Caruso, BA, Ellis, A, Sahin, M, Villasenor, JM, Andes, KL, Freeman MC. WASH in |
| 1098 | Schools Empowers Girls’ Education in Masbate Province and Metro Manila, Philippines: An |
| 1099 | assessment of menstrual hygiene management in schools.2013; avaialbel from |
| 1100 | [https://www.unicef.org/wash/schools.](https://www.unicef.org/wash/schools) |
| 1101 | 11. United Nations Children’s Fund and Columbia University. WASH in schools empowers girls’ |
| 1102 | education: Proceedings of the annual virtual conference on menstrual hygiene management in |
| 1103 | schools.2017; available from https://[www.washinschoolsindex.com](http://www.washinschoolsindex.com/) |
| 1104 | [/storage/articles/ck1he0Zuxts5R7dNgMiPtrRF2SuCKjP54b3VbjnA.](https://www.washinschoolsindex.com/storage/articles/ck1he0Zuxts5R7dNgMiPtrRF2SuCKjP54b3VbjnA) |
| 1105 | 12. Woodall J, Raine G, South J, Warwick-Both L. Empowerment , health and well-being. 2010; |
| 1106 | available from <https://core.ac.uk/download/pdf/42412714.pdf> |
| 1107 | 13. Prüss-Üstün A, World Health Organization. Safer water, better health: Costs, benefits and |
| 1108 | sustainability of interventions to protect and promote health. 2008; available from |
| 1109 | <https://apps.who.int/iris/handle/10665/43840> |
| 1110 | 14. Samuel KA, Ebenezer EYA, Nang BD, George O, Charles CA, et al. WASH in selected basic |
| 1111 | schools and possible implications on health and academics: An example of the Wa |
| 1112 | Municipality of Ghana, West Africa. American Journal of Environmental Science and |
| 1113 | Engineering. 2021; 5:15-20. http:// [doi.org/10.11648/j.ajese.20210501.13](https://doi.org/10.11648/j.ajese.20210501.13) |
| 1114 | 15.Birdthistle I, Dickson K, Freeman M, Javidi L. What impact does the provision of separate toilets |
| 1115 | for girls at schools have on their primary and secondary school enrolment, attendance and |
| 1116 | completion? A systematic review of the evidence. EPPI-Centre, Social Science Research Unit, |
| 1117 | Institute of Education, University of London. 2011; ISBN: 978-907345: available from |
| 1118 | https;//[WWW.educ-links.org](http://WWW.educ-links.org/) |
| 1119 | 16. Jasper C, Le T-T, Bartram J. Water and sanitation in schools: A systematic review of the health |
| 1120 | and educational outcomes. Research Gate. 2012; http:// doi 10.3390/ijerph9082772. |

1121

1122

1123

1124

1125

17. *World Health Organization. Basic Documents,49ed. 2020; available from* [*https://apps.who.int/gb/bd/pdf_files/B*](https://apps.who.int/gb/bd/pdf_files/BD_49th-en.pdf%20.V12) *D_49th-en.pdf .V12*
18. Oster E, Thornton R. Menstruation, sanitary products, and school attendance: Evidence from a randomized evaluation. American Economic Journal.2011;3:91–100.[http://doi:](http://doi/) 10.1257/app.3.1.91

1126

1127

1128

1129

1130

1131

1132

1133

1134

1135

1136

1137

1138

1139

1140

1141

1142

1143

1144

1145

1146

1147

1148

1149

1150

1151

1152

1153

1154

1155

1156

1157

1158

1159

1160

1161

1162

1163

1164

1165

1166

1167

1168

1169

19. Grant MJ, Lloyd CB, Mensch BS. Menstruation and school absenteeism: Evidence from rural Malawi. Comparative Education Review. 2013; http:// doi: 10.1086/669121
20. Freeman MC, Greene LE, Dreibelbis R, Saboori S, Muga R, Brumback B, et al. Assessing the impact of a school-based water treatment, hygiene and sanitation programme on pupil absence in Nyanza Province, Kenya: A cluster-randomized trial. Tropical Medicine & International Health. 2011; http:// doi:10.1111/j.1365-3156.2011.02927.x
21. [Tetteh GA.](https://www.emerald.com/insight/search?q=Godson%20Ayertei%20Tetteh) Effects of classroom attendance and learning strategies on the learning Outcome. *[Journal of International Education in Business](c:\\pdfbuilder\\temp\\Journal of International Education  in  Business)*[.](c:\\pdfbuilder\\temp\\Journal of International Education  in  Business) 2018; 11: http:// [doi.org/10.1108/JIEB-01-2017-](https://doi.org/10.1108/JIEB-01-2017-0004) [0004](https://doi.org/10.1108/JIEB-01-2017-0004)
22. Al Hazaa K , Abdel-Salam,ASG, Ismail R, Johnson C, Al-Tameemi R A N, Romanowski MH, etal. The effects of attendance and high school GPA on student performance in first-year undergraduate	courses. *Cogent	Education.	2021;8*: <https://doi.org/10.1080/2331186X.2021.1956857>
23. *Kwak DW, Sherwood C, Tang KK*. [**C**lass attendance and learning outcom**e**:](https://ideas.repec.org/a/spr/empeco/v57y2019i1d10.1007_s00181-018-1434-7.html) [Empirical](https://ideas.repec.org/s/spr/empeco.html) [economics](https://ideas.repec.org/s/spr/empeco.html) 2019; 57: 177-203. [http://doi:](http://doi/) 10.1007/s00181-018-1434-7
24. Das P, Baker KK, Dutta A, Swain T, Sahoo S, Das BS, et al. Menstrual hygiene practices, WASH access and the risk of urogenital infection in women from Odisha, India. Journal.pone.2015; http:// doi: 10.1371/journal.pone.0130777: PMC4488331.
25. Glanz K, Rimer BK, Viswanath K. Health behaviour and health education: Theory, research and practice. **Jo**ssey-Bass.2008; <https://psycnet.apa.org/fulltext/2008-17146-000.pdf>
26. Krejcie RV, Morgan DW. Determining sample size for research activities: Educational and psychological	measurement.	Sage	publications.1970;30:607–10.http:// doi/10.1177/001316447003000308
27. Miiro G, Rutakumwa R, Nakiyingi-Miiro J, Nakuya K, Musoke S, Namakula J, et al. Menstrual health and school absenteeism among adolescent girls in Uganda: A feasibility study. BMC Women’s Health. 2018; 18: http:// doi 10.1186/s12905-017-0502-z
28. Sumpter C, Torondel B. A systematic review of the health and social effects of menstrual hygiene management. PLoS ONE. 2013;8: http:// doi:10.1371/journal.pone.0062004

##### 7. Supporting documents

**S1 Figure. *Girls’ hygiene knowledge comparison between two groups of schools***

Girls from intervention schools benefited from school peer-to-peer education and teachers who took MHM trainings. This helped girls from intervention school to demonstrate improved hygiene knowledge and practice compared to non-intervention schools who lucked these opportunities.

###### S2 Figure: Genital hygiene practice comparison between intervention and non-intervention girls during menstruation

| 1170 | **S3 Figure: Menstruating girls’ confidence to request menstrual break** |
| --- | --- |
| 1171 | **during classes** |
| 1172 | Menstruating girls from intervention school improved their social relations with boys and |
| 1173 | teachers due to the school intervention and this was reflected in their confidence to request |
| 1174 | their teachers, male and female, for menstrual break during classes, unlike the non- |
| 1175 | intervention school girls who were limited to female teachers***.*** |
| 1176 | ***S1Table: Comparing* girls’ experience to change sanitary pads at** |
| 1177 | **schools** |
| 1178 | In non-intervention schools, girls lacked access to dedicated clubrooms where they could change |
| 1179 | sanitary pads, rest when feeling unwell and exchange hygiene education. This absence significantly |
| 1180 | impacted their comfort and support for managing menstrual health at school, leading to a much higher |
| 1181 | proportion of girls refraining from changing sanitary pads during school hours—44% compared to just |
| 1182 | 4.2% of the girls from intervention schools. |

Response to Reviewers

PONE-D-24-55215

Effects of school menstrual hygiene, water and sanitation interventions on girls’ empowerment, health and educational outcomes: Lasta District, Amhara Regional State, Ethiopia

PLOS ONE

Dear Dr. Andargie,

Thank you for submitting your manuscript to PLOS ONE. After careful consideration, we feel that it has merit but does not fully meet PLOS ONE’s publication criteria as it currently stands. Therefore, we invite you to submit a revised version of the manuscript that addresses the points raised during the review process.

All three reviewers have provided extensive comments to the writing of the manuscript which need to be addressed.

Please submit your revised manuscript by Feb 21 2025 11:59PM. If you will need more time than this to complete your revisions, please reply to this message or contact the journal office

at [plosone@plos.org.](mailto:plosone@plos.org) When you're ready to submit your revision, log on to https://[www.editorialmanager.com/pone/](http://www.editorialmanager.com/pone/) and select the 'Submissions Needing Revision' folder to locate your manuscript file.

Please include the following items when submitting your revised manuscript:

- A rebuttal letter that responds to each point raised by the academic editor and reviewer(s). You should upload this letter as a separate file labeled 'Response to Reviewers'.
- A marked-up copy of your manuscript that highlights changes made to the original version. You should upload this as a separate file labeled 'Revised Manuscript with Track Changes'.
- An unmarked version of your revised paper without tracked changes. You should upload this as a separate file labeled 'Manuscript'.

If you would like to make changes to your financial disclosure, please include your updated statement in your cover letter. Guidelines for resubmitting your figure files are available below the reviewer comments at the end of this letter.

If applicable, we recommend that you deposit your laboratory protocols in protocols.io to enhance the reproducibility of your results. Protocols.io assigns your protocol its own identifier (DOI) so that it can be cited independently in the future. For instructions

see: https://journals.plos.org/plosone/s/submission-guidelines#loc-laboratory-protocols. Additionally, PLOS ONE offers an option for publishing peer-reviewed Lab Protocol articles, which describe protocols hosted on protocols.io. Read more information on sharing protocols

at https://plos.org/protocols?utm_medium=editorial- email&utm_source=authorletters&utm_campaign=protocols.

We look forward to receiving your revised manuscript. Kind regards,

Alison Parker Academic Editor PLOS ONE

Journal Requirements:

When submitting your revision, we need you to address these additional requirements.

1. Please ensure that your manuscript meets PLOS ONE's style requirements, including those for file naming. The PLOS ONE style templates can be found at

https://journals.plos.org/plosone/s/file?id=wjVg/PLOSOne_formatting_sample_main_body.pdf and

https://journals.plos.org/plosone/s/file?id=ba62/PLOSOne_formatting_sample_title_authors_affiliation s.pdf

2. Please include your full ethics statement in the ‘Methods’ section of your manuscript file. In your statement, please include the full name of the IRB or ethics committee who approved or waived your study, as well as whether or not you obtained informed written or verbal consent. If consent was waived for your study, please include this information in your statement as well.
3. Please ensure that you refer to Figure 8 in your text as, if accepted, production will need this reference to link the reader to the figure.
4. Please review your reference list to ensure that it is complete and correct. If you have cited papers that have been retracted, please include the rationale for doing so in the manuscript text, or remove these references and replace them with relevant current references. Any changes to the reference list should be mentioned in the rebuttal letter that accompanies your revised manuscript. If you need to cite a retracted article, indicate the article’s retracted status in the References list and also include a citation and full reference for the retraction notice.

Response: Full ethics statement is presented under methods, sub-section 2.4 ethical issues. The ethical

letter was shared, together with the manuscript and it can be shared again if necessary. In addition, guiding templates for the title and the body have been used to design the manuscript; references reviewed for errors. This research was non-clinical and therefore, laboratory protocol is not applicable for this study. Fig 8 reviewed. Figures used in the manuscript are changed to tif file using PACE and uploaded separately from the manuscript. However, the manuscript maintains figure numbers and captions	on	separate	pages	where	figures	should	be	placed.

[Note:	HTML	markup	is	below.	Please	do	not	edit.] Reviewers'	comments:

Reviewer's Responses to Questions

Comments to the Author

1. Is the manuscript technically sound, and do the data support the conclusions?

The manuscript must describe a technically sound piece of scientific research with data that supports the conclusions. Experiments must have been conducted rigorously, with appropriate controls, replication, and sample sizes. The conclusions must be drawn appropriately based on the data presented.

Reviewer #1: Partly

Reviewer #2: Yes

Reviewer #3: Yes

2. Has the statistical analysis been performed appropriately and rigorously?

Reviewer #1: Yes

Reviewer #2: Yes

Reviewer #3: No

3. Have the authors made all data underlying the findings in their manuscript fully available?

The PLOS Data policy requires authors to make all data underlying the findings described in their manuscript fully available without restriction, with rare exception (please refer to the Data Availability Statement in the manuscript PDF file). The data should be provided as part of the manuscript or its supporting information, or deposited to a public repository. For example, in addition to summary statistics, the data points behind means, medians and variance measures should be available. If there are restrictions on publicly sharing data—e.g. participant privacy or use of data from a third party— those must be specified.

Reviewer #1: Yes

Reviewer #2: Yes

Reviewer #3: Yes

4. Is the manuscript presented in an intelligible fashion and written in standard English?

PLOS ONE does not copyedit accepted manuscripts, so the language in submitted articles must be clear, correct, and unambiguous. Any typographical or grammatical errors should be corrected at revision, so please note any specific errors here.

Reviewer #1: Yes

Reviewer #2: Yes

Reviewer #3: Yes

5. Review Comments to the Author

Please use the space provided to explain your answers to the questions above. You may also include additional comments for the author, including concerns about dual publication, research ethics, or publication ethics. (Please upload your review as an attachment if it exceeds 20,000 characters)

Reviewer #1: The author needs to have all the grammatical errors corrected before submitting the paper for review. Some paragraphs are too wordy, this needs to be addressed without interfering with the meaning. There is poor flow of sentences as well, Some parafraphs do not trelate to the previous ones

Response: The authors made extensive editing to improve word economy, clarity ,grammar error and coherence for the full manuscript.

Reviewer #2: I appreciate the work addressing this timely public health issue. I would like to raise few concerns regarding the study:

1. Method section

You mentioned that you utilized both methods. Could you clarify the purpose of the cross-sectional study? Additionally, what type of experimental design did you implement (e.g., true randomization, quasi-experiment, longitudinal, etc.)?

Response: The school MHM and WASH intervention began in July 2018; however, the study on the school intervention started in April 2023. Therefore, it was not possible to conduct a baseline survey, as time had already passed by the time the study began. Therefore, it was not possible to contrast baseline data with school interventions’ outcomes. Instead, the study used control schools that did not have school interventions to compare against the schools that received the WASH interventions. Consequently, longitudinal study design was not feasible due to the absence of baseline data and therefore, the study used cross-sectional design to measure the commutative effect of the school intervention by comparing with control schools. The study used quasi experimental design, details indicated under the following section.

For the detail see under methods, section, 2.2 Study design

Sampling method: The study district, Last District Education Office, identified 35 schools that had severe WASH service shortages before the beginning of the school intervention. Therefore, any non-governmental organization that intended to support schools could select any one of these schools, depending on its budget and work force capacity. Plan International Ethiopia, an international non-governmental organization, randomly selected 10 schools in 2018 for school MHM and WASH interventions. Therefore, the study randomly selected three sample schools among 10 WASH intervention schools to evaluate changes caused by the school intervention.

Similarly, for the control schools, from 25 schools that did not have school WASH interventions, the study selected randomly three sample schools.

Details indicated under Sampling method; section 2.5

2. Details of the Intervention

The description of the intervention lacks specificity in terms of quantity, frequency, and duration. Additionally, the content of the intervention is described too superficially, making it difficult to derive meaningful insights or formulate policy recommendations based on the findings.

Response: The school intervention incorporated a comprehensive approach to menstrual hygiene management (MHM), including MHM education, access to sanitary products (with training on how to prepare these products), provision of clean water, sex-segregated latrines, and a dedicated MHM room. The MHM room served as a space where girls could change sanitary pads and engage in peer-to-peer education. Soap was also provided to promote proper hygiene practices. The MHM education included both girls and boy students to get the support of boys.

Quantity: For each WASH intervention school, the program provided integrated MHM and WASH services. These services included access to sex-segregated latrines, tailored to the existing facilities at each school. For example, if a school already had one improved latrine, the program built one additional latrine exclusively for girls. However, if a school had no latrines, the intervention constructed two—one for girls and one for boys.

The program also provided MHM education, one MHM room per school, and sanitary products sufficient to last six months. These products included clean fabric cloths for sanitary product

preparation, with the quantity determined by the number of students in each school. Additionally, access to water was ensured through piped systems, from either nearby community water sources or directly within the schools.

MHM education continued in each school until all students had participated, primarily through peer-to-peer education and training sessions.

Frequency: School interventions are limited by budget and implementation timelines. Once schools receive the necessary support and training, they are expected to sustain these services in collaboration with the district education office. However, to ensure a smooth transition and adequate experience, the intervention continued its support until January 2024.

The frequency of support varied based on the nature of the services. Some services, such as latrines, are designed for long-term use, while others, like sanitary pads, have shorter lifespans. For instance, the intervention provided sanitary pad support twice. However, to address shortages, girls were trained to prepare sanitary pads themselves using fabric cloths supplied by the program and other accessible local materials.

Regarding MHM education, once all students were reached through peer-to-peer training, MHM club members took responsibility for advising pubertal girls and providing education to newcomers, especially at the start of the academic year. Similarly, soap was provided twice by the intervention, but menstruating girls contributed a small monthly amount to purchase soap during shortages.

For water access, maintenance was often required. Schools managed these maintenance activities in collaboration with the district education office. However, as the schools and district education office had budget shortage, the school intervention/program was managing all maintenance works until the end of the intervention.

Moreover, to ensure the proper functioning of the school intervention and equip intervention schools with the necessary skills, school program facilitators conducted consistent follow-ups from July 2018 to January 2024, providing both technical and financial assistance.

Duration: The school intervention spanned over six years, from July 2018 to January 2024. However, its activities were primarily conducted during school terms and were interrupted at times due to the COVID-19 pandemic and months lost to conflict in the district.

Content of intervention: There were distinct differences between intervention and control schools. While intervention schools had access to the services outlined under "quantity" above, control schools lacked all of these services due to the absence of similar interventions. The only support available to control schools was general awareness on MHM, provided by the district's "Women and Children Affairs Office" in line with the national education policy, along with non-sex-segregated latrines. Control schools did not have access to water, soap, sanitary products, peer-to-peer education and dedicated MHM rooms.

Included under methods, section 2.1 Description of school interventions

3. Contamination

The manuscript does not clearly outline the strategies implemented to prevent contamination or to buffer against potential biases.

Response: The intervention and control schools are located in rural areas where communities are sparsely populated, and schools are situated far apart. Additionally, due to gender-based violence and security concerns, girls in the rural areas do not travel long distances to attend school. As a result, only girls living near schools have the opportunity to pursue their education.

This geographical isolation meant there was no opportunity for MHM knowledge to be transferred from one school to another, as the distance between schools was significant. Furthermore, there was no deliberate knowledge transfer from intervention schools to control schools. To ensure the validity of the study, the authors verified the absence of other WASH interventions in the control schools or nearby areas before including them in the study.

Therefore, to address the issue of contamination, the study-employed strategies including conducting the research among rural schools, verifying the absence of WASH interventions in the control schools, and ensuring that no deliberate attempts were made to transfer knowledge from intervention to control schools.

Included under methods, section, 2.1 Description of school intervention-last part

4. Ethical Considerations

It is important to understand how the researchers addressed ethical dilemmas faced by non- intervention schools. Clarification on this matter is necessary.

Students under the age of 18 are included in your study. In this case, you must obtain both assent from the students and consent from their parents. Why was not assent considered?

Response: **Ethical Dilemma:** The control schools were not provided with MHM education or WASH services—not because of the research or for the purposes of the study, but due to budget constraints faced by the district education office in providing hygiene facilities and WASH services to all schools. In addition, the school intervention targeted only 10 schools in the district, based on its financial and work force capacity, while there were more schools needing support. The study found that 70% of schools in the district lacked access to water, and 67% did not have improved latrines. The control schools were among those still in need of WASH services, highlighting the broader challenge of resource limitations in the district.

**Students’ assent/consent**: As described in the methods section, students provided both verbal and written consent to participate in the study. Initially, the girls were contacted prior to the day of completing the questionnaire, and their verbal consent was obtained. The following day, after having sufficient time to discuss the matter with their parents and among themselves, those who volunteered to participate provided written assent. Each student signed the designated space on the questionnaire guide before beginning the response process. This

ensured that participation was voluntary and informed, with both verbal and written consent documented

**Guardian consent:** Before data collection, the study schools, in collaboration with the Parents and Teachers’ Association (PTA)—a body that fosters communication between schools and communities on educational matters—invited the parents of menstruating girls to attend a brief morning meeting. During the meeting, the school principal and PTA representatives provided an overview of the existing school hygiene facilities and services.

Parents were then asked for their permission to allow their daughters to share their views on the status of the school hygiene services. Therefore, verbal consent was obtained from parents, while those who participated in focus group discussions (FGDs), they also provided written consent by signing a consent form.

Included under methods, section: 2.4 ethical issues

5. Quality Control Mechanisms

What quality control mechanisms were in place to ensure the fidelity of the intervention, particularly regarding educational components? Blinding, randomization, and other quality control mechanisms need to be clearly articulated.

Response: Quality control: Randomization and blinding: Stratified sampling was used to group 10 intervention and 25 non-control schools based on their similarity in WASH experience. This was followed by selecting three schools randomly from each group. For the intervention schools, the 10 schools were given codes and the codes were mixed. From the mixed school codes, a person who was not part of the process picked three school codes randomly. This procedure was repeated for 25 control schools.

Similarly, to select menstruating girl students, a lottery method was used but it followed multistage sampling: First, all boy students were excluded from the sample, as the focus of the study was on girl students. Second, all non-menstruating girl students were excluded from the sample because the study aimed at evaluating the experience of menstruating girl students.

After the exclusion of boys and non-menstruating girls, the study team identified 333 and 293 menstruating girl students from intervention and control schools respectively. This made 626-study population. Using, the sample size formula and after inclusion of the probably of non-attendant rate, the sample size was 360. Thus, for the sample size, a separate list of girls was prepared for each of the six schools. In the study population, the contribution of the intervention schools was 53% while the control schools was 46.6%. Based on this contribution, 192 and 168 menstruating girls were allocated to intervention and control schools orderly.

Each school received its sample size/share based on its contribution to the study population. Using the prepared list of menstruating girls, a lottery method was used to identify samples from each school. For a school that had 50 menstruating girl students in the list but its sample share/size 25, the study team prepared 50 draws and mixed them. Among 50 draws/pieces of paper, 25 of them were given codes/numbers from 1-25, while the remaining were zero. Then, based on the order of the list of students, each student was asked to withdraw a code. If the draw reads one of the numbers from 1-25, a girl is part of the final sample but if the draw reads zero, a girl is excluded from the sample. Therefore, in both the school and girl student selection, the study used simple random techniques.

Blinding one of the quality controlling techniques used to avoid biases during data collection, data analysis, reporting, etc. from those involved in the research process. Though double blinding was not possible, single blinding was used. Participating girl students were blinded due to long years of program implementation in the school where they had been actively engaged from the start of the school intervention and they had different progress evaluation meetings. Thus, they took their participation in the study process as part of the program implementation not as a research that was different from the program implementation. For data collectors, the researcher provided data collectors’’ training where data collectors bias ’was one of the major components of the training.

In addition, to minimize the researcher’s bias, the study employed different approaches. It employed mixed method of data collection to improve the accuracy of the research finding; used clinical data (recorded from MHM related treatment) to cross check against field data from girl students; used independent samples T-test to avoid considering minor differences between intervention and control school as significant. Furthermore, when the literature reviews are observed, both negative and positive findings were equally presented; it tried to avoid confirmation bias, by employing socio- ecological perspectives that helped the study to consider different levels of factor analysis (individual, group, community, organization, and policy) , which were not common in the previous study designs.

Furthermore, the study used random sampling to determine the sample size and the sample represented 57% of the study population in the six study schools.

Included under methods, section, 2.5, sampling methods

6. Mixed Methods Approach

The implementation of mixed methods is noted. Please clarify the sequence of the qualitative and quantitative studies: which method was conducted first, and what was the rationale behind this choice?

Response: Yes, the study used both qualitative and quantitative methods to improve the strength of the evidence. In terms of sequence, qualitative method was used first to understand the views of the target groups and make any necessary change on the quantitateive method. However, there were also groups and individuals targeted by the qualitative method alone, like, office heads and girls’ mothers. In the second case, only qualitative methods was used and therefore, no sequence. School facility observation was another example.

Clarified under methods, section 2.3

***Institutional Written assent*:-** Additionally, the study obtained written consent from Lasta District Education Office that was responsible for monitoring, coordinating, and managing all schools in the district. **Under methods**, sub section 2.4

7. Controlling for Confounding Factor

Given that school performance can be influenced by multiple factors, it is essential to explain how the study controlled for the effects of other confounding variables on both school attendance and educational performance.

Response:

**Confounding Variables and Mitigation Strategies used**

Confounding variables are factors, other than the school interventions, that can influence both independent and dependent variables. To minimize the effect of confounding factors on girls’ class attendance and performance, the study employed the following strategies:

##### 1. Restriction:

The study restricted the population to girls of similar age and educational level to reduce variability caused by potential confounding variables. The majority of the sampled students were between 13 and 15 years old, all female, and at comparable educational level— students in the Primary 2nd Cycle Schools (Grades 7 and 8). These restrictions ensured that participants shared similar values for key confounding factors.

##### 2. Matching:

To control for potential confounding variables, girls in the intervention schools were matched with girls in the control schools who had similar characteristics. These included physiological factors, level of education, family educational background and income, age, exposure to technology, and school settings (both schools were located in rural areas). By ensuring that confounding factors were consistent between the two groups, any differences identified in the study could more confidently be attributed to the school interventions.

##### 3. Transportation and Academic Performance:

Since both intervention and control schools were in rural settings, students shared similar experiences regarding school travel. Almost all girls walked to school, with no notable differences in access to transportation. Additionally, the study accounted for prior academic performance by using mid-year educational performance data from 2024 for comparison, thereby mitigating the influence of pre-existing academic disparities.

##### 4. Teaching Quality and Learning Environment:

Although individual teacher differences could exist, significant differences in teaching quality were unlikely, as neither intervention nor control schools had access to advanced educational technologies or resources. Both were located in similar rural environments, ensuring comparable learning conditions.

##### 5. Cultural and Religious Homogeneity:

The majority of the girls from both intervention and control schools shared the same ethnic background (98% Amhara) and religious faith (84% Orthodox), which reduced the potential influence of cultural or religious differences on the study outcomes.

**Presented under Methods section, 2.6**

I recommend that all these concerns be addressed in the body of the manuscript to enhance its clarity and robustness.

Reviewer #3: Thank you for asking me to review this paper on “Effects of school menstrual hygiene, water, and sanitation interventions on girls’ empowerment, health, and educational outcomes: Lasta District, Amhara Regional State, Ethiopia." It is exciting research. However, the following issues must be improved before it is accepted for publication.

The title of this study, which is stated as "Effects of School Menstrual Hygiene, Water, and Sanitation Interventions on Girls’ Empowerment, Health, and Educational Outcomes,” needs modifications. The intervention section, as indicated in lines 184–188, includes MHM education and MHM rooms, which

are not included in this title. Therefore the title of this study should be modified to “Effects of School Menstrual Hygiene Management and Water, Sanitation and Hygiene (WASH) Service Interventions on Girls’ Empowerment, health and Educational Outcomes.”

Response: Improvement has been made to the title and abstract and so does on the rest of the of manuscript. However, the word’ services’ may not be necessary as the reference in the title is the

school program/the intervention in order to realize the MHM services . We felt it is also better to use the abbreviation , WASH, in the body, rather than in the title. Therefore, the revised title reads as’’

Effects of School Menstrual Hygiene Management, Water,Sanitation and Hygiene Interventions on

Girls’ Empowerment, Health and Educational Outcomes.’’

Abstract sections: lines 31–33 should be consistent with the modified title. Please modify it as “This study investigated the effects of school menstrual hygiene management and water, sanitation, and hygiene (WASH) service interventions on girls’ empowerment, health, and educational outcomes. And also use effect or impacts consistently throughout the documents.

Response: Similar change has been made to the abstract

The introduction sections: The authors well explained the context of the study. However, lines 157- 160 need rearrangements. Line 157, “…..The significance of this study...” should come after line 160. Design and setting: in line 173, the authors stated, “Six primary second-cycle schools were selected within the district” without describing first how many primary second-cycle schools are there in the district. The authors should also describe how six primary second-cycle schools were selected. Is randomization conducted? Or is there a baseline difference between exp’tal and controlled schools? Otherwise, it is difficult to conclude whether the effects after six years were due to interventions or results of their baseline difference.

| Response: Rearrangement on the indicated lines have been made; clarifications included on how the 6 study schools were selected and the methods used to select schools:- indicated under methods | |
| --- | --- |
| sections. 2.5. Sampling methods and sample size. | However, due to changes made following |
| reviewers’ comments, line numbers referred above many have changed. So, it may require going through many paragraphs to find changes. | |

Line 180: “10 primary second cycle schools or 6? This contradicts what was stated in line 173.

The informed consent section from line 234-262 needs modification. The informed voluntary consent for a minor (age < 18 years)/vulnerable individual should be signed by his/her legal competent representative (e.g., a parent/guardian). But in this study, even though around 85% of study participants were less than 16 years old (as indicated in table 1), informed voluntary consent taken from parent/guardian was not explained.

Response: The 10 primary 2nd Cycle schools were the intervention schools only; however, the six

schools are the study schools, three from the 10 intervention schools and another three from control

schools. To avoid confusion, additional clarifications are given under method, sub-section 2.1-

are added under methods, sub-section.2.4. Ethical issues.

description of school interventions and 2.5-Sampling method.

Regarding consent, detail explanations

Results section: Please rewrite from lines 284 to 288. For example, “Parental education levels were generally low...” Do not interpret the findings in the result section; keep it for the discussion section. Please remove the gender variable from table 1.

The conclusion section is not well written. The authors should stick to the findings of the study during the conclusion. For instance, line 764-765, ”The interventions significantly boosted their confidence in discussing menstruation and lowered anxieties,” should be removed as not a tool that measures anxiety was indicated in the result.

Please rewrite the structured recommendation for this study and remove lines 770–778.

Finally, there are numerous editorial and grammatical errors that should be corrected before this manuscript is accepted. For instance, the author should use MHM consistently throughout the documents once he/she defined menstrual hygiene management as (MHM), unless it comes after full stop or at the beginning of the paragraph.

Response: Manuscript conclusion and recommendations revised and improved, Section: 6

6. PLOS authors have the option to publish the peer review history of their article (what does this mean?). If published, this will include your full peer review and any attached files.

If you choose “no”, your identity will remain anonymous but your review may still be made public.

**Do you want your identity to be public for this peer review?** For information about this choice, including consent withdrawal, please see our Privacy Policy.

Reviewer #1: No

Reviewer #2: **Yes:** Balem Demtsu Betsu Reviewer #3: **Yes:** Abera Cheru

[NOTE: If reviewer comments were submitted as an attachment file, they will be attached to this email and accessible via the submission site. Please log into your account, locate the manuscript record, and check for the action link "View Attachments". If this link does not appear, there are no attachment files.]

While revising your submission, please upload your figure files to the Preflight Analysis and Conversion Engine (PACE) digital diagnostic tool, https://pacev2.apexcovantage.com/. PACE helps ensure that figures meet PLOS requirements. To use PACE, you must first register as a user.

Registration is free. Then, login and navigate to the UPLOAD tab, where you will find detailed instructions on how to use the tool. If you encounter any issues or have any questions when using PACE, please email PLOS at [figures@plos.org.](mailto:figures@plos.org) Please note that Supporting Information files do not need this step.
